# Cohort study investigating gout flares and management in UK general practice

**DOI:** 10.1186/s12875-023-02201-7

**Published:** 2023-11-22

**Authors:** Samuel Finnikin, Christian D. Mallen, Edward Roddy

**Affiliations:** 1 Institute of Applied Health Research, University of Birmingham, Birmingham, B15 2TT UK; 2 Primary Care Centre Versus Arthritis, School of Medicine, Keele University, Keele, UK; 3 Haywood Academic Rheumatology Centre, Midlands Partnership NHS Foundation Trust, Stoke-On-Trent, UK

**Keywords:** Gout, Urate lowering therapy, Management, Decision-making

## Abstract

**Background:**

Gout is the most common inflammatory arthritis and is almost exclusively managed in primary care, however the course and severity of the condition is variable and poorly characterised. This research aims improve understanding about the frequency of, and factors associated with, gout flares in the UK and characterise the factors associated with the initiation of ULT.

**Methods:**

Using the Clinical Practice Research Database, patients with a coded incident gout diagnosis without a prior prescription for urate-lowering therapy (ULT) were identified. Gout flares post diagnosis and ULT initiation were identified through prescribing and coded data. Patient characteristics, co-morbidities and co-prescribing were co-variants. Factors associated with gout flares and ULT initiation were analysed using cox-proportional hazard model and logistic regression.

**Results:**

Fifty-one thousand seven hundred eighty-four patients were identified: 18,605 (35.9%, 95%CI 35.5–36.3%) had experienced ≥ 1 recurrent flare, 17.4% (95%CI 17.1–17.8%) within 12 months of diagnosis. Male sex, black ethnicity, higher BMI, heart failure, CKD, CVD and diuretic use were associated with flares, with the highest HR seen with high serum urate levels (≥ 540 µmol/L HR 4.63, 95%CI 4.03–5.31). ULT initiation was associated with similar variables, although higher alcohol intake and older age were associated with lower odds of ULT initiation but were not associated with flares. ULT was initiated in 27.7% (95%CI 27.3–28.0%): 5.7% (95%CI 5.5–5.9%) within 12 months of diagnosis. ULT initiation rates were higher in patients with recurrent flares.

**Conclusion:**

Approximately one in six people with incident gout had a second flare within 12 months. Factors associated with flare recurrence and ULT initiation were similar, but ULT initiation occurred later after diagnosis than previously thought.

**Supplementary Information:**

The online version contains supplementary material available at 10.1186/s12875-023-02201-7.

## Background

Gout is the most common inflammatory arthritis, affecting around 2.5% of the UK population with men having a significantly higher prevalence than women (4.0% Vs 1.1% respectively) [1]. In contrast to other inflammatory arthritides, gout is almost exclusively managed in primary care, [2] however the course and severity of the condition is variable and poorly characterised.

The medical management of gout is typically focused around the treatment of flares, the prevention of flares through lifestyle modification and using urate-lowering therapies (ULT) and the management of comorbidities. [3] In the UK, only around a third of people with gout are initiated on ULT and adherence to treatment is poor [1, 4]. Rates of ULT initiation have been consistent over the last 20 years. A ‘treat to target’ approach is recommended once ULT is initiated [3, 5–7] but achieving target urate levels can be challenging [5, 8]. Suboptimal treatment may result in unnecessary morbidity and increased economic burden both in terms of healthcare costs and the loss of economic productivity amongst individuals during flares [9].

It is known that ULT initiation rates in the UK and internationally are low [1, 10, 11] and, whilst there is a body of research examining adherence to ULT [12], less is known about how clinicians decide to initiate ULT. International guidelines consider flare frequency, co-morbidities (such as CKD), co-prescribing (such as diuretics) and evidence of advanced disease (such as tophi or radiological change) to guide prescribing of ULT [3, 6, 7]. These guidelines correspond with prescribing practice, with CKD, heart failure, obesity, alcohol excess, diuretic therapy, frequent flares, tophi and urolithiasis all increasing the likelihood of being initiated on allopurinol [4, 13]. Clinicians report that flare frequency was the main reason for initiating ULT [14].

A potential barrier to use of ULT is the lack of evidence around, and consensus on, who should be offered this treatment and at what point in the disease course [15]. One of the factors contributing to this is a poor understanding of the natural history of gout including the likelihood and frequency of flares following diagnosis. This causes confusion for both clinicians and patients which adversely influences the use of ULT [16]. Better understanding of the natural history of gout would allow us to predict with greater certainty the likely progression of gout in particular individuals, helping patients and clinicians to understand the condition better. This could facilitate more informed treatment options and shared decision making.

A previous study using routinely collected primary care data [17] found that a third of patients with incident gout had a second gout flare within a mean follow up period of 3.8 years. Ischaemic heart disease (IHD), hypertension and renal failure were associated with a higher risk of flare, whereas allopurinol initiation within 30 days of diagnosis reduced the risk. However, it was not clear whether patients remained on allopurinol throughout the follow up period or whether it was the co-morbidity or the medications prescribed for those co-morbidities (such as diuretics) that mediated the risk of flares.

Investigation of gout flares in a cohort of prevalent cases has shown distinct gout flare trajectories [18] This exploratory study poses the possibility that patients could be stratified by the risk of suffering future flares and, consequently, given more personalised information about their chances of benefiting from ULT.

The purpose of this study was to improve understanding about the frequency and factors associated with gout flares following diagnosis of gout in the UK for patients not prescribed ULT. A secondary aim was to characterise the clinical and non-clinical factors associated with the initiation of ULT for patients diagnosed with gout.

## Methods

This retrospective cohort study comprised patients with incident gout in the Clinical Practice Research Datalink (CPRD) GOLD database [19]. CPRD GOLD contains anonymised primary care records including demographics, coded diagnoses and prescribing data for over 11.3 million patients from 674 practices (around 6.9% of the UK population) and, with over 98% of the UK population registered with a GP [20], it is broadly representative of the demographics of the UK population. The data extraction and cohort selection according to study design were facilitated using the data extraction for epidemiological research (Dexter) tool [21]. Patients aged 20 years and over from practices opting into CPRD GOLD were included in the cohort if they had a first, coded diagnosis of gout (or gout related code such as tophi) between 1st January 2010 and 31st December 2019. Read codes used are shown in Additional file A1. Previous research has validated the coding of gout in UK electronic medical records to an acceptable level (90% accuracy when combined with urate levels and/or prescribing data) and even when the relatively low levels of serum urate testing are considered, the PPV of a primary care diagnosis of gout in the UK has been found to be high at 88.6% [22, 23]. Patients were eligible for inclusion from the study start date or the earliest of either the practice standardisation date (the date at which the practice data is deemed to be of research quality, based on CPRD algorithm) or date the patient registered with the practice plus 1 year (to allow time for records to be transferred); until the earliest of: study end date, end of practice data or death. Patients were excluded if they left the database within 1 year and 30 days of the index date (the date of a coded gout diagnosis) as a minimum of 1 year follow up was required. The follow up period started 30 days after the index date to ensure treatment of the incident flare was not misclassified as a first subsequent flare. Patients were also excluded if they had a prior diagnosis of gout (or related code) or had a prescription of ULT (allopurinol or febuxostat at any dose) prior to, or within 30 days subsequent from, the index date. This latter criterion was to identify and exclude patients started on allopurinol or NSAIDs prophylactically on ULT initiation rather than for a flare. Prescription of ULT was a censoring event and any co-prescribing of NSAID or allopurinol on ULT initiation was not counted as a flare. All flares following the index flare are termed ‘recurrent flares’.

### Variables

The primary outcome variable of interest was gout flares identified by documented episodes of gout subsequent to the index event according to criteria previously used in the literature [17]. Thus, a gout flare following the initial diagnosis was defined as follows: either a recorded prescription of colchicine or a health-care visit recording a gout code (from any source including letters received and coded by practices following Emergency Department or hospital encounters) together with at least one of the following treatments within 7 days of the code (see Additional material A1): intra-articular aspiration, intra-articular injection or corticosteroid, prescription of an oral NSAID or oral corticosteroids. If ULT was prescribed at the same time as a gout flare identifying episode this was not counted as a flare. Ascertainment of flares was performed recursively. Every new follow-up period following a flare included a grace period of 30 days from the date of the flare detected (to allow for full remission of that flare). Covariates included were sex, age, ethnicity, and Index of Multiple Deprivation (IMD – a statistic of relative deprivation used in England [24]) along with the latest recording prior to the index date of: body mass index (BMI), alcohol intake (units per week) and co-morbidities (hypertension, type 1 diabetes mellitus, type 2 diabetes mellitus, heart failure, cardiovascular disease, chronic kidney disease). Co-prescribing was identified within 90 days prior to index date of diuretics or aspirin, and the latest serum urate level prior to or after the index date.

### Statistical analysis

Demographics, co-morbidities, co-prescribing and flare status of patients were described. The difference in the distribution of characteristics between patients who experienced a recurrent flare and those that did not were tested using a Chi-Squared test. The difference between means of continuous variables were compared using the T-test. Multiple testing was accounted for using the Bonferroni Adjustment. The pattern of gout flares was described using the number of flares, time to first recurrent flare, and proportion of people having a first recurrent flare by the end of each complete year of follow up. ULT initiation was described by recurrent flare status and the proportion of patients initiated on ULT within 12 months of diagnosis and within 12 months of first recurrent flare (where applicable).

For diagnoses and medications, missing data was accepted as the absence of that diagnosis or medication. For all other variables, a missing category was employed as missingness could not be assumed to occur at random.

The association between covariates and the frequency of recurrent flares was analysed using logistic regression. A time to event analysis considering multiple events was performed using the Anderson-Gill Cox model with covariates as predictors and flares as the outcome [25, 26] This model was chosen as it allows each flare to be considered as an independent event. A further logistic analysis was undertaken looking at the prescribing of ULT. Variables included all the above in addition to the number of flares experienced. The practice ID was included as a random effect to account for commonalities in prescribing behaviours amongst GPs in the same practice. All statistical analysis were performed using StataSE V17.

## Results

### Cohort description

**Fig. 1:**
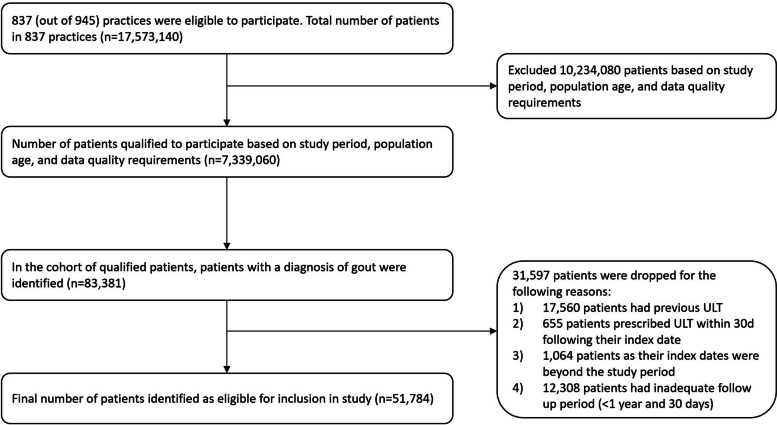
STROBE diagram

In total, 51,784 patients were included in the cohort (see STROBE diagram Fig. 1) with a mean of 4.1 years of follow up (standard deviation 2.1 years) (Table 1). Three-quarters were male and, where ethnicity was recorded, the majority were white. 8,080 patients (15.6%, 95% Confidence Interval 15.3–15.9%) had a BMI between 20–24.9 kg/m^2^ and over half (28,783, 55.6%, 95%CI 55.2–56.0%) had one or more relevant co-morbidity. At least one recurrent flare was identified in 18,605 cohort members (35.9%, 95%CI 35.5–36.3%). All variables apart from type 1 diabetes were significantly different between the patients who had at least one recurrent flare and those who did not flare. Patients who did not experience a recurrent flare during follow-up were more likely to be female, younger, of white ethnicity, normal BMI, drink less alcohol, not have a co-morbidity or be prescribed aspirin or a diuretic, and have lower serum urate. A large proportion of the cohort did not have a serum urate measured at any time (21,062, 95%CI 40.3–41.1%) with females having a slightly lower rate of missing urate than males (38.0% Vs 41.3% respectively).

**Table 1:** Characteristics of the total cohort and split by flare status (n(%) unless otherwise stated)

		All	No flares	Flares	*P* value
N		51784 (100)	33179 (64.1)	18605 (35.9)	-
Follow up (years, mean (S.D.))		4.1 (2.1)	3.8 (2.0)	4.6 (2.1)	< 0.001^a^
Sex	Male	37873 (73.1)	23373 (70.5)	14500 (77.9)	< 0.001^b^
	Female	13909 (26.8)	9805 (29.6)	4,104 (22.1)	
	Missing	2 (0.0)	1 (0.0)	1 (0.0)	
Age category (years)	20–49	12052 (23.3)	7939 (23.9)	4113 (22.1)	< 0.001^b^
	50–59	9894 (19.1)	6564 (19.8)	3330 (17.9)	
	60–69	11980 (23.1)	7652 (23.1)	4328 (23.1)	
	70–79	10978 (21.2)	6600 (19.9)	4378 (23.5)	
	≥ 80	6880 (13.3)	4424 (13.3)	2456 (13.2)	
Ethnicity	White	22693 (43.8)	14747 (44.5)	7946 (42.7)	< 0.001^b^
	Black	4089 (7.9)	2495 (7.5)	1594 (8.6)	
	South Asian	692 (1.3)	473 (1.4)	219 (1.2)	
	Mixed Race	92 (0.2)	63 (0.2)	29 (0.2)	
	Other	985 (1.9)	665 (2.0)	320 (1.7)	
	Missing	23233 (44.9)	14736 (44.4)	8497 (45.7)	
Body Mass Index (kg/m^2^)	< 20	770 (1.5)	590 (1.8)	180 (1.0)	< 0.001^b^
	20–24.9	8080 (15.6)	5610 (16.9)	2470 (13.3)	
	25–29.9	19014 (36.7)	12074 (36.4)	6940 (37.3)	
	30–34.9	12813 (24.7)	7976 (24.0)	4837 (26.0)	
	≥ 35	7402 (14.3)	4486 (13.5)	2916 (15.7)	
	Missing	3705 (7.2)	2443 (7.4)	1262 (6.8)	< 0.001^b^
Alcohol consumption (units/week)	0	4580 (8.8)	2880 (8.7)	1700 (9.1)	
	1–9	9310 (18.0)	6074 (18.3)	3236 (17.4)	
	10–24	9346 (18.1)	5855 (17.7)	3491 (18.8)	
	25–42	3336 (6.4)	2049 (6.2)	1287 (6.9)	
	> 42	1713 (3.3)	1069 (3.2)	644 (3.5)	
	Missing	23499 (45.4)	15252 (46.0)	8247 (44.3)	
Type 1 Diabetes	Yes	274 (0.5)	181 (0.5)	93 (0.5)	0.62
Type 2 Diabetes	Yes	6189 (12.0)	3813 (11.5)	2376 (12.8)	< 0.001^b^
Hypertension	Yes	24024 (46.4)	14628 (44.1)	9396 (50.5)	< 0.001^b^
Heart failure	Yes	2877 (5.6)	1386 (4.2)	1491 (8.0)	< 0.001^b^
CVD	Yes	11302 (21.8)	6519 (19.7)	4783 (25.7)	< 0.001^b^
Diuretic	Yes	16116 (31.1)	9293 (28.0)	6823 (36.7)	< 0.001^b^
Aspirin	Yes	8827 (17.1)	5221 (15.7)	3606 (19.4)	< 0.001^b^
CKD	No CKD	40664 (78.5)	27034 (81.5)	13630 (73.3)	< 0.001^b^
	Stage 3	9786 (18.9)	5413 (16.3)	4373 (23.5)	
	Stage 4	911 (1.8)	472 (1.4)	439 (2.4)	
	Stage 5	423 (0.8)	260 (0.9)	163 (0.8)	
Serum Urate category (µmol/L)	< 360	3981 (7.7)	3461 (10.4)	520 (2.8)	< 0.001^b^
	360–419	5075 (9.8)	3689 (11.1)	1386 (7.5)	
	420–479	9254 (17.9)	5922 (17.9)	3335 (17.9)	
	480–539	7202 (13.9)	4074 (12.3)	3128 (16.8)	
	≥ 540	5210 (10.1)	2437 (7.6)	2773 (14.9)	
	Missing	21062 (40.7)	13596 (41.0)	7466 (40.13)	
ULT started	Yes	14318 (27.7)	5302 (16.0)	9016 (48.5)	< 0.001^b^
Time to ULT (days, mean and S.D.)		1036 (731)	800 (679)	1174 (725)	< 0.001^a^

^a^T-test, significant at *p*< 0.003; ^b^Wilcoxon rank-sum test, significant at *p*< 0.003

### Gout flares

**Fig. 2:**
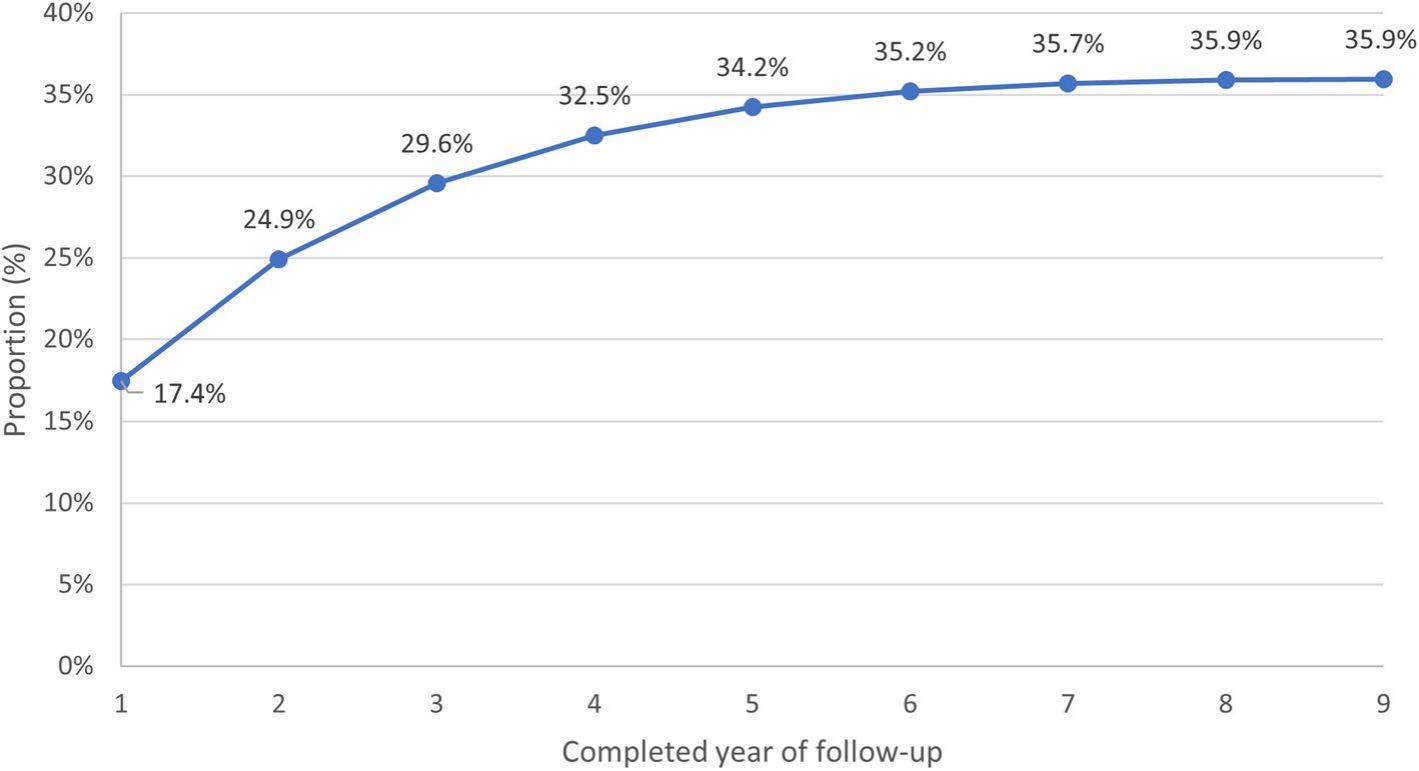
Proportion of cohort experiencing at least one recurrent flare by the end of each year

Overall, 39,889 flares were identified (mean 2.14 flares per patient experiencing recurrent flare). 17.4% (95%CI 17.1–17.8%) of patients experienced a first recurrent flare within 12 months of first diagnosis (Fig. 2). The median time to first recurrent flare was 385 days (IQR 136–871 days) and time to second recurrent flare was 585 days (IQR 264–1161 days) The majority of flares were identified through the prescription of colchicine (32,167, 80.6%, 95%CI 80.3–81.0%) followed by a consultation for gout with an NSAID prescription (6,535, 16.4%, 95%CI 16.0–16.8%), prednisolone prescription (1,175, 3.0%, 95%CI 2.8–3.1%), joint injection (8, 0.02% 95%CI 0.01–0.04%) and joint aspiration (4, 0.01%, 95%CI 0.00–0.03%).

In the time to event analysis, flares were less likely in women (HR 0.69, 95%CI 0.66–0.73) and more likely in those of black ethnicity (1.16, 95% CI 1.08–1.25) and with a higher BMI (Table 2). Heart failure, CVD, CKD stages 3 and 4, and diuretic use, but not hypertension, diabetes or aspirin use, were associated with an increased risk of flares. Higher serum urate had the most impact on the risk of flares with the hazard ratio for the highest category (≥ 540 µmol/l) being 4.63 (95%CI 4.03–5.31).

**Table 2:** Association between variables and flares (multivariate Cox proportional-hazards model Anderson-Gill method) and ULT initiation (multivariate logistic regression)

		Hazard ratio for flares (95%CI)	*P*	Odds Ratio for ULT (95%CI)	*P*
Age (25–49 reference)	50–59	0.99 (0.93–1.06)	0.827	0.96 (0.90–1.02)	0.168
	60–69	1.07 (1.00–1.14)	0.037	**0.87 (0.82–0.94)**	< 0.001
	70–79	1.10 (1.02–1.18)	0.011	**0.73 0.68–0.79)**	< 0.001
	80 +	1.00 (0.92–1.09)	0.988	**0.50 (0.45–0.55)**	< 0.001
Sex (ref male)	Female	**0.69 (0.66–0.73)**	< 0.001	**0.83 (0.78–0.88)**	< 0.001
Ethnicity (ref White)	Black	**1.16 (1.08–1.25)**	< 0.001	1.06 (0.97–1.15)	0.193
	South Asian	1.00 (0.85–1.18)	0.967	0.83 (0.69–1.00)	0.047
	Mixed Race	0.86 (0.60–1.24)	0.419	0.91 (0.58–1.41)	0.658
	Other	1.13 (0.98–1.30)	0.085	0.97 (0.83–1.14)	0.735
	Missing	1.01 (0.97–1.05)	0.601	**0.93 (0.88–0.99)**	0.017
IMD (ref 1st (least deprived) decile)	2	1.07 (0.97–1.17)	0.159	1.00 (0.89–1.12)	0.967
	3	1.00 (0.91–1.09)	0.953	1.06 (0.93–1.20)	0.381
	4	1.08 (0.98–1.19)	0.142	1.10 (0.97–1.25)	0.121
	5	**1.14 (1.02–1.28)**	0.020	**1.18 (1.02–1.35)**	0.025
	Missing	**1.14 (1.06–1.22)**	< 0.001	**1.36 (1.22–1.51)**	< 0.001
BMI kg/m^2^ (ref 20–24.9)	< 20	0.95 (0.77–1.17)	0.647	0.85 (0.69–1.05)	0.126
	25–29.9	**1.10 (1.04–1.17)**	0.001	**1.21 (1.13–1.30)**	< 0.001
	30–34.9	**1.18 (1.10–1.25)**	< 0.001	**1.27 (1.18–1.37)**	< 0.001
	35 +	**1.36 (1.27–1.47)**	< 0.001	**1.34 (1.24–1.45)**	< 0.001
	Missing	**1.14 (1.04–1.25)**	0.006	1.11 (1.00–1.23)	0.042
Alcohol units per week (ref 0)	1–9	0.98 (0.91–1.06)	0.668	**0.77 (0.70–0.85)**	< 0.001
	10–24	0.98 (0.91–1.06)	0.606	**0.81 (0.74–0.89)**	< 0.001
	25–42	1.09 (0.99–1.19)	0.091	**0.84 (0.75–0.94)**	0.003
	> 42	1.11 (0.97–1.26)	0.117	**0.79 (0.69–0.91)**	0.001
	Missing	0.99 (0.92–1.06)	0.784	**0.82 (0.75–0.89)**	< 0.001
Hypertension		**1.09 (1.03–1.14)**	0.001	1.03 (0.98–1.08)	0.265
Type 1 diabetes		0.96 (0.73–1.25)	0.757	0.93 (0.69–1.25)	0.626
Type 2 diabetes		1.04 (0.97–1.10)	0.255	**0.86 (0.80–0.92)**	< 0.001
Heart Failure		**1.38 (1.27–1.49)**	< 0.001	**1.44 (1.29–1.59)**	< 0.001
CVD		**1.22 (1.15–1.31)**	< 0.001	0.97 (0.91–1.04)	0.433
Diuretics		**1.18 (1.12–1.24)**	< 0.001	**1.27 (1.20–1.35)**	< 0.001
Aspirin		0.96 (0.91–1.03)	0.242	**1.11 (1.04–1.19)**	0.002
CKD (ref none)	CKD Stage 3	**1.33 (1.26–1.40)**	< 0.001	**1.51 (1.42–1.60)**	< 0.001
	CKD stage 4	**1.37 (1.19–1.56)**	< 0.001	**1.87 (1.59–2.21)**	< 0.001
	CKD stage 5	1.08 (0.86–1.35)	0.493	**1.55 (1.23–1.96)**	< 0.001
Serum urate µmol/L (ref < 360)	360–419	**1.91 (1.65–2.22)**	< 0.001	**2.74 (2.34–3.21)**	< 0.001
	420–479	**2.64 (2.31–3.01)**	< 0.001	**4.49 (3.85–5.23)**	< 0.001
	480–539	**3.43 (3.00–3.92)**	< 0.001	**6.83 (5.86–7.96)**	< 0.001
	≥ 540	**4.63 (4.03–5.31)**	< 0.001	**11.16 (9.56–13.04)**	< 0.001
	Missing	**2.62 (2.30–2.98)**	< 0.001	**4.02 (3.45–4.69)**	< 0.001
Number of flares		-	-	**1.43 (1.38–1.48)**	< 0.001

Bold text highlights statistical significance

### ULT initiation

**Fig. 3:**
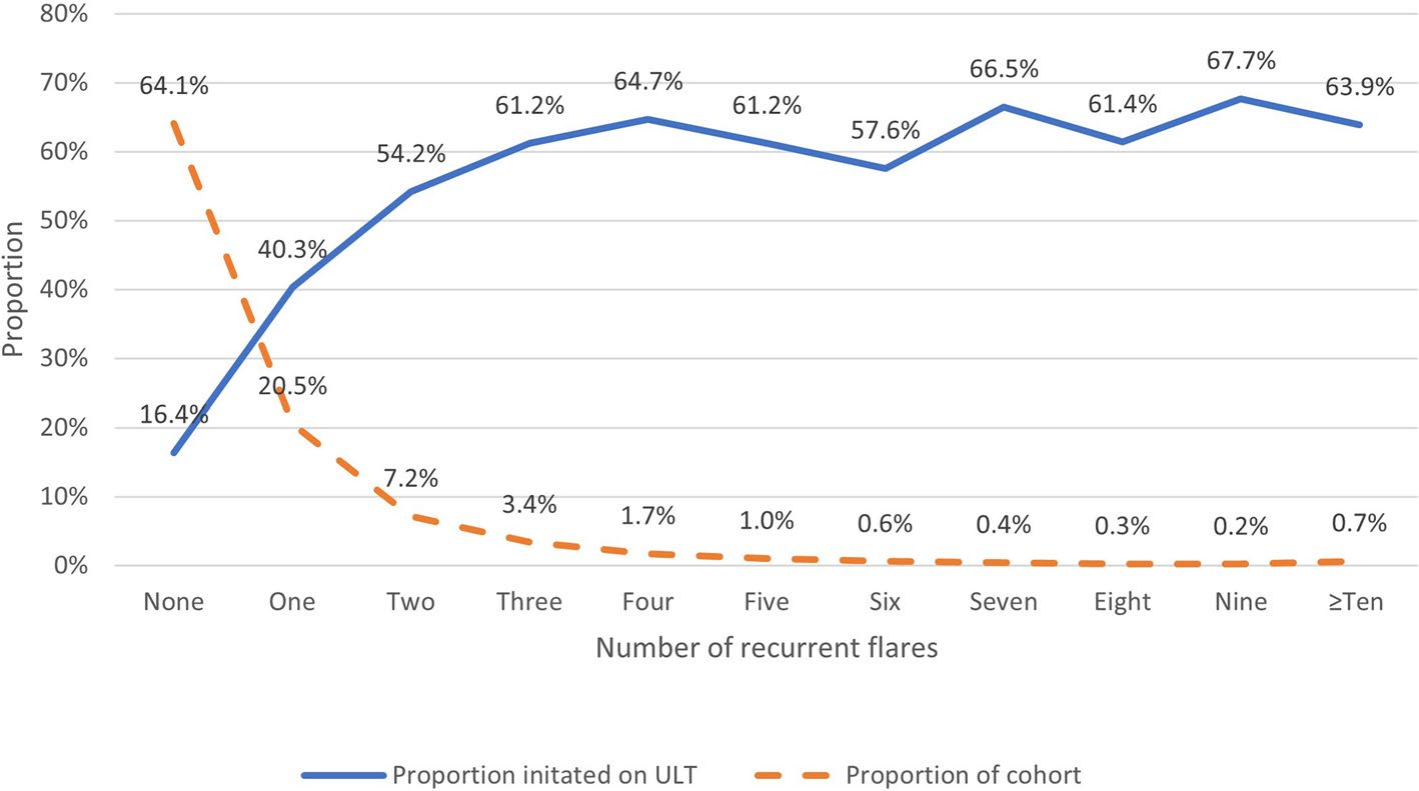
Number of recurrent flares experienced and ULT initiation levels per number of recurrent flares

ULT was initiated in 14,318 individuals (27.7%, 95%CI 27.3–28.0%), with a higher initiation rate in patients who experienced one or more recurrent flares (48.5% (95%CI 47.7–79.2%) vs 16.0% (95%CI 15.6–16.4%) in those with no recurrent flare, *p* < 0.001). Only 5.7% (95%CI 5.5–5.9%) of the cohort (*n* = 2,944) were initiated on ULT within 12 months of diagnosis, 5.4% (95%CI 5.2–5.6%, *n* = 1,790) of patients without a recorded recurrent flare and 6.2% (95CI 5.9–6.6%, *n* = 1,154) of those with one or more recurrent flare. Of the 18,605 people experiencing one or more recurrent flares, 3,590 (19.3%, 95%CI 18.7–19.9%) were initiated on ULT within 12 months of the first recurrent flare. The mean time between diagnosis and ULT initiation was shorter for people who did not experience any recurrent flares than those who did (800 days vs 1174 days, *P* < 0.001). Figure 3 shows the proportion of people initiated on ULT according to the number of recurrent flares experienced. The proportion levelled out after three recurrent flares with approximately a third of people not being initiated on ULT regardless of the number of flares.

The odds ratios for initiating ULT were highest for higher serum urate levels, but were also increased in those with CKD, diuretic and aspirin use, heart failure and higher BMI. All levels of alcohol consumption were associated with a lower odds ratio for ULT initiation as were type 2 diabetes, female sex and older age.

## Discussion

### Main findings

In this large cohort of patients diagnosed with gout who did not commence ULT, 35.9% of patients experienced a recurrent flare during a median follow-up period of 4.1 years. Approximately one in six (of these recurrent flares) occurred within 12 months of diagnosis. After 5 years of follow-up, first recurrent flares were unlikely. 14,318 of the cohort were initiated on ULT (27.7%, 95%CI 27.3–28.0%). A significantly higher proportion of ULT initiations were to people who had evidence of a recurrent flare after diagnosis (48.5% Vs 16.0%) although there may have been unrecorded flares before or after diagnosis that influenced decision-making. Of the people who were initiated on ULT, the time between diagnosis and ULT initiation was considerably lower in people who did not experience a recurrent flare which is likely due to the therapeutic effect of ULT.

When comparing factors associated with the likelihood of a flare with factors associated with ULT initiation we found that age did not seem to influence flare risk, but the odds of being prescribed ULT decreased significantly in older age groups indicating that older people may not be getting an opportunity to benefit from ULT to the same extent as younger people. Prescribing omissions in other areas of prevention including cardiovascular disease, anticoagulation and anti-osteoporotic drugs been found to be more likely in older people and those with polypharmacy and multimorbidity [27, 28] Females were less likely to experience recurrent flares and also had lower odds of being initiated on ULT, even when the number of flares is adjusted for in the model. This could be that clinicians have a heuristic that females experience less frequent gout flares and therefore are less likely to initiate ULT. Similar congruence was found with higher BMI both increasing the risk of recurrent flares and the odds of ULT initiation. Interestingly, no association between alcohol consumption and recurrent flares was identified even in the univariate analysis (Additional file A2), and higher alcohol consumption was associated with lower odds of being initiated on ULT. Alcohol consumption is usually thought to be associated with gout flares and indeed this has been shown in previous studies [17, 29].

### Comparison with existing literature

Our findings are similar to those of Rothenbacher et al. who reported that 36.9% of patients experienced a recurrent flare over a mean follow-up of 3.8 years and that the median time to first recurrent flare was 385 days [17]. Rothenbacher et al. found male sex, higher levels of alcohol consumption, higher BMI, and a history of IHD, hypertension and CKD were associated with time to first post-diagnosis flare. In our analysis, which considered multiple events, the same risk factors were identified with the exception of alcohol consumption. Serum urate was also included in our model and produced the highest hazard ratios. The association between raised serum urate levels and gout flares has been well characterised in the literature previously [30].

The use of ULT is known to be low in the UK with prescribing rates of 37.6% in people with prevalent gout and 27.3% of people being initiated on gout within 12 months of diagnosis [1]. A very similar proportion of people being initiated on ULT within 12 months of diagnosis (28.9%) was found in a separate cohort [4] and other observational data shows that 40% of patients are initiated on ULT over a follow-up period of 31 months [13] and 14% of patients were initiated on ULT within 12 m of a gout flare (excluding initiation during flare) [10]. We observed much lower rates of ULT initiation with only 5.7% (95%CI 5.5–5.9%) of patients being initiated on ULT within 12 months of diagnosis. However, overall initiation rates of 27.7% in our cohort were consistent with the annual frequency of allopurinol use of 25.3–29.5% reported by Mikuls et al [31]. This disparity in the 12 month initiation rate but consistency in overall ULT initiation can be explained due to the exclusion of patients previously prescribed ULT in our definition of gout diagnosis. Other cohorts did not exclude patients previously prescribed ULT [1, 4]. Without this exclusion criteria, we would have included approximately 18,000 patients out of about 70,000 patients with adequate follow up period. This would have represented around 26% of the cohort and these patients would have been likely to have been prescribed ULT in the first 12 months post diagnosis as it was already being prescribed. This would significantly inflate our 12-month ULT initiation rates in line with that previously reported. This strongly suggests that previous incident gout cohorts have been contaminated with prevalent gout cases due to delays in diagnostic coding. One other cohort also excluded patients who had been prescribed ULT (although limited to the 12 months prior to study entry), reporting a mean time to the first ULT prescription of 927 days [32] which is comparable with our corresponding figure of 1036 days. We also excluded patients who were prescribed ULT around the time of diagnosis. These patients may have been included in other cohorts. However, initiating ULT at the time of first gout flare is not standard practice in the UK and these patients may have had previous uncoded flares influencing decision making.

In terms of predictors of ULT initiation; CKD, diuretic use and being overweight have previously been observed to be associated with ULT initiation [13] but this study found that males were less likely to be prescribed ULT in contrast to our findings. This could be explained by additional co-variants in our model, notably serum urate which is known to be higher in males [33]. We found that around 40% of patients did not have a serum urate levels recorded in there record which is similar to previous estimates missing serum urate levels. [23].

The majority of flares in this cohort were managed with colchicine (80.6%) but previous research has shown flares in the UK are usually managed with NSAIDs, with colchicine making up a minority of prescriptions [10, 17]. This could, in part, be due to increasing attention being paid to safety concerns regarding NSAIDS, [34] and also due to the difference in the way that flares were ascertained with NSAID-treated flares having to be associated with a gout code, whereas colchicine flares did not. Many NSAID prescriptions in the cohort were not associated with a gout code and therefore did not get identified as a flare (data not reported). Only a quarter of colchicine prescriptions were associated with a gout code. If similar coding practices were seen with NSAIDs, then there were potentially 17,000 extra flares treated with NSAIDs. However, this would change definition of a gout flare previously established in the literature and prevent accurate comparison. Regardless, colchicine would be the most common medication used to manage gout flares. The disparity with older research could also represent a shift in prescribing practices. This shift was also observed in an analysis of 1308 consultations for gout flares between 2005 and 2015 with the proportion of patients prescribed colchicine rising from 15.5% in 2005 to 31.2% in 2015, and NSAID prescriptions declining from 38.1% to 15.0% over the same period [35]. A trend towards lower overall NSAID prescribing as has also been observed in patients with CVD and in managing osteoarthritis [36, 37].

### Strengths and limitations

This large cohort uses established methods to update what is known about the management of gout in the UK. Due to the nature of the dataset, and almost universal use of electronic prescribing, we can be confident that prescribing data are accurate and generalisable although it is acknowledged that the number of practices contributing to this dataset was declining during the study period due to a move away from the electronic patient record software utilised. Excluding patients with ULT prescriptions prior to gout diagnosis allows more confidence that the index coding represents incident gout than has been previously described resulting in a more accurate description of ULT initiation and flares post-diagnosis. However, only one coded gout diagnosis was required for inclusion, raising the possibility of misclassification.

In the analysis of gout flares, accounting for multiple events instead of only the first recurrent flare allows for different gout trajectories [18] to contribute to the model. This improves the accuracy of our understanding of the factors associated with gout flares which could help clinicians and patients make more informed decisions regarding initiation of ULT.

One of the main limitations of this and all other observational studies using routinely collected data to investigate gout flares is that some flare events will be managed by patients without seeking medical input meaning there will be under-reporting of flare events. However the other main method of characterising gout flares would be through prospective self-reporting and this is not without challenge either [38]. It is likely that flare under-reporting due to self-management is less of a problem earlier in the course of the condition as patients may take time build confidence to manage their flares. There is also the possibility that unrecognised or unmeasured confounders were not included in the model which may affect the relationships reported.

In identifying gout flares it is important to note that colchicine may (rarely) be used in conditions other than gout and it is possible that NSAIDs or oral steroids were prescribed for other indications. The temporal association of gout codes with NSAIDs and oral steroid would reduce this risk. It is worth noting that other methods of defining flares also require this temporal coding association for colchicine related events as well [39]. However, since colchicine is almost exclusively used to manage gout, we felt it was unnecessary to require a corresponding gout code in the colchicine related flare definition, and to do so would have excluded a large proportion of true flares that are treated with colchicine.

## Conclusions

Many people with gout are not initiated on ULT and, although this has been highlighted in the past, by creating a more accurate incident cohort we have estimated the rate of ULT initiation after diagnosis to be considerably lower than previously reported. This suggests that many more people are not getting the opportunity to reduce their risk of flares than previously thought. Two thirds of people may not have a recurrent flare in the years following diagnosis, but those who do suffer recurrent attacks could potentially be initiated on ULT earlier in the progression of their disease. With one in six people experiencing a recurrent flare within 12 months of diagnosis, the potential benefits of ULT should be discussed early in the disease course to allow patients to consider their options through shared decision making. This study should help inform decision making by giving clinicians more insight into the risk of recurrent flares, with a particular focus on serum urate levels as the highest risk factor for recurrent flares. However, serum urate was not recorded for 41% of the cohort. Ensuring that all patients with gout have their serum urate measured soon after diagnosis and using this information in to inform ULT decision making could improve the management of this debilitating condition.

### Supplementary Information

**Additional file 1.** 

**Additional file 2.** 

## Data Availability

The anonymised, coded data used in these analyses were provided by CPRD following approval by their Research Data Governance committee. The data are available on request from CPRD. Full code lists used in these analyses are available on request from the corresponding author.

## Abbreviations

ULT: Urate Lowering Therapy
IHD: Ischaemic Heart Disease
CPRD: Clinical Practice Research Datalink
GP: General Practitioner
IMD: Index of Multiple Deprivation
NSAIDs: Non-Steroidal Anti-Inflammatory Drugs
BMI: Body Mass Index
CKD: Chronic Kidney Disease
CVD: Cardiovascular Disease

## References

[1] Kuo C-F, Grainge MJ, Mallen C, Zhang W, Doherty M (2015). Rising burden of gout in the UK but continuing suboptimal management: a nationwide population study. Ann Rheum Dis.

[2] National Institute for Health and Care Excellence (2020). Guideline scope Gout: diagnosis and management.

[3] National Institute for Health and Care Excellence (2022). Gout: diagnosis and management.

[4] Russell MD, Rutherford AI, Ellis B, Norton S, Douiri A, Gulliford MC (2022). Management of gout following 2016/2017 European (EULAR) and British (BSR) guidelines: an interrupted time-series analysis in the United Kingdom. Lancet Reg Health Eur.

[5] Keenan RT (2017). Limitations of the current standards of care for treating gout and crystal deposition in the primary care setting: a review. Clin Ther.

[6] Fitzgerald JD, Dalbeth N, Mikuls T, Brignardello-Petersen R, Guyatt G, Abeles AM (2020). 2020 American College of Rheumatology guideline for the management of gout. Arthritis Care Res.

[7] Richette P, Doherty M, Pascual E, Barskova V, Becce F, Castañeda-Sanabria J (2017). 2016 updated EULAR evidence-based recommendations for the management of gout. Ann Rheum Dis.

[8] Becker MA, Fitz-Patrick D, Choi HK, Dalbeth N, Storgard C, Cravets M (2015). An open-label, 6-month study of allopurinol safety in gout: the LASSO study. Semin Arthritis Rheum.

[9] Rai SK, Burns LC, De Vera MA, Haji A, Giustini D, Choi HK (2015). The economic burden of gout: a systematic review. Semin Arthritis Rheum.

[10] Roddy E, Mallen CD, Hider SL, Jordan KP (2010). Prescription and comorbidity screening following consultation for acute gout in primary care. Rheumatology.

[11] Proudman C, Lester SE, Gonzalez-Chica DA, Gill TK, Dalbeth N, Hill CL (2019). Gout, flares, and allopurinol use: a population-based study. Arthritis Res Ther.

[12] Yin R, Li L, Zhang G, Cui Y, Zhang L, Zhang Q (2018). Rate of adherence to urate-lowering therapy among patients with gout: a systematic review and meta-analysis. BMJ Open.

[13] Clarson LE, Hider SL, Belcher J, Roddy E, Mallen CD (2017). Factors influencing allopurinol initiation in primary care. Ann Fam Med.

[14] Rai SK, Choi HK, Choi SH, Townsend AF, Shojania K, De Vera MA (2018). Key barriers to gout care: a systematic review and thematic synthesis of qualitative studies. Rheumatology.

[15] Drug and Therapeutics Bulletin. Latest guidance on the management of gout. BMJ. 2018:k2893. 10.1136/bmj.k2893 30021789

[16] Richardson JC, Liddle J, Mallen CD, Roddy E, Hider S, Prinjha S (2016). A joint effort over a period of time: factors affecting use of urate-lowering therapy for long-term treatment of gout. BMC Musculoskelet Disord.

[17] Rothenbacher D, Primatesta P, Ferreira A, Cea-Soriano L, Rodríguez LAG (2011). Frequency and risk factors of gout flares in a large population-based cohort of incident gout. Rheumatology.

[18] Watson L, Belcher J, Nicholls E, Muller S, Mallen C, Roddy E (2020). Latent class growth analysis of gout flare trajectories: a three-year prospective cohort study in primary care. Arthritis Rheumatol.

[19] Herrett E, Gallagher AM, Bhaskaran K, Forbes H, Mathur R, Van Staa T (2015). Data resource profile: clinical practice research datalink (CPRD). Int J Epidemiol.

[20] England NHS (2012). Attribution Dataset GP Registered Populations Scaled to ONS Population Estimates—2011.

[21] Gokhale KM, Chandan JS, Toulis K, Gkoutos G, Tino P, Nirantharakumar K (2021). Data extraction for epidemiological research (DExtER): a novel tool for automated clinical epidemiology studies. Eur J Epidemiol.

[22] Meier CR, Jick H (1997). Omeprazole, other antiulcer drugs and newly diagnosed gout. Br J Clin Pharmacol.

[23] Watson L, Muller S, Roddy E. Primary care diagnosis of gout compared to a primary care diagnostic rule for gout and to classification criteria. J Rheumatol. 2019;46(11):1542-. 10.3899/jrheum.190495 31308214

[24] Noble S, McLennan D, Noble M, Plunkett E, Gutacker N, Silk M, et al. The English indices of deprivation 2019. CLG Ministry of Housing, Editor London. 2019.

[25] Cleves M. Analysis of multiple failure-time data with stata. Stata Technical Bull. 2000;9.

[26] Andersen PK, Gill RD (1982). Cox's regression model for counting processes: a large sample study. Ann Stat.

[27] Gorup EC, Šter MP (2017). Number of medications or number of diseases: what influences underprescribing?. Eur J Clin Pharmacol.

[28] Lombardi F, Paoletti L, Carrieri B, Dell’Aquila G, Fedecostante M, Di Muzio M (2021). Underprescription of medications in older adults: causes, consequences and solutions—a narrative review. Eur Geriatr Med.

[29] Wang M, Jiang X, Wu W, Zhang D (2013). A meta-analysis of alcohol consumption and the risk of gout. Clin Rheumatol.

[30] Shiozawa A, Szabo SM, Bolzani A, Cheung A, Choi HK (2017). Serum uric acid and the risk of incident and recurrent gout: a systematic review. J Rheumatol.

[31] Mikuls TR (2005). Gout epidemiology: results from the UK general practice research database, 1990–1999. Ann Rheum Dis.

[32] Abhishek A, Cipolletta E, Nakafero G, Avery AJ, Mamas M, Tata LJ (2022). Serum urate outcomes of treat-to-target urate lowering treatment: results of a nationwide cohort study from 1997 to the COVID-19 pandemic using data from the Clinical Practice Research Datalink. Ann Rheum Dis.

[33] Zhu Y, Pandya BJ, Choi HK (2011). Prevalence of gout and hyperuricemia in the US general population: the National Health and Nutrition Examination Survey 2007–2008. Arthritis Rheum.

[34] Medicines and Healthcare products Regulatory Agency. Diclofenac: new contraindications and warnings 2014 [Cited 24.10.2023]. Available from: https://www.gov.uk/drug-safety-update/diclofenac-new-contraindications-and-warnings.

[35] Padmanabhan N, Muller S, Mallen C, Roddy E. P149 Prescription for consultations with gout flare in primary care: an observational study. Rheumatology. 2022;61(Supplement_1):keac133. 48.

[36] Zeng C, Zhang W, Doherty M, Persson MS, Mallen C, Swain S (2021). Initial analgesic prescriptions for osteoarthritis in the United Kingdom, 2000–2016. Rheumatology.

[37] Chen Y, Bedson J, Hayward RA, Jordan KP (2018). Trends in prescribing of non-steroidal anti-inflammatory drugs in patients with cardiovascular disease: influence of national guidelines in UK primary care. Fam Pract.

[38] Teoh N, Gamble GD, Horne A, Taylor WJ, Palmano K, Dalbeth N (2019). The challenges of gout flare reporting: mapping flares during a randomized controlled trial. BMC Rheumatol.

[39] Cipolletta E, Tata LJ, Nakafero G, Avery AJ, Mamas MA, Abhishek A (2022). Association between gout flare and subsequent cardiovascular events among patients with gout. JAMA.

