# Supplementary material for: Cohort study investigating gout flares and management in UK general practice
Source: BMC Prim Care. 2023 Nov 22;24:246. doi: 10.1186/s12875-023-02201-7 (PMC10664696; doi:10.1186/s12875-023-02201-7)
Supplement: Supplementary file 1 — Additional file 1. [file 12875_2023_2201_MOESM1_ESM.pdf]

Cohort study investigating gout flares and management in UK general practice: Additional File A1

Univariate analysis: Association between variables and flares (Cox proportional-hazards model Anderson-Gill method) and ULT initiation (logistic regression)

		Hazard ratio for flares (95%CI)	P	Odds Ratio for ULT (95%CI)	P
Age (25-49 reference)	50-59	1.00 (0.94-1.07)	0.926	0.96 (0.91-1.02)	0.181
	60-69	**1.18 (1.11-1.25)**	<0.001	0.97 (0.93-1.04)	0.644
	70-79	**1.37 (1.30-1.46)**	<0.001	1.03 (0.97-1.09)	0.408
	80+	**1.38 (1.29-1.48)**	<0.001	**0.82 (0.77-0.88)**	<0.001
Sex (ref male)	Female	**0.78 (0.74-0.81)**	<0.001	**0.77 (0.74-0.81)**	<0.001
Ethnicity (ref White)	Black	**1.18 (1.10-1.27)**	<0.001	1.12 (1.03-1.21)	0.004
	South Asian	0.92 (0.78-1.09)	0.337	**0.78 (0.67-0.95)**	0.011
	Mixed Race	0.76 (0.51-1.13)	0.178	0.83 (0.54-1.27)	0.389
	Other	1.03 (0.90-1.19)	0.639	0.94 (0.81-1.08)	0.391
	Missing	1.03 (1.00-1.07)	0.153	**1.00 (0.95-1.06)**	0.960
IMD (ref 1st (least deprived) decile)	2	1.11 (1.01-1.22)	0.031	1.04 (0.94-1.16)	0.429
	3	1.02 (1.01-1.24)	0.644	1.09 (1.05-1.31)	0.145
	4	1.12 (1.01-1.24)	0.026	1.17 (1.05-1.31)	0.006
	5	**1.24 (1.10-1.39)**	<0.001	**1.29 (1.13-1.48)**	<0.001
	Missing	**1.19 (1.11-1.28)**	<0.001	**1.49 (1.36-1.65)**	<0.001
BMI kg/m^2 (ref 20-24.9)	<20	0.90 (0.73-1.11)	0.309	0.78 (0.64-0.95)	0.014
	25-29.9	**1.19 (1.12-1.27)**	<0.001	**1.38 (1.29-1.47)**	<0.001
	30-34.9	**1.32 (1.24-1.41)**	<0.001	**1.58 (1.48-1.69)**	<0.001
	35+	**1.53 (1.42-1.64)**	<0.001	**1.83 (1.70-1.97)**	<0.001
	Missing	1.09 (1.00-1.20)	0.061	**1.30 (1.19-1.43)**	<0.001
Alcohol units per week (ref 0)	1-9	0.98 (0.91-1.06)	0.673	**0.72 (0.66-0.80)**	<0.001
	10-24	0.97 (0.90-1.04)	0.393	**0.79 (0.72-0.87)**	<0.001
	25-42	1.06 (0.97-1.17)	0.203	0.86 (0.77-0.96)	0.007
	>42	1.07 (0.94-1.22)	0.292	**0.81 (0.74-0.92)**	0.002
	Missing	1.02 (0.95-1.10)	0.512	**0.81 (0.74-0.87)**	<0.001
Hypertension		**1.37 (0.32-1.43)**	<0.001	**1.26 (1.21 (1.31)**	<0.001
Type 1 diabetes		1.23 (0.94-1.62)	0.136	1.12 (0.86-1.46)	0.407
Type 2 diabetes		**1.31 (1.24-1.39)**	<0.001	1.09 (1.02-1.15)	0.005
Heart Failure		**2.25 (2.11-2.40)**	<0.001	**2.08 (1.93-2.24)**	<0.001
CVD		**1.62 (1.55-1.70)**	<0.001	**1.30 (1.24-1.36)**	<0.001
Diuretics		**1.60 (1.54-1.67)**	<0.001	**1.59 (1.53-1.66)**	<0.001
Aspirin		**1.33 (1.27-1.40)**	<0.001	**1.24 (1.17-1.30)**	<0.001
CKD (ref none)	CKD Stage 3	**1.69 (1.62-1.77)**	<0.001	**1.71 (1.63-1.79)**	<0.001
	CKD stage 4	**2.08 (1.83-2.35)**	<0.001	**2.52 (2.20-2.90)**	<0.001
	CKD stage 5	1.16 (0.93-1.46)	0.173	**1.80 (1.44-2.25)**	<0.001
Serum urate µmol/L (ref <360)	360-419	**2.17 (1.89-2.53)**	<0.001	**3.23 (2.82-3.82)**	<0.001
	420-479	**3.16 (2.77-3.61)**	<0.001	**5.97 (5.15-6.93)**	<0.001
	480-539	**4.34 (3.79-4.95)**	<0.001	**9.99 (8.61-11.6)**	<0.001
	>=540	**6.70 (5.86-7.67)**	<0.001	**19.0 (16.4-22.0)**	<0.001
	Missing	**3.15 (2.77-3.59)**	<0.001	**5.62 (4.84-6.53)**	<0.001
Number of flares				**1.54 (1.49-1.60)**	<0.001

Bold indicates significance at level of p<0.003
