# Supplementary material for: Cohort study investigating gout flares and management in UK general practice
Source: BMC Prim Care. 2023 Nov 22;24:246. doi: 10.1186/s12875-023-02201-7 (PMC10664696; doi:10.1186/s12875-023-02201-7)
Supplement: Supplementary file 2 — Additional file 2. [file 12875_2023_2201_MOESM2_ESM.pdf]

Cohort study investigating gout flares and management in UK general practice

Codes used in database creation and frequency of occurance in cohort of gout codes

READ_CODE	Description	Contribution to cohort N(%)
C34..00	Gout	86956 (94.85)
N023.00	Gouty arthritis	446 (0.49)
1443	H/O: gout	2174 (2.37)
C34y200	Gouty tophi of other sites	35 (0.04)
C34y500	Gouty tophi of hand	166 (0.18)
C340.00	Gouty arthropathy	164 (0.18)
C342.00	Idiopathic gout	39 (0.04)
N023z00	Gouty arthritis NOS	98 (0.11)
6691	Initial gout assessment	-
669..00	Gout monitoring	811 (0.88)
2D52.00	O/E - auricle of ear - tophi	18 (0.02)
C345.00	Gout due to impairment of renal function	4 (0.00)
C34z.00	Gout NOS	586 (0.64)
C34yz00	Other specified gouty manifestation NOS	2 (0.00)
C34y.00	Other specified gouty manifestation	16 (0.02)
6693	Joints gout affected	-
6695	Date gout treatment started	25 (0.03)
6692	Follow-up gout assessment	11 (0.01)
N023700	Gouty arthritis of the ankle and foot	4 (0.00)
C34y000	Gouty tophi of ear	20 (0.02)
C344.00	Drug-induced gout	1 (0.00)
N023300	Gouty arthritis of the forearm	-
N023600	Gouty arthritis of the lower leg	-
C34y300	Gouty iritis	-
N023400	Gouty arthritis of the hand	1 (0.00)
669Z.00	Gout monitoring NOS	7 (0.00)
C341.00	Gouty nephropathy	-
G557300	Gouty tophi of heart	-
N023x00	Gouty arthritis of multiple sites	4 (0.00)
6697	Gout associated problems	-
C34y400	Gouty neuritis	-
N023y00	Gouty arthritis of other specified site	-
C341z00	Gouty nephropathy NOS	1 (0.00)
6696	Date of last gout attack	-
N023100	Gouty arthritis of the shoulder region	84 (0.09)
N023800	Gouty arthritis of toe	-
C34y100	Gouty tophi of heart	-
Nyu1700	[X]Other secondary gout	-
N023200	Gouty arthritis of the upper arm	-

Oral steroids

DRUG_CODE_	DESCRIPTION
78144	Prednisolone 25mg tablets (Phoenix Healthcare Distribution Ltd) Prednisolone 25mg Tablet Oral
34660	Prednisolone 1mg tablets (Kent Pharmaceuticals Ltd) Prednisolone 1mg Tablet Oral
25272	Precortisyl 1mg Tablet (Hoechst Marion Roussel) Prednisolone 1mg Tablet Oral
41515	Prednisolone 5mg tablets (Teva UK Ltd) Prednisolone 5mg Tablet Oral
73678	Prednisolone 5mg gastro-resistant tablets (DE Pharmaceuticals) Prednisolone 5mg Gastro-resistant tablet Oral
74493	Prednisolone 5mg soluble tablets (Actavis UK Ltd) Prednisolone sodium phosphate 5mg Soluble tablet Oral
75001	Prednisolone 1mg tablets (DE Pharmaceuticals) Prednisolone 1mg Tablet Oral
58234	Prednisolone 10mg/5ml oral solution Prednisolone 2mg/1ml Oral solution Oral
54118	Prednisolone 25mg/5ml oral suspension Prednisolone 5mg/1ml Oral suspension Oral
60421	Prednisolone 5mg tablets (Strides Pharma UK Ltd) Prednisolone 5mg Tablet Oral
64007	Pevanti 10mg tablets (Advanz Pharma) Prednisolone 10mg Tablet Oral
32803	Prednisolone 5mg gastro-resistant tablets (Accord Healthcare Ltd) Prednisolone 5mg Gastro-resistant tablet Oral
34393	Prednisolone 5mg gastro-resistant tablets (Teva UK Ltd) Prednisolone 5mg Gastro-resistant tablet Oral
58987	Prednisolone 5mg gastro-resistant tablets (Phoenix Healthcare Distribution Ltd) Prednisolone 5mg Gastro-resistant tablet Oral
79930	Prednisolone 2.5mg gastro-resistant tablets (Phoenix Healthcare Distribution Ltd) Prednisolone 2.5mg Gastro-resistant tablet Oral
2704	Prednisolone 25mg tablets Prednisolone 25mg Tablet Oral
34631	Prednisolone 1mg Tablet (Co-Pharma Ltd) Prednisolone 1mg Tablet Oral
27959	PREDNISOLONE
27889	PREDNISOLONE
64128	Pevanti 5mg tablets (Advanz Pharma) Prednisolone 5mg Tablet Oral
53336	Prednisolone 25mg tablets (A A H Pharmaceuticals Ltd) Prednisolone 25mg Tablet Oral
59229	Dilacort 5mg gastro-resistant tablets (Auden McKenzie (Pharma Division) Ltd) Prednisolone 5mg Gastro-resistant tablet Oral
63082	Prednisolone 20mg tablets Prednisolone 20mg Tablet Oral
34748	Prednisolone 1mg tablets (Teva UK Ltd) Prednisolone 1mg Tablet Oral
63214	Prednisolone 5mg soluble tablets (Alliance Healthcare (Distribution) Ltd) Prednisolone sodium phosphate 5mg Soluble tablet Oral
73294	Prednisolone 2.5mg/5ml oral solution Prednisolone 500microgram/1ml Oral solution Oral
34914	Prednisolone 1mg Tablet (Celltech Pharma Europe Ltd) Prednisolone 1mg Tablet Oral
34978	Prednisolone 1mg tablets (Wockhardt UK Ltd) Prednisolone 1mg Tablet Oral
2368	Prednisolone 2.5mg tablet Prednisolone 2.5mg Tablets Oral
66645	Prednisolone 5mg/5ml oral solution unit dose (Logixx Pharma Solutions Ltd) Prednisolone 1mg/1ml Oral solution Oral
578	Prednisolone 1mg tablets Prednisolone 1mg Tablet Oral
41745	Prednisolone 25mg tablets (Zentiva) Prednisolone 25mg Tablet Oral
28376	Prednisolone 2.5mg Gastro-resistant tablet (Biorex Laboratories Ltd) Prednisolone 2.5mg Gastro-resistant tablet Oral
34452	Prednisolone 1mg tablets (A A H Pharmaceuticals Ltd) Prednisolone 1mg Tablet Oral
34781	Prednisolone 5mg tablets (Kent Pharmaceuticals Ltd) Prednisolone 5mg Tablet Oral

76020	Prednisolone 2.5mg gastro-resistant tablets (Alliance Healthcare (Distribution) Ltd) Prednisolone 2.5mg Gastro-resistant tablet Oral
80110	Dilacort 2.5mg gastro-resistant tablets (Crescent Pharma Ltd) Prednisolone 2.5mg Gastro-resistant tablet Oral
7584	PREDNISOLONE 4 MG TAB
64009	Pevanti 20mg tablets (Advanz Pharma) Prednisolone 20mg Tablet Oral
5913	Deltacortril 2.5mg gastro-resistant tablets (Alliance Pharmaceuticals Ltd) Prednisolone 2.5mg Gastro-resistant tablet Oral
20095	Precortisyl forte 25mg Tablet (Aventis Pharma) Prednisolone 25mg Tablet Oral
65020	Prednisolone 25mg/5ml oral solution Prednisolone 5mg/1ml Oral solution Oral
44	Prednisolone 5mg gastro-resistant tablets Prednisolone 5mg Gastro-resistant tablet Oral
45302	Prednisolone 5mg Tablet (Biorex Laboratories Ltd) Prednisolone 5mg Tablet Oral
72421	Prednisolone 1mg gastro-resistant tablets (A A H Pharmaceuticals Ltd) Prednisolone 1mg Gastro-resistant tablet Oral
31532	Prednisolone 5mg gastro-resistant tablets (A A H Pharmaceuticals Ltd) Prednisolone 5mg Gastro-resistant tablet Oral
38407	Prednisolone 20mg tablet Prednisolone 20mg Tablets Oral
67076	Prednisolone 20mg/5ml oral solution Prednisolone 4mg/1ml Oral solution Oral
78129	Dilacort 5mg gastro-resistant tablets (Teva UK Ltd) Prednisolone 5mg Gastro-resistant tablet Oral
28859	Deltastab 5mg Tablet (Waymade Healthcare Plc) Prednisolone 5mg Tablet Oral
61689	Prednisolone 5mg soluble tablets (A A H Pharmaceuticals Ltd) Prednisolone sodium phosphate 5mg Soluble tablet Oral
28375	Prednisolone 2.5mg gastro-resistant tablets (A A H Pharmaceuticals Ltd) Prednisolone 2.5mg Gastro-resistant tablet Oral
63791	Prednisolone 5mg/5ml oral solution unit dose Prednisolone 1mg/1ml Oral solution Oral
13522	PREDNISOLONE 2 MG TAB
61162	Prednisolone 5mg tablets (Waymade Healthcare Plc) Prednisolone 5mg Tablet Oral
67107	Prednisolone 5mg gastro-resistant tablets (Alliance Pharmaceuticals Ltd) Prednisolone 5mg Gastro-resistant tablet Oral
47142	Prednisolone 5mg Soluble tablet (Amdipharm Plc) Prednisolone sodium phosphate 5mg Soluble tablet Oral
75763	Prednisolone 5mg Tablet (Celltech Pharma Europe Ltd) Prednisolone 5mg Tablet Oral
73553	Prednisolone 1mg tablets (Alliance Healthcare (Distribution) Ltd) Prednisolone 1mg Tablet Oral
59338	Prednisolone 1mg/5ml oral solution Prednisolone 200microgram/1ml Oral solution Oral
21417	Prednisolone 5mg tablets (A A H Pharmaceuticals Ltd) Prednisolone 5mg Tablet Oral
3059	PREDNISOLONE 50 MG TAB
54434	Prednisolone 2.5mg/5ml oral suspension Prednisolone 500microgram/1ml Oral suspension Oral
53313	Prednisolone 20mg/5ml oral suspension Prednisolone 4mg/1ml Oral suspension Oral
65626	Prednisolone 10mg/5ml oral suspension Prednisolone 2mg/1ml Oral suspension Oral
34404	Prednisolone 1mg tablets (Accord Healthcare Ltd) Prednisolone 1mg Tablet Oral
69568	Dilacort 5mg gastro-resistant tablets (Crescent Pharma Ltd) Prednisolone 5mg Gastro-resistant tablet Oral
19141	Prednisolone 5mg soluble tablets (Advanz Pharma) Prednisolone sodium phosphate 5mg Soluble tablet Oral
59912	Prednisolone 5mg gastro-resistant tablets (Waymade Healthcare Plc) Prednisolone 5mg Gastro-resistant tablet Oral

70603	Prednisolone 5mg soluble tablets (Focus Pharmaceuticals Ltd) Prednisolone sodium phosphate 5mg Soluble tablet Oral
55480	Prednisolone 2.5mg gastro-resistant tablets (Alliance Pharmaceuticals Ltd) Prednisolone 2.5mg Gastro-resistant tablet Oral
5490	Deltacortril 5mg gastro-resistant tablets (Alliance Pharmaceuticals Ltd) Prednisolone 5mg Gastro-resistant tablet Oral
51753	Prednisolone 1mg tablets (Strides Pharma UK Ltd) Prednisolone 1mg Tablet Oral
34109	Prednisolone 5 mg gastro-resistant tablet Prednisolone 5mg Enteric Coated Tablets Oral
66550	Prednisolone 5mg gastro-resistant tablets (Alliance Healthcare (Distribution) Ltd) Prednisolone 5mg Gastro-resistant tablet Oral
58384	Prednisolone 1mg tablets (Almus Pharmaceuticals Ltd) Prednisolone 1mg Tablet Oral
69811	Prednisolone 30mg tablets (Actavis UK Ltd) Prednisolone 30mg Tablet Oral
2799	PREDNISOLONE 10 MG TAB
59283	Dilacort 2.5mg gastro-resistant tablets (Auden McKenzie (Pharma Division) Ltd) Prednisolone 2.5mg Gastro-resistant tablet Oral
9727	Prednisolone 50mg tablets Prednisolone 50mg Tablet Oral
95	Prednisolone 5mg tablets Prednisolone 5mg Tablet Oral
56891	Prednisolone 1mg tablets (Waymade Healthcare Plc) Prednisolone 1mg Tablet Oral
61132	Prednisolone 1mg tablets (Boston Healthcare Ltd) Prednisolone 1mg Tablet Oral
33988	Prednisolone 5mg Tablet (Co-Pharma Ltd) Prednisolone 5mg Tablet Oral
63066	Prednisolone 2.5mg tablets Prednisolone 2.5mg Tablet Oral
32835	Prednisolone 5mg tablets (Wockhardt UK Ltd) Prednisolone 5mg Tablet Oral
67559	Prednisolone 5mg/5ml oral solution unit dose (A A H Pharmaceuticals Ltd) Prednisolone 1mg/1ml Oral solution Oral
955	Prednisolone 5mg soluble tablets Prednisolone sodium phosphate 5mg Soluble tablet Oral
34461	Prednisolone 2.5mg gastro-resistant tablets (Accord Healthcare Ltd) Prednisolone 2.5mg Gastro-resistant tablet Oral
67507	Prednisolone 30mg tablets Prednisolone 30mg Tablet Oral
557	Prednisolone 2.5mg gastro-resistant tablets Prednisolone 2.5mg Gastro-resistant tablet Oral
66914	Prednisolone 1mg gastro-resistant tablets Prednisolone 1mg Gastro-resistant tablet Oral
79091	Prednisolone 1.5mg/5ml oral solution Prednisolone 300microgram/1ml Oral solution Oral
33691	Prednisolone 5mg Gastro-resistant tablet (Biorex Laboratories Ltd) Prednisolone 5mg Gastro-resistant tablet Oral
58000	Prednisolone 5mg tablets (Almus Pharmaceuticals Ltd) Prednisolone 5mg Tablet Oral
55024	Prednisolone 5mg/5ml oral solution Prednisolone 1mg/1ml Oral solution Oral
23512	Precortisyl 5mg Tablet (Hoechst Marion Roussel) Prednisolone 5mg Tablet Oral
69686	Pevanti 25mg tablets (Advanz Pharma) Prednisolone 25mg Tablet Oral
78789	Prednisolone 5mg soluble tablets (DE Pharmaceuticals) Prednisolone sodium phosphate 5mg Soluble tablet Oral
63549	Prednisolone 1mg/ml oral solution (Logixx Pharma Solutions Ltd) Prednisolone 1mg/1ml Oral solution Oral
68497	Prednisolone 2.5mg gastro-resistant tablets (Waymade Healthcare Plc) Prednisolone 2.5mg Gastro-resistant tablet Oral
58369	Prednisolone 5mg tablets (Boston Healthcare Ltd) Prednisolone 5mg Tablet Oral
63172	Prednisolone 10mg tablets Prednisolone 10mg Tablet Oral
29333	Prednisolone 5mg tablets (Actavis UK Ltd) Prednisolone 5mg Tablet Oral

80050	Prednisolone 1mg gastro-resistant tablets (Alliance Healthcare (Distribution) Ltd) Prednisolone 1mg Gastro-resistant tablet Oral
7710	PREDNISOLONE 15 MG TAB
64008	Pevanti 2.5mg tablets (Advanz Pharma) Prednisolone 2.5mg Tablet Oral
74239	Prednisolone 15mg/5ml oral solution Prednisolone 3mg/1ml Oral solution Oral
33990	Prednisolone 5mg Tablet (IVAX Pharmaceuticals UK Ltd) Prednisolone 5mg Tablet Oral
64416	Prednisolone 10mg/ml oral solution sugar free Prednisolone 10mg/1ml Oral solution Oral
77760	Prednisolone 5mg soluble tablets (Phoenix Labs Ltd) Prednisolone sodium phosphate 5mg Soluble tablet Oral
64221	Prednisolone 5mg/5ml oral suspension Prednisolone 1mg/1ml Oral suspension Oral
27962	Deltastab 1mg Tablet (Waymade Healthcare Plc) Prednisolone 1mg Tablet Oral
81168	Prednisolone 1mg gastro-resistant tablets (Alliance Pharmaceuticals Ltd)
81344	Prednisolone 2.5mg gastro-resistant tablets (Mawdsley-Brooks & Company Ltd)Prednisolone2.5mgGastro-resistant tabletOral
81369	Prednisolone 5mg tablets (Genesis Pharmaceuticals Ltd)Prednisolone5mgTabletOral
82367	Prednisolone 5mg tablets (Phoenix Healthcare Distribution Ltd)Prednisolone5mgTabletOral
82432	Prednisolone 2.5mg gastro-resistant tablets (Almus Pharmaceuticals Ltd)Prednisolone2.5mgGastro-resistant tabletOral
82675	Prednisolone 10mg/ml oral solution sugar free (A A H Pharmaceuticals Ltd)Prednisolone10mg/1mlOral solutionOral
82743	Prednisolone 1.5mg/5ml oral suspensionPrednisolone300microgram/1mlOral suspensionOral

Intra-articular injection

READ_COI	DESCRIPTION
7K6Z700	Injection of steroid into knee joint
7K6Z500	Injection of steroid into shoulder joint
7L19100	Injection of steroid for local action NEC
7K6Z600	Injection of steroid into ankle joint
7G2A100	Injection of steroid into subcutaneous tissue
7K6ZF00	Injection of steroid into wrist joint
7L19F00	Injection of steroid NEC
7K6ZG00	Injection of steroid into carpometacarpal joint of thumb
7K6ZH00	Injection of steroid into carpometacarpal joint of finger
7K6ZJ00	Injection of steroid into hip joint
7K6ZM00	Injection of steroid into elbow joint

Joint aspiration

READ_CODE	DESCRIPTION
7K6Z800	Aspiration of fluid from knee joint
7K6U400	Aspiration of joint NEC
816y.00	Other therapeutic aspiration

Oral NSAIDs

DRUG_CODE_	DESCRIPTION
33669	Diclofenac 50mg Gastro-resistant tablet (Genus Pharmaceuticals Ltd) Diclofenac sodium 50mg Gastro-resistant tablet Oral
33801	Opustan 250mg Capsule (Opus Pharmaceuticals Ltd) Mefenamic acid 250mg Capsule Oral
3182	Froben 50mg tablets (Abbott Laboratories Ltd) Flurbiprofen 50mg Tablet Oral
14678	Defanac sr 100mg Modified-release tablet (Ranbaxy (UK) Ltd) Diclofenac sodium 100mg Modified-release tablet Oral
50602	Diclofenac potassium 50mg tablets (Alliance Healthcare (Distribution) Ltd) Diclofenac potassium 50mg Tablet Oral
40401	Naproxen 250mg gastro-resistant tablets (IVAX Pharmaceuticals UK Ltd) Naproxen 250mg Gastro-resistant tablet Oral
29037	Valdic 100 Retard tablets (Fannin UK Ltd) Diclofenac sodium 100mg Modified-release tablet Oral
2387	Arthrotec 75 gastro-resistant tablets (Pfizer Ltd) Diclofenac sodium/Misoprostol 75mg + 200microgram Gastro-resistant tablet Oral
34850	Ibuprofen 600mg tablets (Teva UK Ltd) Ibuprofen 600mg Tablet Oral
13818	Nabumetone 500mg tablets (Accord Healthcare Ltd) Nabumetone 500mg Tablet Oral
30164	Lemsip Cold and Flu Sinus 12 Hr Ibuprofen + Pseudoephedrine modified-release capsules (Reckitt Benckiser Healthcare (UK) Ltd) Pseudoephedrine hydrochloride/Ibuprofen 45mg + 300mg Modified-release capsule Oral
46967	Mefenamic acid 250mg Capsule (Sandoz Ltd) Mefenamic acid 250mg Capsule Oral
17532	Dicloflex Retard 100mg tablets (Kent Pharmaceuticals Ltd) Diclofenac sodium 100mg Modified-release tablet Oral
35653	Etopan XL 600mg tablets (Sun Pharmaceutical Industries Europe B.V.) Etodolac 600mg Modified-release tablet Oral
78779	Ketonal 50mg Capsule (Lagap) Ketoprofen 50mg Capsule Oral
46848	Naproxen 500mg Gastro-resistant tablet (Almus Pharmaceuticals Ltd) Naproxen 500mg Gastro-resistant tablet Oral
53700	Naproxen 250mg gastro-resistant tablets (Alliance Healthcare (Distribution) Ltd) Naproxen 250mg Gastro-resistant tablet Oral
3326	Oruvail 100mg Modified-release capsule (Hawgreen Ltd) Ketoprofen 100mg Modified-release capsule Oral
35935	Meloxicam 7.5mg tablets (Somex Pharma) Meloxicam 7.5mg Tablet Oral
57006	Diclofenac sodium 25mg gastro-resistant tablets (Phoenix Healthcare Distribution Ltd) Diclofenac sodium 25mg Gastro-resistant tablet Oral
51293	Diclofenac potassium 50mg tablets (Phoenix Healthcare Distribution Ltd) Diclofenac potassium 50mg Tablet Oral
42108	Ibuprofen 200mg tablets (OBG Pharmaceuticals Ltd) Ibuprofen 200mg Tablet Oral
78172	Naproxen 250mg tablets (IVAX Pharmaceuticals UK Ltd) Naproxen 250mg Tablet Oral
18527	Mandafen 400mg tablets (M & A Pharmachem Ltd) Ibuprofen 400mg Tablet Oral
612	Dicloflex 25mg gastro-resistant tablets (Dexcel-Pharma Ltd) Diclofenac sodium 25mg Gastro-resistant tablet Oral

71989 Meloxicam 15mg tablets (Almus Pharmaceuticals Ltd) Meloxicam 15mg Tablet Oral
Diclofenac potassium 50mg tablets (A A H Pharmaceuticals Ltd) Diclofenac potassium
48059 50mg Tablet Oral
736 Indometacin 50mg capsules Indometacin 50mg Capsule Oral
Voltarol 50mg dispersible tablets (DE Pharmaceuticals) Diclofenac sodium 50mg
50058 Dispersible tablet Oral
1073 Mefenamic acid 500mg tablets Mefenamic acid 500mg Tablet Oral
63612 Suprol 200mg Capsule (Lorex Synthelabo Ltd) 200mg Capsule
20016 Tolmetin 400mg Capsule Tolmetin Sodium 400mg Capsule Oral
12075 Mobiflex 20mg Tablet (Roche Products Ltd) Tenoxicam 20mg Tablet Oral
Diclofenac 50mg dispersible tablets sugar free (DE Pharmaceuticals) Diclofenac
57162 sodium 50mg Dispersible tablet Oral
66544 Ibuprofen 400mg tablets (DE Pharmaceuticals) Ibuprofen 400mg Tablet Oral
34527 Ibuprofen 200mg tablets (Zentiva) Ibuprofen 200mg Tablet Oral
Nurofen Express 512mg tablets (Reckitt Benckiser Healthcare (UK) Ltd) Ibuprofen
44483 sodium dihydrate 400mg Tablet Oral
Naproxen 250mg effervescent tablets sugar free Naproxen 250mg Effervescent
65862 tablet Oral
Ketoprofen 200mg modified-release capsules (A A H Pharmaceuticals Ltd)
77293 Ketoprofen 200mg Modified-release capsule Oral
Timpron 250mg Gastro-resistant tablet (Berk Pharmaceuticals Ltd) Naproxen 250mg
31429 Gastro-resistant tablet Oral
Rheuflex 500mg Tablet (Goldshield Pharmaceuticals Ltd) Naproxen 500mg Tablet
28816 Oral

53345 Voltarol Rapid 50mg tablets (Lexon (UK) Ltd) Diclofenac potassium 50mg Tablet Oral
Naproxen 250mg gastro-resistant tablets (Teva UK Ltd) Naproxen 250mg Gastro-
34290 resistant tablet Oral
Nurofen Maximum Strength Migraine Pain 684mg caplets (Reckitt Benckiser
33935 Healthcare (UK) Ltd) Ibuprofen lysine 400mg Tablet Oral
Diclofenac sodium 50mg gastro-resistant tablets (Kent Pharmaceuticals Ltd)
27055 Diclofenac sodium 50mg Gastro-resistant tablet Oral
Indometacin 50mg Capsule (Meridian Healthcare (UK) Ltd) Indometacin 50mg
36577 Capsule Oral

21815 Arthrofen 600 tablets (Ashbourne Pharmaceuticals Ltd) Ibuprofen 600mg Tablet Oral
Diclofenac sodium 100mg modified-release tablets (Phoenix Healthcare Distribution
67220 Ltd) Diclofenac sodium 100mg Modified-release tablet Oral
5175 Celebrex 100mg capsules (Upjohn UK Ltd) Celecoxib 100mg Capsule Oral
Jomethid XL 200mg capsules (Actavis UK Ltd) Ketoprofen 200mg Modified-release
30327 capsule Oral
Ketoprofen 200mg Modified-release capsule (Actavis UK Ltd) Ketoprofen 200mg
33568 Modified-release capsule Oral
78096 Etoricoxib 60mg tablets (Torrent Pharma (UK) Ltd) Etoricoxib 60mg Tablet Oral
Voltarol Rapid 50mg tablets (Novartis Pharmaceuticals UK Ltd) Diclofenac potassium
5085 50mg Tablet Oral
360 Brufen 100mg/5ml syrup (Mylan) Ibuprofen 20mg/1ml Oral suspension Oral
Brufen 600mg effervescent granules sachets (Necessity Supplies Ltd) Ibuprofen
55009 600mg Effervescent granules Oral

7481	Lederfen 450mg Tablet (Wyeth Pharmaceuticals) Fenbufen 450mg Tablet Oral
74451	Diclofenac sodium 25mg gastro-resistant tablets (Waymade Healthcare Plc) Diclofenac sodium 25mg Gastro-resistant tablet Oral
72925	Celecoxib 100mg capsules (A A H Pharmaceuticals Ltd) Celecoxib 100mg Capsule Oral
42793	Diclofenac 100mg Modified-release tablet (IVAX Pharmaceuticals UK Ltd) Diclofenac sodium 100mg Modified-release tablet Oral
2366	Flurbiprofen 100mg tablets Flurbiprofen 100mg Tablet Oral
34595	Mefenamic acid 500mg tablets (Zentiva) Mefenamic acid 500mg Tablet Oral
42604	Mobiflex 20mg tablets (Mylan) Tenoxicam 20mg Tablet Oral
28172	Ibuprofen 300mg / Pseudoephedrine 45mg modified-release capsules Pseudoephedrine hydrochloride/Ibuprofen 45mg + 300mg Modified-release capsule Oral
526	Aceclofenac 100mg tablets Aceclofenac 100mg Tablet Oral
51614	Ibuprofen 200mg caplets (Lloyds Pharmacy Ltd) Ibuprofen 200mg Tablet Oral
67117	Stirlescent 250mg effervescent tablets (Stirling Anglian Pharmaceuticals Ltd) Naproxen 250mg Effervescent tablet Oral
64595	Misofen 50mg/200microgram gastro-resistant tablets (Morningside Healthcare Ltd) Diclofenac sodium/Misoprostol 50mg + 200microgram Gastro-resistant tablet Oral
16286	Lofensaid Retard 75 tablets (Opus Pharmaceuticals Ltd) Diclofenac sodium 75mg Modified-release tablet Oral
66323	Ebretin 300mg capsules (Ranbaxy (UK) Ltd) Etodolac 300mg Capsule Oral
11554	Ibuprofen 200mg / Codeine 12.8mg tablets Ibuprofen/Codeine phosphate 200mg + 12.8mg Tablet Oral
1866	Naprosyn 500mg tablets (Atnahs Pharma UK Ltd) Naproxen 500mg Tablet Oral
21811	Lidifen 200mg Tablet (Berk Pharmaceuticals Ltd) Ibuprofen 200mg Tablet Oral
31469	Apsifen -f 600mg Tablet (Approved Prescription Services Ltd) Ibuprofen 600mg Tablet Oral
17818	Ketovail 100mg modified-release capsules (Teva UK Ltd) Ketoprofen 100mg Modified-release capsule Oral
40141	Ketoprofen 100mg capsules (A A H Pharmaceuticals Ltd) Ketoprofen 100mg Capsule Oral
20059	Tiaprofenic acid 300mg sachets Tiaprofenic Acid 300mg Sachets Oral
57007	Mefenamic acid 250mg capsules (Essential Generics Ltd) Mefenamic acid 250mg Capsule Oral
34143	Naprosyn 375 Tablet (Roche Products Ltd) Naproxen Tablet Oral
73131	Diclofenac 25mg Gastro-resistant tablet (Almus Pharmaceuticals Ltd) Diclofenac sodium 25mg Gastro-resistant tablet Oral
10785	Fenbid 300mg Spansules (Mercury Pharma Group Ltd) Ibuprofen 300mg Modified-release capsule Oral
32090	Mefenamic acid 500mg tablets (Actavis UK Ltd) Mefenamic acid 500mg Tablet Oral
57297	Mefenamic acid 500mg tablets (Alliance Healthcare (Distribution) Ltd) Mefenamic acid 500mg Tablet Oral
34536	Ibuprofen 400mg tablets (IVAX Pharmaceuticals UK Ltd) Ibuprofen 400mg Tablet Oral
24469	Cuprofen for Children 100mg/5ml oral suspension (SSL International Plc) Ibuprofen 20mg/1ml Oral suspension Oral

26234	Flamatrol 10mg Capsule (Berk Pharmaceuticals Ltd) Piroxicam 10mg Capsule Oral
12709	Ibuprofen and codeine 200mg + 12.5mg Tablet Codeine Phosphate/Ibuprofen 200mg + 12.5mg Tablet Oral
14084	Diclovol 75mg SR tablets (Arun Pharmaceuticals Ltd) Diclofenac sodium 75mg Modified-release tablet Oral
3496	Nycopren 250mg gastro-resistant tablets (Ardern Healthcare Ltd) Naproxen 250mg Gastro-resistant tablet Oral
56441	Calprofen 100mg/5ml oral suspension 5ml sachets (McNeil Products Ltd) Ibuprofen 20mg/1ml Oral suspension Oral
68018	Ibuprofen 200mg tablets (Mawdsley-Brooks & Company Ltd) Ibuprofen 200mg Tablet Oral
21821	Lidifen f 600mg Tablet (Berk Pharmaceuticals Ltd) Ibuprofen 600mg Tablet Oral
73040	Nurofen for Children 100mg chewable capsules (Reckitt Benckiser Healthcare (UK) Ltd) Ibuprofen 100mg Chewable capsule Oral
32227	Larafen CR 200mg capsules (Ennogen Pharma Ltd) Ketoprofen 200mg Modified-release capsule Oral
54137	Ibuprofen 400mg tablets (Aspar Pharmaceuticals Ltd) Ibuprofen 400mg Tablet Oral
59880	Diclofenac sodium 75mg modified-release capsules (Waymade Healthcare Plc) Diclofenac sodium 75mg Modified-release capsule Oral
66567	Ibuprofen sodium dihydrate 400mg tablets Ibuprofen sodium dihydrate 400mg Tablet Oral
5254	Celecoxib 200mg capsules Celecoxib 200mg Capsule Oral
32105	Mefenamic acid 500mg tablets (A A H Pharmaceuticals Ltd) Mefenamic acid 500mg Tablet Oral
27438	Pseudoephedrine 45mg with ibuprofen 300mg modified-release capsule Ibuprofen/Pseudoephedrine Hydrochloride 45mg + 300mg Modified Release Capsules Oral
34898	Mefenamic acid 250mg Capsule (Berk Pharmaceuticals Ltd) Mefenamic acid 250mg Capsule Oral
43032	Inoven 200mg Tablet (Janssen-Cilag Ltd) Ibuprofen 200mg Tablet Oral
54660	Diclofenac sodium 50mg capsules Diclofenac Sodium
13459	Dysman 500 tablets (Ashbourne Pharmaceuticals Ltd) Mefenamic acid 500mg Tablet Oral
14333	Ibuprofen 400mg capsules Ibuprofen 400mg Capsule Oral
21045	Ibumetin 400mg Tablet (Alfred Benzon (UK) Ltd) Ibuprofen 400mg Tablet Oral
13347	Alrheumat 50mg Capsule (Bayer Plc) Ketoprofen 50mg Capsule Oral
928	Diclofenac sodium 25mg tablets Diclofenac Sodium 25mg Tablets Oral
57370	Meloxicam 15mg orodispersible tablets sugar free Meloxicam 15mg Orodispersible tablet Oral
18820	Fenpaed 100mg/5ml Oral suspension (Pinewood Healthcare) Ibuprofen 100mg/5ml Oral Suspension Oral
70393	Naproxen 250mg tablets (DE Pharmaceuticals) Naproxen 250mg Tablet Oral
53576	Arcoxia 120mg tablets (DE Pharmaceuticals) Etoricoxib 120mg Tablet Oral
36260	Mendys 250mg Capsule (Kent Pharmaceuticals Ltd) Mefenamic acid 250mg Capsule Oral
18812	Nurofen meltlets lemon 200mg Orodispersible tablet (Reckitt Benckiser Healthcare (UK) Ltd) Ibuprofen 200mg Orodispersible tablet Oral

Voltarol Pain-eze Extra Strength 25mg tablets (Novartis Consumer Health UK Ltd)
47820 Diclofenac potassium 25mg Tablet Oral
Axorid 200mg/20mg modified-release capsules (Meda Pharmaceuticals Ltd)
41365 Omeprazole/Ketoprofen 20mg + 200mg Modified-release capsule Oral
34961 Ibuprofen 600mg tablets (Sandoz Ltd) Ibuprofen 600mg Tablet Oral
43541 Piroxicam 10mg capsules (Actavis UK Ltd) Piroxicam 10mg Capsule Oral
Timpron 500mg Gastro-resistant tablet (Berk Pharmaceuticals Ltd) Naproxen 500mg
26231 Gastro-resistant tablet Oral
13380 Clinoril 200mg tablets (Merck Sharp & Dohme Ltd) Sulindac 200mg Tablet Oral
55582 Celebrex 200mg capsules (Lexon (UK) Ltd) Celecoxib 200mg Capsule Oral
14385 Cuprofen 200mg Tablet (SSL International Plc) Ibuprofen 200mg Tablet Oral
Berlind 75 Retard capsules (Tillomed Laboratories Ltd) Indometacin 75mg Modified-
77102 release capsule Oral
Lofensaid 50mg gastro-resistant tablets (Opus Pharmaceuticals Ltd) Diclofenac
18798 sodium 50mg Gastro-resistant tablet Oral
Ibuprofen with pseudoephedrine hc 400mg + 60mg Liquid
28822 Ibuprofen/Pseudoephedrine Hydrochloride 400mg + 60mg Liquid Oral

71027 Piroxicam 10mg capsules (Sigma Pharmaceuticals Plc) Piroxicam 10mg Capsule Oral
9822 Arcoxia 120mg tablets (Merck Sharp & Dohme Ltd) Etoricoxib 120mg Tablet Oral
Piroxicam 20mg capsules (IVAX Pharmaceuticals UK Ltd) Piroxicam 20mg Capsule
41623 Oral
Diclofenac 10mg/5ml oral suspension Diclofenac sodium 2mg/1ml Oral suspension
61762 Oral
Dicloflex 50mg Gastro-resistant tablet (Ratiopharm UK Ltd) Diclofenac sodium 50mg
9886 Gastro-resistant tablet Oral
First Resort Double Action Pain Relief 12.5mg tablets (Actavis UK Ltd) Diclofenac
45814 potassium 12.5mg Tablet Oral
Ketoprofen 200mg modified-release capsules Ketoprofen 200mg Modified-release
3043 capsule Oral
Pranoxen continus 375mg Tablet (Napp Pharmaceuticals Ltd) Naproxen 375mg
21843 Tablet Oral
15159 Tolfenamic acid 200mg Capsule Tolfenamic Acid 200mg Capsule Oral
Dicloflex sr 75mg Tablet (Ratiopharm UK Ltd) Diclofenac sodium 75mg Modified-
17491 release tablet Oral
Ibuprofen 100mg/5ml oral suspension 5ml sachets sugar free Ibuprofen 20mg/1ml
48562 Oral suspension Oral
Valenac ec 50mg Gastro-resistant tablet (Shire Pharmaceuticals Ltd) Diclofenac
25283 sodium 50mg Gastro-resistant tablet Oral
Nurofen 200mg tablets (Reckitt Benckiser Healthcare (UK) Ltd) Ibuprofen 200mg
36650 Tablet Oral
Nurofen Express 684mg caplets (Reckitt Benckiser Healthcare (UK) Ltd) Ibuprofen
36787 lysine 400mg Tablet Oral

78149 Ibuprofen 600mg Tablet (IVAX Pharmaceuticals UK Ltd) Ibuprofen 600mg Tablet Oral

56762 Naproxen 100mg/5ml oral suspension Naproxen 20mg/1ml Oral suspension Oral
60772 Indometacin 25mg/5ml oral solution Indometacin 5mg/1ml Oral solution Oral
33321 Indometacin 50mg capsules (Actavis UK Ltd) Indometacin 50mg Capsule Oral
10169 Brexidol 20mg tablets (Chiesi Ltd) Piroxicam betadex 20mg Tablet Oral

Naprosyn EC 250mg tablets (Atnahs Pharma UK Ltd) Naproxen 250mg Gastro-
3972 resistant tablet Oral
77260 Meloxicam 15mg tablets (Teva UK Ltd) Meloxicam 15mg Tablet Oral
Tolectin 200mg Capsule (Cilag Pharmaceuticals Ltd) Tolmetin Sodium 200mg Capsule
18640 Oral
29704 Paxofen 200mg Tablet (M A Steinhard Ltd) Ibuprofen 200mg Tablet Oral
40484 Orudis 100mg capsules (Sanofi) Ketoprofen 100mg Capsule Oral
Diclofenac sodium 75mg modified-release capsules (Waymade Healthcare Plc)
71064 Diclofenac sodium 75mg Modified-release capsule Oral
Arthrotec 50 gastro-resistant tablets (Pfizer Ltd) Diclofenac sodium/Misoprostol
162 50mg + 200microgram Gastro-resistant tablet Oral
Slofenac 75mg SR tablets (Sterwin Medicines) Diclofenac sodium 75mg Modified-
19382 release tablet Oral
Ketoprofen 100mg / Omeprazole 20mg modified-release capsules
41364 Ketoprofen/Omeprazole 100mg + 20mg Modified-release capsule Oral
Ibuprofen 100mg/5ml oral suspension sugar free (DE Pharmaceuticals) Ibuprofen
73112 20mg/1ml Oral suspension Oral

78343 Naproxen 500mg tablets (Noumed Life Sciences Ltd) Naproxen 500mg Tablet Oral
1086 Ibuprofen 600mg tablets Ibuprofen 600mg Tablet Oral
Indocid R 75mg capsules (Merck Sharp & Dohme Ltd) Indometacin 75mg Modified-
1496 release capsule Oral
29465 Piroxicam 20mg capsules (Actavis UK Ltd) Piroxicam 20mg Capsule Oral
Tolectin 400mg Capsule (Cilag Pharmaceuticals Ltd) Tolmetin Sodium 400mg Capsule
10711 Oral
43904 Feminax Express 342mg tablets (Bayer Plc) Ibuprofen lysine 200mg Tablet Oral
Ketoprofen 200mg Modified-release capsule (Generics (UK) Ltd) Ketoprofen 200mg
46920 Modified-release capsule Oral
661 Naproxen 250mg tablets Naproxen 250mg Tablet Oral

56039 Ibuprofen 600mg tablets (Waymade Healthcare Plc) Ibuprofen 600mg Tablet Oral
Fenactol SR 75mg tablets (Discovery Pharmaceuticals) Diclofenac sodium 75mg
17126 Modified-release tablet Oral
Pardelprin MR 75mg capsules (Actavis UK Ltd) Indometacin 75mg Modified-release
23204 capsule Oral
Feminax Ultra 250mg gastro-resistant tablets (Bayer Plc) Naproxen 250mg Gastro-
38511 resistant tablet Oral
Acoflam 75mg SR tablets (Mercury Pharma Group Ltd) Diclofenac sodium 75mg
75442 Modified-release tablet Oral

24193 Imbrilon 25mg Capsule (Berk Pharmaceuticals Ltd) Indometacin 25mg Capsule Oral
Nurofen Express 400mg liquid capsules (Reckitt Benckiser Healthcare (UK) Ltd)
37648 Ibuprofen 400mg Capsule Oral
Volsaid Retard 75 tablets (Chiesi Ltd) Diclofenac sodium 75mg Modified-release
4506 tablet Oral
Diclofenac sodium 25mg gastro-resistant tablets (Sterwin Medicines) Diclofenac
62636 sodium 25mg Gastro-resistant tablet Oral
78072 Etoricoxib 120mg tablets (Accord Healthcare Ltd) Etoricoxib 120mg Tablet Oral
Naproxen 250mg gastro-resistant tablets (Ranbaxy (UK) Ltd) Naproxen 250mg Gastro-
70698 resistant tablet Oral

52338	Diclofenac potassium 50mg tablets (Focus Pharmaceuticals Ltd) Diclofenac potassium 50mg Tablet Oral
65025	Ibuprofen 400mg tablets (Crescent Pharma Ltd) Ibuprofen 400mg Tablet Oral
43456	Anadin LiquiFast 400mg capsules (Pfizer Consumer Healthcare Ltd) Ibuprofen 400mg Capsule Oral
34744	Diclofenac 100mg Modified-release capsule (Sandoz Ltd) Diclofenac sodium 100mg Modified-release capsule Oral
40215	Oruvail 100 modified-release capsules (Sanofi) Ketoprofen 100mg Modified-release capsule Oral
31945	Naproxen 500mg Gastro-resistant tablet (Sterwin Medicines) Naproxen 500mg Gastro-resistant tablet Oral
31944	Diclofenac sodium 25mg gastro-resistant tablets (Mylan) Diclofenac sodium 25mg Gastro-resistant tablet Oral
76041	Ibular 400mg tablets (Ennogen Pharma Ltd) Ibuprofen 400mg Tablet Oral
16001	Ibuprofen 200mg tablets (A A H Pharmaceuticals Ltd) Ibuprofen 200mg Tablet Oral
24137	Indometacin 25mg capsules (Actavis UK Ltd) Indometacin 25mg Capsule Oral
69477	Voltarol Rapid 50mg tablets (DE Pharmaceuticals) Diclofenac potassium 50mg Tablet Oral
37816	Cuprofen PLUS tablets (SSL International Plc) Ibuprofen/Codeine phosphate 200mg + 12.8mg Tablet Oral
56898	Rhumalgan SR 75mg capsules (Actavis UK Ltd) Diclofenac sodium 75mg Modified-release capsule Oral
46656	Chloraprep with tint solution (CareFusion U.K. Ltd) Isopropyl Alcohol/Chlorhexidine Gluconate Oral Solution Topical
79433	Indometacin 25mg capsules (Waymade Healthcare Plc) Indometacin 25mg Capsule Oral
60443	Diclofenac sodium 75mg modified-release capsules (DE Pharmaceuticals) Diclofenac sodium 75mg Modified-release capsule Oral
40756	Dicloflex 25mg gastro-resistant tablets (Almus Pharmaceuticals Ltd) Diclofenac sodium 25mg Gastro-resistant tablet Oral
12122	Orudis 50mg Capsule (Hawgreen Ltd) Ketoprofen 50mg Capsule Oral
259	Mefenamic acid 250mg capsules Mefenamic acid 250mg Capsule Oral
51827	Mefenamic acid 500mg tablets (Sigma Pharmaceuticals Plc) Mefenamic acid 500mg Tablet Oral
47501	Rhumalgan SR 75mg capsules (Almus Pharmaceuticals Ltd) Diclofenac sodium 75mg Modified-release capsule Oral
26165	Diclofenac sodium 50mg gastro-resistant tablets (A A H Pharmaceuticals Ltd) Diclofenac sodium 50mg Gastro-resistant tablet Oral
389	Ketoprofen 50mg capsules Ketoprofen 50mg Capsule Oral
58048	Diclofenac sodium 50mg gastro-resistant tablets (Waymade Healthcare Plc) Diclofenac sodium 50mg Gastro-resistant tablet Oral
474	Celecoxib 100mg capsules Celecoxib 100mg Capsule Oral
74952	Arcoxia 60mg tablets (Waymade Healthcare Plc) Etoricoxib 60mg Tablet Oral
1755	Piroxicam 20mg capsules Piroxicam 20mg Capsule Oral
8969	Lodine 300mg Capsule (Shire Pharmaceuticals Ltd) Etodolac 300mg Capsule Oral
76608	Indotard 75mg Modified-release capsule (Galen Ltd) Indometacin 75mg Modified-release capsule Oral
68685	Naproxen 250mg tablets (Waymade Healthcare Plc) Naproxen 250mg Tablet Oral

34769	Naproxen 500mg tablets (A A H Pharmaceuticals Ltd) Naproxen 500mg Tablet Oral
71362	Diclomax Retard 100mg capsules (Waymade Healthcare Plc) Diclofenac sodium 100mg Modified-release capsule Oral
48218	Dicloflex sr 100mg Tablet (Teva UK Ltd) Diclofenac sodium 100mg Modified-release tablet Oral
4216	Brufen 600mg tablets (Mylan) Ibuprofen 600mg Tablet Oral
26216	Timpron 500mg Tablet (Berk Pharmaceuticals Ltd) Naproxen 500mg Tablet Oral
55153	Nurofen Express Soluble 400mg oral powder sachets (Reckitt Benckiser Healthcare (UK) Ltd) Ibuprofen lysine 400mg Powder Oral
26970	Ibuprofen 100mg/5ml oral suspension sugar free (Teva UK Ltd) Ibuprofen 20mg/1ml Oral suspension Oral
70878	Nurofen Joint & Back Pain Relief 200mg capsules (Reckitt Benckiser Healthcare (UK) Ltd) Ibuprofen 200mg Capsule Oral
75765	Indometacin 50mg Capsule (Celltech Pharma Europe Ltd) Indometacin 50mg Capsule Oral
52617	Ibuprofen 100mg/5ml oral suspension sugar free (Sigma Pharmaceuticals Plc) Ibuprofen 20mg/1ml Oral suspension Oral
34743	Naproxen 500mg gastro-resistant tablets (A A H Pharmaceuticals Ltd) Naproxen 500mg Gastro-resistant tablet Oral
33645	Diclofenac 75mg Modified-release tablet (IVAX Pharmaceuticals UK Ltd) Diclofenac sodium 75mg Modified-release tablet Oral
1984	Diclofenac sodium 100mg modified-release tablets Diclofenac Sodium 100mg Modified Release Tablet Oral
49266	Ibuprofen for Children 100mg/5ml oral suspension (Galpharm International Ltd) Ibuprofen 20mg/1ml Oral suspension Oral
31383	Dexomon 75mg SR tablets (Hillcross Pharmaceuticals Ltd) Diclofenac sodium 75mg Modified-release tablet Oral
3421	Diclomax sr 75mg Modified-release capsule (Provalis Healthcare Ltd) Diclofenac sodium 75mg Modified-release capsule Oral
44313	Indoflex 25mg Capsule (Unimed Pharmaceuticals Ltd) Indometacin 25mg Capsule Oral
67608	Piroxicam 20mg dispersible tablets (A A H Pharmaceuticals Ltd) Piroxicam 20mg Dispersible tablet Oral
39823	Dicloflex 50mg gastro-resistant tablets (Almus Pharmaceuticals Ltd) Diclofenac sodium 50mg Gastro-resistant tablet Oral
33559	Diclofenac 50mg Tablet (C P Pharmaceuticals Ltd) Diclofenac sodium 50mg Gastro-resistant tablet Oral
74028	Diclomax SR 75mg capsules (Waymade Healthcare Plc) Diclofenac sodium 75mg Modified-release capsule Oral
59562	Ibuprofen 600mg tablets (Boston Healthcare Ltd) Ibuprofen 600mg Tablet Oral
41367	Ketoprofen 200mg / Omeprazole 20mg modified-release capsules Omeprazole/Ketoprofen 20mg + 200mg Modified-release capsule Oral
4911	Ibuprofen 400mg Granules Ibuprofen 400mg Granules Oral
34362	Diclofenac 25mg Gastro-resistant tablet (Genus Pharmaceuticals Ltd) Diclofenac sodium 25mg Gastro-resistant tablet Oral
18662	Indomod 75mg modified-release capsules (Pfizer Ltd) Indometacin 75mg Modified-release capsule Oral
74005	Oruvail 200 modified-release capsules (Dowelhurst Ltd) Ketoprofen 200mg Modified-release capsule Oral

3710	Piroxicam 20mg dispersible tablets Piroxicam 20mg Dispersible tablet Oral
76583	Berlind 75mg Modified-release capsule (Neo Laboratories Ltd) Indometacin 75mg Modified-release capsule Oral
10149	Ibuprofen 200mg capsules Ibuprofen 200mg Capsule Oral
50343	Feldene 0.5% gel (DE Pharmaceuticals) Piroxicam 5mg/1gram Gel Cutaneous
33180	Ketoprofen cr 200mg Capsule (Bristol-Myers Squibb Pharmaceuticals Ltd) Ketoprofen 200mg Modified-release capsule Oral
20384	Flamatak MR 100mg tablets (Actavis UK Ltd) Diclofenac sodium 100mg Modified-release tablet Oral
31950	Diclofenac sodium 50mg gastro-resistant tablets (Sterwin Medicines) Diclofenac sodium 50mg Gastro-resistant tablet Oral
54783	Naproxen 250mg tablets (Teva UK Ltd) Naproxen 250mg Tablet Oral
3409	Feldene 20mg Orodispersible tablet (Pfizer Ltd) Piroxicam 20mg Orodispersible tablet Oral
71104	Oruvail 200 modified-release capsules (Waymade Healthcare Plc) Ketoprofen 200mg Modified-release capsule Oral
15180	Naproxen and misoprostol 500mgwith200microgram combined Tablet Naproxen/Misoprostol 500mg+200mcg Tablet Oral
28479	Nurofen Back Pain SR 300mg capsules (Reckitt Benckiser Healthcare (UK) Ltd) Ibuprofen 300mg Modified-release capsule Oral
31787	Econac SR 75mg tablets (Advanz Pharma) Diclofenac sodium 75mg Modified-release tablet Oral
55486	Naproxen 500mg tablets (Teva UK Ltd) Naproxen 500mg Tablet Oral
1446	Voltarol 50mg Tablet (Novartis Pharmaceuticals UK Ltd) Diclofenac sodium 50mg Gastro-resistant tablet Oral
3597	Nurofen 200mg Soluble tablet (Crookes Healthcare Ltd) Ibuprofen 200mg Soluble Tablet Oral
40185	Oruvail 200 modified-release capsules (Sanofi) Ketoprofen 200mg Modified-release capsule Oral
79731	Diclofenac sodium 25mg gastro-resistant tablets (Medreich Plc) Diclofenac sodium 25mg Gastro-resistant tablet Oral
22283	Lemsip flu 12 hr Modified-release capsule (Reckitt Benckiser Healthcare (UK) Ltd) Pseudoephedrine hydrochloride/Ibuprofen 45mg + 300mg Modified-release capsule Oral
784	Ibuprofen 300mg modified-release capsules Ibuprofen 300mg Modified-release capsule Oral
71374	Galpharm Ibuprofen For Children 100mg/5ml oral suspension 5ml sachets (Galpharm International Ltd) Ibuprofen 20mg/1ml Oral suspension Oral
66486	Arcoxia 90mg tablets (DE Pharmaceuticals) Etoricoxib 90mg Tablet Oral
14707	Defanac Retard 100mg tablets (Ranbaxy (UK) Ltd) Diclofenac sodium 100mg Modified-release tablet Oral
51769	Nurofen for Children Singles 100mg/5ml oral suspension 5ml sachets orange (Reckitt Benckiser Healthcare (UK) Ltd) Ibuprofen 20mg/1ml Oral suspension Oral
53617	Ibuprofen and codeine 200mg+12.8mg Tablet (Almus Pharmaceuticals Ltd) Ibuprofen/Codeine phosphate 200mg + 12.8mg Tablet Oral
53803	Ibuprofen 200mg capsules (Kent Pharmaceuticals Ltd) Ibuprofen 200mg Capsule Oral
31064	Mobiflex 20mg Granules (Roche Products Ltd) Tenoxicam 20mg Granules Oral

62238	Ibuprofen 200mg capsules (Colorama Pharmaceuticals Ltd) Ibuprofen 200mg Capsule Oral
31959	Indometacin 50mg capsules (A A H Pharmaceuticals Ltd) Indometacin 50mg Capsule Oral
597	Diclofenac potassium 50mg tablets Diclofenac potassium 50mg Tablet Oral
29068	Nurofen Extra Strength 400mg capsules (Reckitt Benckiser Healthcare (UK) Ltd) Ibuprofen 400mg Capsule Oral
44112	Voltarol Joint Pain 12.5mg tablets (Novartis Consumer Health UK Ltd) Diclofenac potassium 12.5mg Tablet Oral
11999	Orudis 100mg Capsule (Hawgreen Ltd) Ketoprofen 100mg Capsule Oral
5812	Etoricoxib 90mg tablets Etoricoxib 90mg Tablet Oral
5938	Etoricoxib 120mg tablets Etoricoxib 120mg Tablet Oral
73961	Naproxen 250mg tablets (Sigma Pharmaceuticals Plc) Naproxen 250mg Tablet Oral
55894	Naproxen 500mg gastro-resistant tablets (Phoenix Healthcare Distribution Ltd) Naproxen 500mg Gastro-resistant tablet Oral
67768	Naproxen 250mg tablets (Mylan) Naproxen 250mg Tablet Oral
52141	Mobilan 25mg Capsule (Galen Ltd) Indometacin 25mg Capsule Oral
650	Etoricoxib 60mg tablets Etoricoxib 60mg Tablet Oral
50117	Brufen 100mg/5ml syrup (Lexon (UK) Ltd) Ibuprofen 20mg/1ml Oral suspension Oral
76955	Naproxen 75mg/5ml oral suspension Naproxen 15mg/1ml Oral suspension Oral
5401	Voltarol Rapid 25mg tablets (Novartis Pharmaceuticals UK Ltd) Diclofenac potassium 25mg Tablet Oral
34271	Diclofenac sodium 100mg modified-release tablets (A A H Pharmaceuticals Ltd) Diclofenac sodium 100mg Modified-release tablet Oral
74216	Ibuprofen 100mg/5ml oral suspension sugar free (Pinewood Healthcare) Ibuprofen 20mg/1ml Oral suspension Oral
14541	Ponstan 50mg/5ml paediatric Liquid (Chemidex Pharma Ltd) Mefenamic acid 10mg/1ml Oral suspension Oral
29330	Diclofenac sodium 50mg gastro-resistant tablets (Sandoz Ltd) Diclofenac sodium 50mg Gastro-resistant tablet Oral
48546	Ibuprofen 400mg caplets (Bristol Laboratories Ltd) Ibuprofen 400mg Tablet Oral
31777	Piroxicam 20mg dispersible tablets (Mylan) Piroxicam 20mg Dispersible tablet Oral
66648	Ibuprofen 400mg tablets (Almus Pharmaceuticals Ltd) Ibuprofen 400mg Tablet Oral
5080	Celebrex 200mg capsules (Upjohn UK Ltd) Celecoxib 200mg Capsule Oral
14085	Diclovol Retard 100mg tablets (Arun Pharmaceuticals Ltd) Diclofenac sodium 100mg Modified-release tablet Oral
14776	Surgam 300mg tablets (Sanofi) Tiaprofenic acid 300mg Tablet Oral
34212	Diclofenac 75mg Modified-release tablet (Genus Pharmaceuticals Ltd) Diclofenac sodium 75mg Modified-release tablet Oral
26522	Meflam 500mg Tablet (Trinity Pharmaceuticals Ltd) Mefenamic acid 500mg Tablet Oral
56554	Naproxen 250mg/5ml oral suspension Naproxen 50mg/1ml Oral suspension Oral
73629	Naproxen 500mg tablets (Mylan) Naproxen 500mg Tablet Oral
27782	Ibuprofen 400mg tablets (Teva UK Ltd) Ibuprofen 400mg Tablet Oral
2197	Naproxen 375mg Tablet Naproxen 375mg Tablet Oral

28168	Nurofen Recovery 200mg orodispersible tablets (Reckitt Benckiser Healthcare (UK) Ltd) Ibuprofen 200mg Orodispersible tablet Oral
28900	Indometacin 25mg Capsule (Generics (UK) Ltd) Indometacin 25mg Capsule Oral
53331	Ibuprofen 100mg/5ml oral suspension sugar free (Alliance Healthcare (Distribution) Ltd) Ibuprofen 20mg/1ml Oral suspension Oral
50317	Voltarol 75mg SR tablets (Lexon (UK) Ltd) Diclofenac sodium 75mg Modified-release tablet Oral
67815	Feldene Melt 20mg tablets (Waymade Healthcare Plc) Piroxicam 20mg Orodispersible tablet Oral
48161	Naproxen 500mg Tablet (Almus Pharmaceuticals Ltd) Naproxen 500mg Tablet Oral
34447	Ibuprofen 200mg tablets (Thornton & Ross Ltd) Ibuprofen 200mg Tablet Oral
57045	Voltarol 50mg dispersible tablets (Waymade Healthcare Plc) Diclofenac sodium 50mg Dispersible tablet Oral
3901	Naprosyn EC 500mg tablets (Atnahs Pharma UK Ltd) Naproxen 500mg Gastro-resistant tablet Oral
1739	Brufen 400mg tablets (Mylan) Ibuprofen 400mg Tablet Oral
66247	Ibuprofen 100mg chewable capsules Ibuprofen 100mg Chewable capsule Oral
177	Indometacin 25mg capsules Indometacin 25mg Capsule Oral
1231	Ketoprofen 100mg capsules Ketoprofen 100mg Capsule Oral
296	Ponstan Forte 500mg tablets (Chemidex Pharma Ltd) Mefenamic acid 500mg Tablet Oral
39019	Brufen Retard 800mg tablets (Mylan) Ibuprofen 800mg Modified-release tablet Oral
33994	Diclofenac sodium 25mg gastro-resistant tablets (IVAX Pharmaceuticals UK Ltd) Diclofenac sodium 25mg Gastro-resistant tablet Oral
34910	Mefenamic acid 500mg Tablet (Berk Pharmaceuticals Ltd) Mefenamic acid 500mg Tablet Oral
17030	Rhumalgan SR 75mg capsules (Sandoz Ltd) Diclofenac sodium 75mg Modified-release capsule Oral
51339	Indometacin 25mg capsules (Genesis Pharmaceuticals Ltd) Indometacin 25mg Capsule Oral
17029	Rhumalgan CR 75 tablets (Sandoz Ltd) Diclofenac sodium 75mg Modified-release tablet Oral
65514	Ibuprofen 200mg tablets (Boston Healthcare Ltd) Ibuprofen 200mg Tablet Oral
66571	Celecoxib 200mg capsules (Actavis UK Ltd) Celecoxib 200mg Capsule Oral
42821	Nabumetone 500mg tablets (A A H Pharmaceuticals Ltd) Nabumetone 500mg Tablet Oral
1983	Mefenamic acid 250mg Dispersible tablet Mefenamic Acid 250mg Dispersible Tablet Oral
42397	Nurofen Express 256mg tablets (Reckitt Benckiser Healthcare (UK) Ltd) Ibuprofen sodium dihydrate 200mg Tablet Oral
1246	Ponstan 250mg Dispersible tablet (Chemidex Pharma Ltd) Mefenamic Acid 250mg Dispersible Tablet Oral
586	Ibuprofen 200mg Capsule Ibuprofen 200mg Capsule Oral
41366	Axorid 100mg/20mg modified-release capsules (Meda Pharmaceuticals Ltd) Ketoprofen/Omeprazole 100mg + 20mg Modified-release capsule Oral
74835	Diclomax Retard 100mg capsules (DE Pharmaceuticals) Diclofenac sodium 100mg Modified-release capsule Oral

3817	Synflex 275mg tablets (Roche Products Ltd) Naproxen sodium 275mg Tablet Oral
26351	Rheumatac Retard 75 tablets (Advanz Pharma) Diclofenac sodium 75mg Modified-release tablet Oral
62643	Indometacin 50mg Capsule (Carter Wallace Ltd) Indometacin 50mg Capsule Oral
50266	Ibuprofen 200mg caplets (The Boots Company Plc) Ibuprofen 200mg Tablet Oral
77335	Nurofen 400mg Granules (Crookes Healthcare Ltd) Ibuprofen 400mg Granules
14422	Fenbufen 450mg Effervescent tablet Fenbufen 450mg Effervescent Tablet Oral
57112	Ibuprofen 400mg tablets (Alliance Healthcare (Distribution) Ltd) Ibuprofen 400mg Tablet Oral
8062	Motifene 75mg modified-release capsules (Daiichi Sankyo UK Ltd) Diclofenac sodium 75mg Modified-release capsule Oral
32854	Diclofenac sodium 75mg modified-release capsules (A A H Pharmaceuticals Ltd) Diclofenac sodium 75mg Modified-release capsule Oral
51874	Arcoxia 30mg tablets (Lexon (UK) Ltd) Etoricoxib 30mg Tablet Oral
48871	Diclofenac potassium 25mg tablets (Accord Healthcare Ltd) Diclofenac potassium 25mg Tablet Oral
76404	Naproxen 500mg gastro-resistant tablets (DE Pharmaceuticals) Naproxen 500mg Gastro-resistant tablet Oral
3897	Sulindac 100mg tablets Sulindac 100mg Tablet Oral
26242	Timpron 250mg Tablet (Berk Pharmaceuticals Ltd) Naproxen 250mg Tablet Oral
29352	Ibuprofen 100mg/5ml oral suspension sugar free (Vantage) Ibuprofen 20mg/1ml Oral suspension Oral
52154	Ibuprofen 200mg tablets (Galpharm International Ltd) Ibuprofen 200mg Tablet Oral
64759	Diclofenac 50mg/5ml oral solution Diclofenac sodium 10mg/1ml Oral solution Oral
75154	Mefenamic acid 250mg/5ml oral suspension Mefenamic acid 50mg/1ml Oral suspension Oral
10209	Ibufem 200mg tablets (Galpharm International Ltd) Ibuprofen 200mg Tablet Oral
55233	Ibuprofen 400mg Tablet (Nucare Plc) Ibuprofen 400mg Tablet Oral
69645	Naproxen 50mg/ml oral suspension (Thornton & Ross Ltd) Naproxen 50mg/1ml Oral suspension Oral
60035	Ibuprofen 200mg tablets film coated (Actavis UK Ltd) Ibuprofen 200mg Tablet Oral
4469	Fenoprofen 300mg tablets Fenoprofen calcium 300mg Tablet Oral
28519	Pseudoephedrine 30mg with ibuprofen 200mg tablet Ibuprofen/Pseudoephedrine Hydrochloride 30mg + 200mg Tablets Oral
44800	Naproxen 500mg / Esomeprazole 20mg modified-release tablets Naproxen/Esomeprazole magnesium trihydrate 500mg + 20mg Modified-release tablet Oral
17680	Indomax 75 SR capsules (Ashbourne Pharmaceuticals Ltd) Indometacin 75mg Modified-release capsule Oral
50363	Nurofen for Children Singles 100mg/5ml oral suspension 5ml sachets strawberry (Reckitt Benckiser Healthcare (UK) Ltd) Ibuprofen 20mg/1ml Oral suspension Oral
4045	Naprosyn EC 375mg tablets (Atnahs Pharma UK Ltd) Naproxen 375mg Gastro-resistant tablet Oral

79845	Diclofenac sodium 50mg gastro-resistant tablets (Crescent Pharma Ltd) Diclofenac sodium 50mg Gastro-resistant tablet Oral
26247	Opustan 500mg Tablet (Opus Pharmaceuticals Ltd) Mefenamic acid 500mg Tablet Oral
22230	Meflam 250mg Capsule (Trinity Pharmaceuticals Ltd) Mefenamic acid 250mg Capsule Oral
33785	Galprofen 200mg tablets (Galpharm International Ltd) Ibuprofen 200mg Tablet Oral
32875	Ibuprofen 400mg tablets (Sandoz Ltd) Ibuprofen 400mg Tablet Oral
30942	Diclofenac 50mg Tablet (Regent Laboratories Ltd) Diclofenac sodium 50mg Gastro-resistant tablet Oral
50059	Celebrex 100mg capsules (Necessity Supplies Ltd) Celecoxib 100mg Capsule Oral
31916	Tiloket CR 100mg capsules (Tillomed Laboratories Ltd) Ketoprofen 100mg Modified-release capsule Oral
54906	Diclofenac 50mg/5ml oral suspension Diclofenac sodium 10mg/1ml Oral suspension Oral
56213	Ibuprofen 400mg tablets sugar coated (Kent Pharmaceuticals Ltd) Ibuprofen 400mg Tablet Oral
79577	Naproxen 250mg tablets (Genesis Pharmaceuticals Ltd) Naproxen 250mg Tablet Oral
48644	Ibuprofen 400mg caplets (Lloyds Pharmacy Ltd) Ibuprofen 400mg Tablet Oral
6249	Froben 100mg tablets (Abbott Laboratories Ltd) Flurbiprofen 100mg Tablet Oral
55505	Naproxen 250mg gastro-resistant tablets (Kent Pharmaceuticals Ltd) Naproxen 250mg Gastro-resistant tablet Oral
8401	Motrin 400mg tablets (Pfizer Ltd) Ibuprofen 400mg Tablet Oral
41624	Piroxicam 10mg capsules (IVAX Pharmaceuticals UK Ltd) Piroxicam 10mg Capsule Oral
1115	Diclofenac sodium 100mg modified-release capsules Diclofenac sodium 100mg Modified-release capsule Oral
61596	Diclofenac sodium 75mg modified-release capsules (Phoenix Healthcare Distribution Ltd) Diclofenac sodium 75mg Modified-release capsule Oral
25342	Arthrosin EC 500 tablets (Ashbourne Pharmaceuticals Ltd) Naproxen 500mg Gastro-resistant tablet Oral
74048	Diclomax Retard 100mg capsules (Mawdsley-Brooks & Company Ltd) Diclofenac sodium 100mg Modified-release capsule Oral
18647	Fenoket 200mg modified-release capsules (Opus Pharmaceuticals Ltd) Ketoprofen 200mg Modified-release capsule Oral
32097	Indometacin 75mg Modified-release capsule (Actavis UK Ltd) Indometacin 75mg Modified-release capsule Oral
34289	Naproxen 250mg gastro-resistant tablets (Mylan) Naproxen 250mg Gastro-resistant tablet Oral
2129	Brufen retard tabs 800mg Modified-release tablet (Abbott Laboratories Ltd) Ibuprofen 800mg Modified-release tablet Oral
28348	Ibuprofen 200mg tablets (Teva UK Ltd) Ibuprofen 200mg Tablet Oral
49432	Calprofen 100mg/5ml oral suspension (McNeil Products Ltd) Ibuprofen 20mg/1ml Oral suspension Oral
76284	Ibular 200mg tablets (Ennogen Pharma Ltd) Ibuprofen 200mg Tablet Oral
9474	Preservex 100mg tablets (Almirall Ltd) Aceclofenac 100mg Tablet Oral
65783	Diclofenac potassium 50mg tablets (DE Pharmaceuticals) Diclofenac potassium 50mg Tablet Oral

65591	Ibuprofen 200mg tablets (Crescent Pharma Ltd) Ibuprofen 200mg Tablet Oral
32862	Ibuprofen 100mg/5ml oral suspension sugar free (Thornton & Ross Ltd) Ibuprofen 20mg/1ml Oral suspension Oral
17201	Motrin 600mg tablets (Pfizer Ltd) Ibuprofen 600mg Tablet Oral
4298	Nurofen 200mg Tablet (Crookes Healthcare Ltd) Ibuprofen 200mg Tablet Oral
72293	Celecoxib 200mg capsules (Mylan) Celecoxib 200mg Capsule Oral
25341	Arthrosin EC 250 tablets (Ashbourne Pharmaceuticals Ltd) Naproxen 250mg Gastro-resistant tablet Oral
57943	Valket 200 Retard capsules (Tillomed Laboratories Ltd) Ketoprofen 200mg Modified-release capsule Oral
78675	Diclofenac sodium 100mg modified-release capsules (Sigma Pharmaceuticals Plc) Diclofenac sodium 100mg Modified-release capsule Oral
66577	Diclofenac sodium 75mg modified-release capsules (Sigma Pharmaceuticals Plc) Diclofenac sodium 75mg Modified-release capsule Oral
34911	Ibuprofen 200mg Tablet (Celltech Pharma Europe Ltd) Ibuprofen 200mg Tablet Oral
14476	Indolar SR 75mg capsules (Sandoz Ltd) Indometacin 75mg Modified-release capsule Oral
38770	Lodine SR 600mg tablets (Almirall Ltd) Etodolac 600mg Modified-release tablet Oral
1470	Mobic 15mg tablets (Boehringer Ingelheim Ltd) Meloxicam 15mg Tablet Oral
3599	Ibuprofen 600mg effervescent granules sachets Ibuprofen 600mg Effervescent granules Oral
76610	Indometacin 25mg/5ml oral suspension Indometacin 5mg/1ml Oral suspension Oral
61953	Ibuprofen 200mg caplets (Wockhardt UK Ltd) Ibuprofen 200mg Tablet Oral
2288	Naprosyn 250mg tablets (Atnahs Pharma UK Ltd) Naproxen 250mg Tablet Oral
51808	Diclofenac 12.5mg/5ml oral solution Diclofenac sodium 2.5mg/1ml Oral solution Oral
3266	Flurbiprofen 50mg tablets Flurbiprofen 50mg Tablet Oral
75549	Etoricoxib 30mg tablets (Accord Healthcare Ltd) Etoricoxib 30mg Tablet Oral
11907	Dexibuprofen 400mg tablets Dexibuprofen 400mg Tablet Oral
75893	Ibuprofen 200mg/5ml oral suspension sugar free Ibuprofen 40mg/1ml Oral suspension Oral
37562	Arcoxia 30mg tablets (Merck Sharp & Dohme Ltd) Etoricoxib 30mg Tablet Oral
30297	Diclofenac 50mg Gastro-resistant tablet (Pharmacia Ltd) Diclofenac sodium 50mg Gastro-resistant tablet Oral
34354	Ibuprofen 200mg tablets (Vantage) Ibuprofen 200mg Tablet Oral
30724	Galprofen 100mg/5ml oral suspension (Galpharm International Ltd) Ibuprofen 20mg/1ml Oral suspension Oral
59067	Ibuprofen 200mg capsules (AM Distributions (Yorkshire) Ltd) Ibuprofen 200mg Capsule Oral
65348	Naproxen 250mg gastro-resistant tablets (Genesis Pharmaceuticals Ltd) Naproxen 250mg Gastro-resistant tablet Oral
2258	Emflex 60mg capsules (Merck Serono Ltd) Acemetacin 60mg Capsule Oral
38817	Diclofenac potassium 12.5mg tablets Diclofenac potassium 12.5mg Tablet Oral
57475	Meloxicam 7.5mg orodispersible tablets sugar free Meloxicam 7.5mg Orodispersible tablet Oral
54476	Naproxen 500mg gastro-resistant tablets (Genesis Pharmaceuticals Ltd) Naproxen 500mg Gastro-resistant tablet Oral

24121	Diclofenac sodium 25mg gastro-resistant tablets (Actavis UK Ltd) Diclofenac sodium 25mg Gastro-resistant tablet Oral
34487	Diclofenac sodium 50mg gastro-resistant tablets (IVAX Pharmaceuticals UK Ltd) Diclofenac sodium 50mg Gastro-resistant tablet Oral
1608	Adcortyl with graneodin Cream (E R Squibb and Sons Ltd) Neomycin Sulphate/Gramicidin/Triamcinolone Acetonide Cream Topical
59502	Nurofen for Children Cold, Pain and Fever Strawberry Flavour 100mg/5ml oral suspension (Reckitt Benckiser Healthcare (UK) Ltd) Ibuprofen 20mg/1ml Oral suspension Oral
71314	Ibuprofen 200mg tablets (Kent Pharmaceuticals Ltd) Ibuprofen 200mg Tablet Oral
58652	Ibuprofen 600mg tablets (Sigma Pharmaceuticals Plc) Ibuprofen 600mg Tablet Oral
25619	Nurofen 400mg Tablet (Crookes Healthcare Ltd) Ibuprofen 400mg Tablet Oral
58071	Voltarol Rapid 50mg tablets (Waymade Healthcare Plc) Diclofenac potassium 50mg Tablet Oral
76419	Etodolac sr 600mg Tablet (Winthrop Pharmaceuticals Ltd) Etodolac 600mg Modified-release tablet Oral
56078	Rhumalgan XL 100mg capsules (Almus Pharmaceuticals Ltd) Diclofenac sodium 100mg Modified-release capsule Oral
75120	Diflunisal 250mg Tablet (Approved Prescription Services Ltd) Diflunisal 250mg Tablet Oral
21824	Flamrase 50 EC tablets (Teva UK Ltd) Diclofenac sodium 50mg Gastro-resistant tablet Oral
66364	Meloxicam 15mg tablets (Actavis UK Ltd) Meloxicam 15mg Tablet Oral
25205	Ibuprofen 100mg/5ml oral suspension 5ml sachets sugar free (Thornton & Ross Ltd) Ibuprofen 20mg/1ml Oral suspension Oral
21813	Lidifen 400mg Tablet (Berk Pharmaceuticals Ltd) Ibuprofen 400mg Tablet Oral
21955	Ketozip 200 XL capsules (Ashbourne Pharmaceuticals Ltd) Ketoprofen 200mg Modified-release capsule Oral
44233	Nurofen for children baby 100mg/5ml Oral suspension (Reckitt Benckiser Healthcare (UK) Ltd) Ibuprofen 100mg/5ml Oral Suspension Oral
35292	Nurofen 200mg liquid capsules (Reckitt Benckiser Healthcare (UK) Ltd) Ibuprofen 200mg Capsule Oral
28553	Diclofenac sodium 50mg gastro-resistant tablets (Teva UK Ltd) Diclofenac sodium 50mg Gastro-resistant tablet Oral
24887	Nurofen Advance 200mg tablets (Crookes Healthcare Ltd) Ibuprofen lysine 200mg Tablet Oral
71908	Etolyn 600mg modified-release tablets (Mylan) Etodolac 600mg Modified-release tablet Oral
42455	Dicloflex Retard 100mg tablets (Teva UK Ltd) Diclofenac sodium 100mg Modified-release tablet Oral
72396	Diclofenac sodium 50mg gastro-resistant tablets (Medreich Plc) Diclofenac sodium 50mg Gastro-resistant tablet Oral
71152	Mobiflex 20mg tablets (Dowelhurst Ltd) Tenoxicam 20mg Tablet Oral
51343	Voltarol Rapid 25mg tablets (DE Pharmaceuticals) Diclofenac potassium 25mg Tablet Oral
29332	Ibuprofen 100mg/5ml oral suspension sugar free (Sandoz Ltd) Ibuprofen 20mg/1ml Oral suspension Oral
71041	Ibuprofen 600mg tablets (Bristol Laboratories Ltd) Ibuprofen 600mg Tablet Oral

17128	Fenactol 50mg gastro-resistant tablets (Dexcel-Pharma Ltd) Diclofenac sodium 50mg Gastro-resistant tablet Oral
11980	Cuprofen 400mg Tablet (SSL International Plc) Ibuprofen 400mg Tablet Oral
45213	Diclofenac 10mg dispersible tablets Diclofenac sodium 10mg Dispersible tablet Oral
580	Diclofenac sodium 75mg modified-release tablets Diclofenac sodium 75mg Modified-release tablet Oral
70299	Ibuprofen 200mg capsules (Bell,Sons & Co (Druggists) Ltd) Ibuprofen 200mg Capsule Oral
45262	Naproxen Oral solution Naproxen Oral Solution Oral
19575	Proflex 200mg Tablet (Novartis Consumer Health UK Ltd) Ibuprofen 200mg Tablet Oral
23026	Artracin sr 75mg Modified-release capsule (Trinity Pharmaceuticals Ltd) Indometacin 75mg Modified-release capsule Oral
55434	Ibuprofen 400mg tablets (Bristol Laboratories Ltd) Ibuprofen 400mg Tablet Oral
62843	Arcoxia 90mg tablets (Lexon (UK) Ltd) Etoricoxib 90mg Tablet Oral
28390	Valenac ec 25mg Gastro-resistant tablet (Shire Pharmaceuticals Ltd) Diclofenac sodium 25mg Gastro-resistant tablet Oral
32108	Diclofenac sodium 25mg gastro-resistant tablets (Teva UK Ltd) Diclofenac sodium 25mg Gastro-resistant tablet Oral
65471	Ibuprofen 200mg tablets (Almus Pharmaceuticals Ltd) Ibuprofen 200mg Tablet Oral
55454	Naproxen 500mg tablets (Kent Pharmaceuticals Ltd) Naproxen 500mg Tablet Oral
10481	Lederfen f 450mg Tablet (Wyeth Pharmaceuticals) Fenbufen 450mg Tablet Oral
72030	Naproxen 500mg tablets (Sigma Pharmaceuticals Plc) Naproxen 500mg Tablet Oral
18371	Digenac xl 100mg Modified-release tablet (Genus Pharmaceuticals Ltd) Diclofenac sodium 100mg Modified-release tablet Oral
34621	Ibuprofen 200mg Tablet (Nucare Plc) Ibuprofen 200mg Tablet Oral
49059	Voltarol 50mg dispersible tablets (Lexon (UK) Ltd) Diclofenac sodium 50mg Dispersible tablet Oral
21050	Ketonal 100mg Capsule (Lagap) Ketoprofen 100mg Capsule Oral
30282	Diclofenac 75mg Modified-release tablet (Galen Ltd) Diclofenac sodium 75mg Modified-release tablet Oral
53604	Ibuprofen 200mg capsules (Numark Ltd) Ibuprofen 200mg Capsule Oral
27200	Diclovol Retard 100mg tablets (Mylan) Diclofenac sodium 100mg Modified-release tablet Oral
2243	Meloxicam 7.5mg tablets Meloxicam 7.5mg Tablet Oral
47816	Tenoxicam 20mg Tablet (Sovereign Medical Ltd) Tenoxicam 20mg Tablet Oral
1043	Naproxen sodium 275mg tablets Naproxen sodium 275mg Tablet Oral
19322	Disalcid 500mg Capsule (3M Health Care Ltd) Salsalate 500mg Capsule Oral
25362	Defanac 25mg gastro-resistant tablets (Ranbaxy (UK) Ltd) Diclofenac sodium 25mg Gastro-resistant tablet Oral
61695	Naproxen 500mg tablets (Alliance Healthcare (Distribution) Ltd) Naproxen 500mg Tablet Oral
77228	Ibuprofen 200mg Tablet (LPC Medical (UK) Ltd) Ibuprofen 200mg Tablet Oral
49277	Ibuprofen 200mg caplets (Bristol Laboratories Ltd) Ibuprofen 200mg Tablet Oral
1210	Indometacin 75mg modified-release capsules Indometacin 75mg Modified-release capsule Oral

36606	Manorfen 400mg tablets (The Manor Drug Company (Nottingham) Ltd) Ibuprofen 400mg Tablet Oral
75573	Ketoprofen 100mg modified-release capsules (A A H Pharmaceuticals Ltd) Ketoprofen 100mg Modified-release capsule Oral
41823	Indometacin sr 75mg Modified-release capsule (Generics (UK) Ltd) Indometacin 75mg Modified-release capsule Oral
1096	Diclofenac sodium 25mg gastro-resistant tablets Diclofenac Sodium 25mg EC Tablets Oral
69828	Naproxen 50mg/ml oral suspension (Alliance Healthcare (Distribution) Ltd) Naproxen 50mg/1ml Oral suspension Oral
838	Oruvail 200mg Modified-release capsule (Hawgreen Ltd) Ketoprofen 200mg Modified-release capsule Oral
62658	Arcoxia 120mg tablets (Waymade Healthcare Plc) Etoricoxib 120mg Tablet Oral
1075	Diclofenac sodium 50mg gastro-resistant tablets Diclofenac Sodium 50mg EC Tablets Oral
16272	Lofensaid Retard 100 tablets (Opus Pharmaceuticals Ltd) Diclofenac sodium 100mg Modified-release tablet Oral
33589	Ibuprofen 400mg tablets (Thornton & Ross Ltd) Ibuprofen 400mg Tablet Oral
46919	Ketoprofen sr 200mg Capsule (Approved Prescription Services Ltd) Ketoprofen 200mg Modified-release capsule Oral
79167	Ibuprofen 400mg Tablet (Teva UK Ltd) Ibuprofen 400mg Tablet Oral
50269	Arthrotec 75 gastro-resistant tablets (Mawdsley-Brooks & Company Ltd) Diclofenac sodium/Misoprostol 75mg + 200microgram Gastro-resistant tablet Oral
32704	Advil cold and sinus 200mg+30mg Tablet (Wyeth Consumer Healthcare) Ibuprofen/Pseudoephedrine hydrochloride 200mg + 30mg Tablet Oral
39502	Ibuprofen sodium dihydrate 200mg tablets Ibuprofen sodium dihydrate 200mg Tablet Oral
70438	Arthrotec 50 gastro-resistant tablets (Lexon (UK) Ltd) Diclofenac sodium/Misoprostol 50mg + 200microgram Gastro-resistant tablet Oral
42406	Diclofenac 50mg Gastro-resistant tablet (Almus Pharmaceuticals Ltd) Diclofenac sodium 50mg Gastro-resistant tablet Oral
25790	Rhumalgan 25mg Tablet (Lagap) Diclofenac sodium 25mg Gastro-resistant tablet Oral
76234	Ibuprofen Twelve Plus Pain Relief 200mg/5ml oral suspension (Aspire Pharma Ltd) Ibuprofen 40mg/1ml Oral suspension Oral
68470	Naproxen 25mg/ml oral suspension sugar free (Orion Pharma (UK) Ltd) Naproxen 25mg/1ml Oral suspension Oral
33111	Prosaid 250mg Tablet (BHR Pharmaceuticals Ltd) Naproxen 250mg Tablet Oral
16194	Lodine 200mg Tablet (Shire Pharmaceuticals Ltd) Etodolac 200mg Tablet Oral
2938	Ibuprofen 100mg/5ml Oral suspension Ibuprofen 100mg/5ml Oral Suspension Oral
37253	Anadin ultra double strength 400mg Capsule (Wyeth Consumer Healthcare) Ibuprofen 400mg Capsule Oral
56106	Naproxen 500mg/5ml oral suspension Naproxen 100mg/1ml Oral suspension Oral
69285	Boots Ibuprofen and Codeine 200mg/12.8mg tablets (The Boots Company Plc) Ibuprofen/Codeine phosphate 200mg + 12.8mg Tablet Oral
1778	Surgam 300mg Tablet (Sanofi) Tiaprofenic acid 300mg Tablet Oral

58221	Naproxen 250mg gastro-resistant tablets (Accord Healthcare Ltd) Naproxen 250mg Gastro-resistant tablet Oral
24212	Imbrilon 50mg Capsule (Berk Pharmaceuticals Ltd) Indometacin 50mg Capsule Oral
29587	Ebufac 400mg Tablet (DDSA Pharmaceuticals Ltd) Ibuprofen 400mg Tablet Oral
64245	Celecoxib 200mg capsules (A A H Pharmaceuticals Ltd) Celecoxib 200mg Capsule Oral
68582	Ibuprofen 200mg tablets (DE Pharmaceuticals) Ibuprofen 200mg Tablet Oral
1233	Diclofenac sodium 75mg modified-release tablets Diclofenac Sodium 75mg Modified Release Tablet Oral
19046	Ibuprofen 400mg tablets (A A H Pharmaceuticals Ltd) Ibuprofen 400mg Tablet Oral
36486	Econac XL 100mg tablets (Advanz Pharma) Diclofenac sodium 100mg Modified-release tablet Oral
48084	Ibuprofen 200mg/5ml oral suspension Ibuprofen 40mg/1ml Oral suspension Oral
74313	Brufen Retard 800mg tablets (Lexon (UK) Ltd) Ibuprofen 800mg Modified-release tablet Oral
4731	Nurofen for children 100mg/5ml Oral suspension (Reckitt Benckiser Healthcare (UK) Ltd) Ibuprofen 100mg/5ml Oral Suspension Oral
15068	Arthrofen 400 tablets (Ashbourne Pharmaceuticals Ltd) Ibuprofen 400mg Tablet Oral
4692	Dicloflex 50mg gastro-resistant tablets (Dexcel-Pharma Ltd) Diclofenac sodium 50mg Gastro-resistant tablet Oral
46440	Naproxen 500mg Tablet (M & A Pharmachem Ltd) Naproxen 500mg Tablet Oral
68354	Diclofenac sodium 100mg modified-release capsules (Actavis UK Ltd) Diclofenac sodium 100mg Modified-release capsule Oral
51099	Voltarol Rapid 50mg tablets (Mawdsley-Brooks & Company Ltd) Diclofenac potassium 50mg Tablet Oral
21421	Seractil 400mg tablets (Thornton & Ross Ltd) Dexibuprofen 400mg Tablet Oral
20805	Dicloflex 75mg SR tablets (Teva UK Ltd) Diclofenac sodium 75mg Modified-release tablet Oral
24128	Diclofenac sodium 25mg gastro-resistant tablets (A A H Pharmaceuticals Ltd) Diclofenac sodium 25mg Gastro-resistant tablet Oral
141	Piroxicam 10mg capsules Piroxicam 10mg Capsule Oral
26214	Fenbuzip 450mg Tablet (Ashbourne Pharmaceuticals Ltd) Fenbufen 450mg Tablet Oral
8789	Dicloflex retard tabs 100 100mg Modified-release tablet (Dexcel-Pharma Ltd) Diclofenac sodium 100mg Modified-release tablet Oral
77405	Voltarol Retard 100mg tablets (Waymade Healthcare Plc) Diclofenac sodium 100mg Modified-release tablet Oral
21807	Flamrase 25 EC tablets (Teva UK Ltd) Diclofenac sodium 25mg Gastro-resistant tablet Oral
45320	Ibuprofen 200mg tablets (Sandoz Ltd) Ibuprofen 200mg Tablet Oral
71117	Diclomax SR 75mg capsules (DE Pharmaceuticals) Diclofenac sodium 75mg Modified-release capsule Oral
72546	Diclofenac sodium 100mg modified-release capsules (A A H Pharmaceuticals Ltd) Diclofenac sodium 100mg Modified-release capsule Oral
26404	Tolmetin 200mg Capsule Tolmetin Sodium 200mg Capsule Oral
1621	Brufen 200mg tablets (Abbott Laboratories Ltd) Ibuprofen 200mg Tablet Oral

54514	Ibuprofen lysine 400mg oral powder sachets Ibuprofen lysine 400mg Powder Oral
68932	Meloxicam 15mg tablets (Somex Pharma) Meloxicam 15mg Tablet Oral
79173	Mefenamic acid 250mg capsules (Advanz Pharma) Mefenamic acid 250mg Capsule Oral
4710	Mefenamic acid 250mg Capsule (Actavis UK Ltd) Mefenamic acid 250mg Capsule Oral
4564	Fenoprofen 200mg Tablet Fenoprofen Calcium 200mg Tablet Oral
71072	Ibuprofen 100mg/5ml oral suspension sugar free (Phoenix Healthcare Distribution Ltd) Ibuprofen 20mg/1ml Oral suspension Oral
21444	Volraman 25mg gastro-resistant tablets (LPC Medical (UK) Ltd) Diclofenac sodium 25mg Gastro-resistant tablet Oral
12188	Trilisate 500mg Tablet (Napp Pharmaceuticals Ltd) Magnesium Trisilicate 500mg Tablet Oral
24236	Slofenac 100mg Modified-release tablet (Sterwin Medicines) Diclofenac sodium 100mg Modified-release tablet Oral
66757	Celebrex 200mg capsules (Waymade Healthcare Plc) Celecoxib 200mg Capsule Oral
71100	Diclofenac sodium 50mg gastro-resistant tablets (Sigma Pharmaceuticals Plc) Diclofenac sodium 50mg Gastro-resistant tablet Oral
46860	Anadin LiquiFast 200mg effervescent tablets (Pfizer Consumer Healthcare Ltd) Ibuprofen 200mg Effervescent tablet Oral
64297	Nabumetone 500mg tablets (Mylan) Nabumetone 500mg Tablet Oral
19036	Arthrofen 200 tablets (Ashbourne Pharmaceuticals Ltd) Ibuprofen 200mg Tablet Oral
60666	Diclofenac sodium 75mg modified-release capsules (Actavis UK Ltd) Diclofenac sodium 75mg Modified-release capsule Oral
19320	Piroflam 20mg Capsule (Opus Pharmaceuticals Ltd) Piroxicam 20mg Capsule Oral
17754	Progesic 200mg Tablet (Eli Lilly and Company Ltd) Fenoprofen Calcium 200mg Tablet Oral
70468	Voltarol Rapid 50mg tablets (Sigma Pharmaceuticals Plc) Diclofenac potassium 50mg Tablet Oral
64935	Celecoxib 200mg capsules (Sigma Pharmaceuticals Plc) Celecoxib 200mg Capsule Oral
18921	Fenactol 25mg gastro-resistant tablets (Discovery Pharmaceuticals) Diclofenac sodium 25mg Gastro-resistant tablet Oral
497	Voltarol 25mg gastro-resistant tablets (Novartis Pharmaceuticals UK Ltd) Diclofenac sodium 25mg Gastro-resistant tablet Oral
32366	Relcofen 200mg Tablet (Actavis UK Ltd) Ibuprofen 200mg Tablet Oral
21864	Pirozip 10 capsules (Ashbourne Pharmaceuticals Ltd) Piroxicam 10mg Capsule Oral
52931	Naproxen 500mg gastro-resistant tablets (Kent Pharmaceuticals Ltd) Naproxen 500mg Gastro-resistant tablet Oral
4043	Froben sr 200mg Modified-release capsule (Abbott Laboratories Ltd) Flurbiprofen 200mg Modified-release capsule Oral
43616	Celecoxib 400mg capsules Celecoxib 400mg Capsule Oral
9500	Diclotard 75mg modified-release tablets (Galen Ltd) Diclofenac sodium 75mg Modified-release tablet Oral

26888	Difenor xl 100mg Modified-release tablet (IVAX Pharmaceuticals UK Ltd) Diclofenac sodium 100mg Modified-release tablet Oral
16192	Motrin 200mg Tablet (Pharmacia Ltd) Ibuprofen 200mg Tablet Oral
48810	Dysman 250 capsules (Ashbourne Pharmaceuticals Ltd) Mefenamic acid 250mg Capsule Oral
7522	Lederfen 300mg Tablet (Wyeth Pharmaceuticals) Fenbufen 300mg Tablet Oral
3432	Naproxen 375mg gastro-resistant tablets Naproxen 375mg Gastro-resistant tablet Oral
43911	Ibuprofen 600mg Tablet (C P Pharmaceuticals Ltd) Ibuprofen 600mg Tablet Oral
41621	Piroxicam 20mg capsules (A A H Pharmaceuticals Ltd) Piroxicam 20mg Capsule Oral
30243	Ibuprofen 200mg effervescent tablets Ibuprofen 200mg Effervescent tablet Oral
33113	Artracin 50mg Capsule (DDSA Pharmaceuticals Ltd) Indometacin 50mg Capsule Oral
67756	Indometacin 25mg capsules (Almus Pharmaceuticals Ltd) Indometacin 25mg Capsule Oral
46968	Mefenamic acid 250mg capsules (Mylan) Mefenamic acid 250mg Capsule Oral
53384	Voltarol 50mg dispersible tablets (Mawdsley-Brooks & Company Ltd) Diclofenac sodium 50mg Dispersible tablet Oral
34610	Naproxen 500mg gastro-resistant tablets (Mylan) Naproxen 500mg Gastro-resistant tablet Oral
26994	Fenbuzip 300mg Tablet (Ashbourne Pharmaceuticals Ltd) Fenbufen 300mg Tablet Oral
33704	Ibuprofen 100mg/5ml oral suspension sugar free (A A H Pharmaceuticals Ltd) Ibuprofen 20mg/1ml Oral suspension Oral
25358	Defanac 50mg gastro-resistant tablets (Ranbaxy (UK) Ltd) Diclofenac sodium 50mg Gastro-resistant tablet Oral
402	Nurofen 200mg Tablet (Crookes Healthcare Ltd) Ibuprofen 200mg Tablet Oral
63357	Naproxen 250mg tablets (Almus Pharmaceuticals Ltd) Naproxen 250mg Tablet Oral
29345	Ibuprofen 100mg/5ml Oral suspension (Hillcross Pharmaceuticals Ltd) Ibuprofen 20mg/1ml Oral suspension Oral
65121	Fenpaed 100mg/5ml oral suspension (Pinewood Healthcare) Ibuprofen 20mg/1ml Oral suspension Oral
344	Acemetacin 60mg capsules Acemetacin 60mg Capsule Oral
60916	Indometacin 75mg modified-release capsules (Kent Pharmaceuticals Ltd) Indometacin 75mg Modified-release capsule Oral
34663	Ibuprofen 100mg/5ml Oral suspension (Neo Laboratories Ltd) Ibuprofen 20mg/1ml Oral suspension Oral
71127	Oruvail 100 modified-release capsules (Waymade Healthcare Plc) Ketoprofen 100mg Modified-release capsule Oral
30122	Lornoxicam 8mg tablets Lornoxicam 8mg Tablet Oral
29010	Phenylbutazone 100mg tablets Phenylbutazone 100mg Tablet Oral
42500	Ketoprofen sr 100mg Capsule (Approved Prescription Services Ltd) Ketoprofen 100mg Modified-release capsule Oral
31589	Diclofenac sodium 75mg modified-release tablets (A A H Pharmaceuticals Ltd) Diclofenac sodium 75mg Modified-release tablet Oral
8663	Naprosyn S/R 500mg tablets (Roche Products Ltd) Naproxen sodium 500mg Modified-release tablet Oral

38948	Diclomax Retard 100mg capsules (Galen Ltd) Diclofenac sodium 100mg Modified-release capsule Oral
76310	Mefenamic acid 500mg/5ml oral suspension Mefenamic acid 100mg/1ml Oral suspension Oral
44892	Sudafed sinus pressure & pain Tablet (McNeil Products Ltd) Ibuprofen/Pseudoephedrine hydrochloride 200mg + 30mg Tablet Oral
77525	Ibuprofen 200mg tablets (Bell,Sons & Co (Druggists) Ltd) Ibuprofen 200mg Tablet Oral
39722	Voltarol Pain-eze 12.5mg tablets (Novartis Consumer Health UK Ltd) Diclofenac potassium 12.5mg Tablet Oral
35893	Dicloflex Retard 100mg tablets (Almus Pharmaceuticals Ltd) Diclofenac sodium 100mg Modified-release tablet Oral
77185	Piroxicam 20mg Capsule (Ashbourne Pharmaceuticals Ltd) Piroxicam 20mg Capsule Oral
11322	Flamrase sr 75mg Modified-release tablet (APS Berk) Diclofenac sodium 75mg Modified-release tablet Oral
5407	Naproxen 125mg/5ml oral suspension Naproxen 25mg/1ml Oral suspension Oral
52009	Ibuprofen 200mg capsules (Galpharm International Ltd) Ibuprofen 200mg Capsule Oral
20386	Ramodar 200mg Tablet (Wyeth Pharmaceuticals) Etodolac 200mg Tablet Oral
69018	Nurofen Joint & Back Pain Relief 256mg tablets (Reckitt Benckiser Healthcare (UK) Ltd) Ibuprofen sodium dihydrate 200mg Tablet Oral
850	Mobic 7.5mg tablets (Boehringer Ingelheim Ltd) Meloxicam 7.5mg Tablet Oral
33318	Indometacin 50mg Capsule (Generics (UK) Ltd) Indometacin 50mg Capsule Oral
41817	Indometacin sr 75mg Modified-release capsule (C P Pharmaceuticals Ltd) Indometacin 75mg Modified-release capsule Oral
1392	Ibuprofen 800mg modified-release tablets Ibuprofen 800mg Modified-release tablet Oral
15	Ibuprofen 400mg tablets Ibuprofen 400mg Tablet Oral
25794	Isisfen 400mg Tablet (Isis Products Ltd) Ibuprofen 400mg Tablet Oral
75771	Larafen 100mg Capsule (Sandoz Ltd) Ketoprofen 100mg Capsule Oral
3053	Naproxen 500mg gastro-resistant tablets Naproxen 500mg Gastro-resistant tablet Oral
54463	Diclofenac 50mg Tablet (Approved Prescription Services Ltd) Diclofenac sodium 50mg Gastro-resistant tablet Oral
55099	Acoflam 100mg Retard tablets (Mercury Pharma Group Ltd) Diclofenac sodium 100mg Modified-release tablet Oral
77392	Motifene 75mg modified-release capsules (Lexon (UK) Ltd) Diclofenac sodium 75mg Modified-release capsule Oral
30849	Valdic 75 Retard tablets (Fannin UK Ltd) Diclofenac sodium 75mg Modified-release tablet Oral
2363	Dolobid 250mg tablets (Merck Sharp & Dohme Ltd) Diflunisal 250mg Tablet Oral
59203	Brufen 100mg/5ml syrup (Sigma Pharmaceuticals Plc) Ibuprofen 20mg/1ml Oral suspension Oral
34550	Ibuprofen 400mg tablets film coated (Actavis UK Ltd) Ibuprofen 400mg Tablet Oral
34924	Mefenamic acid 250mg Capsule (Teva UK Ltd) Mefenamic acid 250mg Capsule Oral

50652	Junior Ibuprofen 100mg/5ml oral suspension (Numark Ltd) Ibuprofen 20mg/1ml Oral suspension Oral
71376	Oruvail 100 modified-release capsules (Mawdsley-Brooks & Company Ltd) Ketoprofen 100mg Modified-release capsule Oral
30168	Arthroxen 250mg Tablet (C P Pharmaceuticals Ltd) Naproxen 250mg Tablet Oral
48568	Boots Rapid Ibuprofen lysine 342mg tablets (The Boots Company Plc) Ibuprofen lysine 200mg Tablet Oral
17733	Condrotec 500mg+200microgram Tablet (Pharmacia Ltd) Naproxen/Misoprostol 500mg+200microgram Tablet Oral
33457	Isclofen 50mg Gastro-resistant tablet (Isis Products Ltd) Diclofenac sodium 50mg Gastro-resistant tablet Oral
32509	Anadin Ibuprofen 200mg tablets (Pfizer Consumer Healthcare Ltd) Ibuprofen 200mg Tablet Oral
51360	Naproxen 250mg tablets (Accord Healthcare Ltd) Naproxen 250mg Tablet Oral
64303	Diclofenac sodium 25mg gastro-resistant tablets (DE Pharmaceuticals) Diclofenac sodium 25mg Gastro-resistant tablet Oral
48326	Ibuprofen 100mg/5ml oral suspension sugar free Ibuprofen 20mg/1ml Oral suspension Oral
341	Feldene 10mg capsules (Pfizer Ltd) Piroxicam 10mg Capsule Oral
39693	Naproxen 200mg/5ml oral suspension Naproxen 40mg/1ml Oral suspension Oral
63079	Ibuprofen 400mg tablets (Waymade Healthcare Plc) Ibuprofen 400mg Tablet Oral
2235	Relifex 500mg tablets (Mylan) Nabumetone 500mg Tablet Oral
30982	Naproxen 500mg gastro-resistant tablets (Actavis UK Ltd) Naproxen 500mg Gastro-resistant tablet Oral
41615	Indometacin 50mg Capsule (Approved Prescription Services Ltd) Indometacin 50mg Capsule Oral
64521	Arcoxia 90mg tablets (Mawdsley-Brooks & Company Ltd) Etoricoxib 90mg Tablet Oral
44703	Piroxicam 10mg Capsule (Berk Pharmaceuticals Ltd) Piroxicam 10mg Capsule Oral
34725	Flurbiprofen 50mg Tablet (Bristol-Myers Squibb Pharmaceuticals Ltd) Flurbiprofen 50mg Tablet Oral
15732	Diclovol 50mg gastro-resistant tablets (Arun Pharmaceuticals Ltd) Diclofenac sodium 50mg Gastro-resistant tablet Oral
24122	Diclofenac sodium 50mg gastro-resistant tablets (Actavis UK Ltd) Diclofenac sodium 50mg Gastro-resistant tablet Oral
24356	Eccoxolac 300mg capsules (Meda Pharmaceuticals Ltd) Etodolac 300mg Capsule Oral
78629	Meloxicam 15mg tablets (Mylan) Meloxicam 15mg Tablet Oral
37002	Nurofen Express 200mg liquid capsules (Reckitt Benckiser Healthcare (UK) Ltd) Ibuprofen 200mg Capsule Oral
80088	Naproxen 250mg gastro-resistant tablets (Sovereign Medical Ltd) Naproxen 250mg Gastro-resistant tablet Oral
60510	Nurofen for Children Cold, Pain and Fever Orange Flavour 100mg/5ml oral suspension (Reckitt Benckiser Healthcare (UK) Ltd) Ibuprofen 20mg/1ml Oral suspension Oral
73743	Ibuprofen 400mg tablets (Sigma Pharmaceuticals Plc) Ibuprofen 400mg Tablet Oral

62251	Arcoxia 90mg tablets (Sigma Pharmaceuticals Plc) Etoricoxib 90mg Tablet Oral
45331	Ibuprofen 200mg Tablet (Co-Pharma Ltd) Ibuprofen 200mg Tablet Oral
76342	Ibuprofen 200mg tablets (Bristol Laboratories Ltd) Ibuprofen 200mg Tablet Oral
46844	Dicloflex 75mg SR tablets (Actavis UK Ltd) Diclofenac sodium 75mg Modified-release tablet Oral
25750	Rheuflex 250mg Tablet (Goldshield Pharmaceuticals Ltd) Naproxen 250mg Tablet Oral
69584	Diclofenac sodium 100mg modified-release tablets (Sigma Pharmaceuticals Plc) Diclofenac sodium 100mg Modified-release tablet Oral
34931	Ibuprofen 200mg Tablet (Regent Laboratories Ltd) Ibuprofen 200mg Tablet Oral
3935	Feldene 20 capsules (Pfizer Ltd) Piroxicam 20mg Capsule Oral
10917	Flamrase SR 100mg tablets (Teva UK Ltd) Diclofenac sodium 100mg Modified-release tablet Oral
35265	Nurofen for children 3 months to 9 years 100mg/5ml Oral suspension (Reckitt Benckiser Healthcare (UK) Ltd) Ibuprofen 100mg/5ml Oral Suspension Oral
32234	Mefenamic acid 500mg tablets (IVAX Pharmaceuticals UK Ltd) Mefenamic acid 500mg Tablet Oral
34190	Indometacin 75mg modified-release capsules (A A H Pharmaceuticals Ltd) Indometacin 75mg Modified-release capsule Oral
35711	Dicloflex 25mg gastro-resistant tablets (Teva UK Ltd) Diclofenac sodium 25mg Gastro-resistant tablet Oral
71968	Ibuprofen 400mg capsules (Ennogen Healthcare Ltd) Ibuprofen 400mg Capsule Oral
36597	Hedex Ibuprofen 200mg tablets (Omega Pharma Ltd) Ibuprofen 200mg Tablet Oral
71088	Arthrotec 50 gastro-resistant tablets (Mawdsley-Brooks & Company Ltd) Diclofenac sodium/Misoprostol 50mg + 200microgram Gastro-resistant tablet Oral
5266	Lodine sr 600mg Modified-release tablet (Shire Pharmaceuticals Ltd) Etodolac 600mg Modified-release tablet Oral
45216	Ibuprofen 400mg Tablet (C P Pharmaceuticals Ltd) Ibuprofen 400mg Tablet Oral
63036	Ibuprofen 400mg capsules (AM Distributions (Yorkshire) Ltd) Ibuprofen 400mg Capsule Oral
9222	Dicloflex 75mg SR tablets (Dexcel-Pharma Ltd) Diclofenac sodium 75mg Modified-release tablet Oral
31962	Ketpron XL 200mg capsules (Mercury Pharma Group Ltd) Ketoprofen 200mg Modified-release capsule Oral
3974	Tenoxicam 20mg tablets Tenoxicam 20mg Tablet Oral
53626	Naproxen 500mg gastro-resistant tablets (Alliance Healthcare (Distribution) Ltd) Naproxen 500mg Gastro-resistant tablet Oral
50785	Diclofenac sodium 50mg gastro-resistant tablets (Genesis Pharmaceuticals Ltd) Diclofenac sodium 50mg Gastro-resistant tablet Oral
21610	Rhumalgan CR 100 tablets (Sandoz Ltd) Diclofenac sodium 100mg Modified-release tablet Oral
66194	Care Ibuprofen for Children 100mg/5ml oral suspension (Thornton & Ross Ltd) Ibuprofen 20mg/1ml Oral suspension Oral
68097	Naproxen 250mg tablets (Crescent Pharma Ltd) Naproxen 250mg Tablet Oral
38332	Ibucalm 200mg tablets (Aspar Pharmaceuticals Ltd) Ibuprofen 200mg Tablet Oral

79307	Meloxicam 7.5mg tablets (Mawdsley-Brooks & Company Ltd) Meloxicam 7.5mg Tablet Oral
28764	Closteril 100mg Modified-release tablet (Pharmalife Healthcare Services Ltd) Diclofenac sodium 100mg Modified-release tablet Oral
5648	Ibuprofen 200mg orodispersible tablets sugar free Ibuprofen 200mg Orodispersible tablet Oral
75581	Ketoprofen cr 100mg Capsule (Bristol-Myers Squibb Pharmaceuticals Ltd) Ketoprofen 100mg Modified-release capsule Oral
1468	Ibuprofen 200mg Soluble tablet Ibuprofen 200mg Soluble Tablet Oral
37731	Nurofen Express 342mg caplets (Reckitt Benckiser Healthcare (UK) Ltd) Ibuprofen lysine 200mg Tablet Oral
18196	Orbifen for children 100mg/5ml Oral suspension (Orbis Consumer Products Ltd) Ibuprofen 100mg/5ml Oral Suspension Oral
39317	Naproxen 500mg tablets (Wockhardt UK Ltd) Naproxen 500mg Tablet Oral
25257	Advil 200mg tablets (Wyeth Consumer Healthcare) Ibuprofen 200mg Tablet Oral
38944	Froben SR 200mg capsules (Abbott Laboratories Ltd) Flurbiprofen 200mg Modified-release capsule Oral
29181	Dicloflex 75mg SR tablets (Almus Pharmaceuticals Ltd) Diclofenac sodium 75mg Modified-release tablet Oral
41521	Indometacin 25mg Capsule (Approved Prescription Services Ltd) Indometacin 25mg Capsule Oral
62892	Ibuprofen 200mg tablets (Wockhardt UK Ltd) Ibuprofen 200mg Tablet Oral
62840	Celebrex 100mg capsules (DE Pharmaceuticals) Celecoxib 100mg Capsule Oral
849	Ibumed 400mg Tablet (Medipharma Ltd) Ibuprofen 400mg Tablet Oral
5482	Sulindac 200mg tablets Sulindac 200mg Tablet Oral
74659	Piroxicam 20mg capsules (Approved Prescription Services Ltd) Piroxicam 20mg Capsule Oral
10295	Relifex 500mg/5ml oral suspension (Meda Pharmaceuticals Ltd) Nabumetone 100mg/1ml Oral suspension Oral
34980	Ibuprofen 200mg tablets sugar coated (Actavis UK Ltd) Ibuprofen 200mg Tablet Oral
20105	Dicloflex 25mg Gastro-resistant tablet (Ratiopharm UK Ltd) Diclofenac sodium 25mg Gastro-resistant tablet Oral
66123	Diclofenac sodium 100mg modified-release tablets (Ethigen Ltd) Diclofenac sodium 100mg Modified-release tablet Oral
628	Diclofenac potassium 25mg tablets Diclofenac potassium 25mg Tablet Oral
80442	Arcoxia 60mg tablets (DE Pharmaceuticals) Etoricoxib 60mg Tablet Oral
24531	Mobiflex 20mg Effervescent tablet (Roche Products Ltd) Tenoxicam 20mg Effervescent Tablet Oral
39109	Feldene Melt 20mg tablets (Pfizer Ltd) Piroxicam 20mg Orodispersible tablet Oral
32100	Ibuprofen 600mg tablets (A A H Pharmaceuticals Ltd) Ibuprofen 600mg Tablet Oral
54021	Voltarol Retard 100mg tablets (Sigma Pharmaceuticals Plc) Diclofenac sodium 100mg Modified-release tablet Oral
66993	Naproxen 125mg/5ml oral suspension sugar free Naproxen 25mg/1ml Oral suspension Oral
77421	Relifex 500mg tablets (Waymade Healthcare Plc) Nabumetone 500mg Tablet Oral

9736	Mefenamic acid 50mg/5ml oral suspension Mefenamic acid 10mg/1ml Oral suspension Oral
76073	Boots Period Pain Relief 250mg gastro-resistant tablets (The Boots Company Plc) Naproxen 250mg Gastro-resistant tablet Oral
76093	Boots Ibuprofen 6 Months Plus 100mg/5ml oral suspension strawberry (The Boots Company Plc) Ibuprofen 20mg/1ml Oral suspension Oral
52420	Celebrex 100mg capsules (Mawdsley-Brooks & Company Ltd) Celecoxib 100mg Capsule Oral
24320	Indolar 50mg Capsule (Lagap) Indometacin 50mg Capsule Oral
77553	Celebrex 200mg capsules (Sigma Pharmaceuticals Plc) Celecoxib 200mg Capsule Oral
27013	Tiloket 200mg Modified-release capsule (Tillomed Laboratories Ltd) Ketoprofen 200mg Modified-release capsule Oral
59553	Ibuprofen 200mg tablets (Alliance Healthcare (Distribution) Ltd) Ibuprofen 200mg Tablet Oral
392	Ibuprofen 200mg modified-release capsules Ibuprofen 200mg Modified-release capsule Oral
27362	Diclofenac 100mg Modified-release tablet (Actavis UK Ltd) Diclofenac sodium 100mg Modified-release tablet Oral
64103	Mefenamic acid 500mg tablets (Almus Pharmaceuticals Ltd) Mefenamic acid 500mg Tablet Oral
80017	Celecoxib 200mg capsules (Milpharm Ltd) Celecoxib 200mg Capsule Oral
48738	Nurofen for Children 100mg/5ml oral suspension orange (Reckitt Benckiser Healthcare (UK) Ltd) Ibuprofen 20mg/1ml Oral suspension Oral
63843	Naproxen 250mg tablets (Milpharm Ltd) Naproxen 250mg Tablet Oral
65016	Celecoxib 200mg capsules (Alliance Healthcare (Distribution) Ltd) Celecoxib 200mg Capsule Oral
6464	Arcoxia 60mg tablets (Merck Sharp & Dohme Ltd) Etoricoxib 60mg Tablet Oral
50628	Ibuprofen 400mg caplets (The Boots Company Plc) Ibuprofen 400mg Tablet Oral
77459	Ketoprofen 100mg modified-release capsules (Alliance Healthcare (Distribution) Ltd) Ketoprofen 100mg Modified-release capsule Oral
34889	Ibuprofen 400mg Tablet (Celltech Pharma Europe Ltd) Ibuprofen 400mg Tablet Oral
16225	Dexomon retard 100mg Modified-release tablet (Hillcross Pharmaceuticals Ltd) Diclofenac sodium 100mg Modified-release tablet Oral
28255	Naproxen 250mg tablets (Wockhardt UK Ltd) Naproxen 250mg Tablet Oral
120	Indocid 25mg capsules (Merck Sharp & Dohme Ltd) Indometacin 25mg Capsule Oral
23323	Prosaid 500mg Tablet (BHR Pharmaceuticals Ltd) Naproxen 500mg Tablet Oral
53164	Diclofenac sodium 25mg gastro-resistant tablets (Kent Pharmaceuticals Ltd) Diclofenac sodium 25mg Gastro-resistant tablet Oral
76595	Meloxicam 7.5mg/5ml oral suspension Meloxicam 1.5mg/1ml Oral suspension Oral
40394	Advil 400mg Tablet (Wyeth Consumer Healthcare) Ibuprofen 400mg Tablet Oral
41701	Ibuprofen 600mg tablets (Actavis UK Ltd) Ibuprofen 600mg Tablet Oral
24020	Valrox 250mg Tablet (Shire Pharmaceuticals Ltd) Naproxen 250mg Tablet Oral
40664	Oruvail 150 modified-release capsules (Sanofi) Ketoprofen 150mg Modified-release capsule Oral
51242	Naproxen 500mg tablets (Pfizer Ltd) Naproxen 500mg Tablet Oral
59709	Prontoderm solution (B.Braun Medical Ltd)

26095	Ibuprofen lysine 400mg tablets Ibuprofen lysine 400mg Tablet Oral
417	Diclofenac 50mg dispersible tablets sugar free Diclofenac sodium 50mg Dispersible tablet Oral
25800	Feverfen 100mg/5ml oral suspension (Wise Pharmaceuticals Ltd) Ibuprofen 20mg/1ml Oral suspension Oral
42003	Indometacin sr 75mg Capsule (Lagap) Indometacin 75mg Modified-release capsule Oral
32536	Diclofenac 25mg Tablet (Berk Pharmaceuticals Ltd) Diclofenac sodium 25mg Gastro-resistant tablet Oral
32136	Ibular 200mg Tablet (Lagap) Ibuprofen 200mg Tablet Oral
30382	Ibuprofen 200mg Tablet (C P Pharmaceuticals Ltd) Ibuprofen 200mg Tablet Oral
21816	Pranoxen continus 500mg Tablet (Napp Pharmaceuticals Ltd) Naproxen sodium 500mg Modified-release tablet Oral
807	Naproxen 500mg tablets Naproxen 500mg Tablet Oral
21840	Arthrosin 250 tablets (Ashbourne Pharmaceuticals Ltd) Naproxen 250mg Tablet Oral
4880	Diclofenac sodium 75mg gastro-resistant / Misoprostol 200microgram tablets Diclofenac sodium/Misoprostol 75mg + 200microgram Gastro-resistant tablet Oral
25329	Lofensaid 25mg gastro-resistant tablets (Opus Pharmaceuticals Ltd) Diclofenac sodium 25mg Gastro-resistant tablet Oral
34425	Ibuprofen 400mg Tablet (Family Health) Ibuprofen 400mg Tablet Oral
4368	Lodine 200mg Capsule (Shire Pharmaceuticals Ltd) Etodolac 200mg Capsule Oral
20230	Salsalate 500mg capsules Salsalate 500mg Capsules Oral
647	Ibuprofen 100mg/5ml oral suspension Ibuprofen 20mg/1ml Oral suspension Oral
67740	Ibuprofen 600mg tablets (Fannin UK Ltd) Ibuprofen 600mg Tablet Oral
59246	Naproxen 500mg tablets (Accord Healthcare Ltd) Naproxen 500mg Tablet Oral
41513	Ibuprofen 200mg tablets (IVAX Pharmaceuticals UK Ltd) Ibuprofen 200mg Tablet Oral
32242	Ibuprofen 400mg tablets (Sterwin Medicines) Ibuprofen 400mg Tablet Oral
34218	Diclofenac 25mg Gastro-resistant tablet (Pharmacia Ltd) Diclofenac sodium 25mg Gastro-resistant tablet Oral
27366	Naproxen 500mg gastro-resistant tablets (Teva UK Ltd) Naproxen 500mg Gastro-resistant tablet Oral
589	Voltarol 50mg dispersible tablets (Novartis Pharmaceuticals UK Ltd) Diclofenac sodium 50mg Dispersible tablet Oral
61235	Meloxicam 7.5mg tablets (Sigma Pharmaceuticals Plc) Meloxicam 7.5mg Tablet Oral
1692	Diclofenac sodium 50mg gastro-resistant / Misoprostol 200microgram tablets Diclofenac sodium/Misoprostol 50mg + 200microgram Gastro-resistant tablet Oral
52714	Etodolac 600mg modified-release tablets (Alliance Healthcare (Distribution) Ltd) Etodolac 600mg Modified-release tablet Oral
18234	Rheumacin LA 75mg capsules (Hillcross Pharmaceuticals Ltd) Indometacin 75mg Modified-release capsule Oral
60705	Meloxicam 15mg tablets (Niche Generics Ltd) Meloxicam 15mg Tablet Oral
37094	Cuprofen 200mg tablets (SSL International Plc) Ibuprofen 200mg Tablet Oral

58842	Misofen 75mg/200microgram gastro-resistant tablets (Morningside Healthcare Ltd) Diclofenac sodium/Misoprostol 75mg + 200microgram Gastro-resistant tablet Oral
4631	Voltarol 50mg gastro-resistant tablets (Novartis Pharmaceuticals UK Ltd) Diclofenac sodium 50mg Gastro-resistant tablet Oral
59289	Diclofenac sodium 100mg modified-release tablets (AM Distributions (Yorkshire) Ltd) Diclofenac sodium 100mg Modified-release tablet Oral
56275	Meloxicam 7.5mg tablets (Teva UK Ltd) Meloxicam 7.5mg Tablet Oral
37750	Piroxicam 20mg capsules (Mylan) Piroxicam 20mg Capsule Oral
4625	Voltarol 75mg SR tablets (Novartis Pharmaceuticals UK Ltd) Diclofenac sodium 75mg Modified-release tablet Oral
21846	Pirozip 20 capsules (Ashbourne Pharmaceuticals Ltd) Piroxicam 20mg Capsule Oral
21831	Dysman 250mg Capsule (Ashbourne Pharmaceuticals Ltd) Mefenamic acid 250mg Capsule Oral
1757	Choline Mg trisalicylate 500mg tablets Magnesium Trisilicate 500mg Tablets Oral
26205	Fenbuzip 300mg Capsule (Ashbourne Pharmaceuticals Ltd) Fenbufen 300mg Capsule Oral
30790	Dicloflex sr 75mg Tablet (Genus Pharmaceuticals Ltd) Diclofenac sodium 75mg Modified-release tablet Oral
71949	Flarin 200mg capsules (infirst Healthcare Ltd) Ibuprofen 200mg Capsule Oral
24682	Tenoxicam 20mg effervescent tablets Tenoxicam 20mg Effervescent Tablet Oral
30389	Contraflam 250mg Capsule (Berk Pharmaceuticals Ltd) Mefenamic acid 250mg Capsule Oral
58572	Diclofenac potassium 25mg tablets (A A H Pharmaceuticals Ltd) Diclofenac potassium 25mg Tablet Oral
447	Diclofenac sodium 75mg modified-release capsules Diclofenac sodium 75mg Modified-release capsule Oral
2863	Tiaprofenic acid 300mg tablets Tiaprofenic acid 300mg Tablet Oral
4309	Ibuprofen lysine 200mg tablets Ibuprofen lysine 200mg Tablet Oral
59595	Diclofenac 50mg dispersible tablets sugar free (Sigma Pharmaceuticals Plc) Diclofenac sodium 50mg Dispersible tablet Oral
68220	Ibuprofen 200mg capsules (Ennogen Healthcare Ltd) Ibuprofen 200mg Capsule Oral
39758	Nurofen Express 256mg caplets (Reckitt Benckiser Healthcare (UK) Ltd) Ibuprofen sodium dihydrate 200mg Tablet Oral
51828	Ibuprofen 100mg/5ml oral suspension sugar free (Kent Pharmaceuticals Ltd) Ibuprofen 20mg/1ml Oral suspension Oral
3852	Diclomax 100mg Modified-release capsule (Provalis Healthcare Ltd) Diclofenac sodium 100mg Modified-release capsule Oral
44986	Vimovo 500mg/20mg modified-release tablets (AstraZeneca UK Ltd) Naproxen/Esomeprazole magnesium trihydrate 500mg + 20mg Modified-release tablet Oral
24305	Ibufac 400mg Tablet (DDSA Pharmaceuticals Ltd) Ibuprofen 400mg Tablet Oral
67594	Ibuprofen 400mg tablets (Kent Pharmaceuticals Ltd) Ibuprofen 400mg Tablet Oral
29772	Ketotard XL 200mg capsules (Galen Ltd) Ketoprofen 200mg Modified-release capsule Oral
21387	Diclofenac sodium 50mg gastro-resistant tablets (Mylan) Diclofenac sodium 50mg Gastro-resistant tablet Oral

7524	Feldene 20mg dispersible tablets (Pfizer Ltd) Piroxicam 20mg Dispersible tablet Oral
7434	Clinoril 100mg tablets (Merck Sharp & Dohme Ltd) Sulindac 100mg Tablet Oral
34793	Mefenamic acid 250mg capsules (Zentiva) Mefenamic acid 250mg Capsule Oral
34922	Naproxen 500mg Tablet (Berk Pharmaceuticals Ltd) Naproxen 500mg Tablet Oral
40336	Orudis 50mg capsules (Sanofi) Ketoprofen 50mg Capsule Oral
75677	Ibuprofen 400mg tablets (Phoenix Healthcare Distribution Ltd) Ibuprofen 400mg Tablet Oral
41524	Mefenamic acid 500mg tablets (Teva UK Ltd) Mefenamic acid 500mg Tablet Oral
56584	Arcoxia 60mg tablets (Lexon (UK) Ltd) Etoricoxib 60mg Tablet Oral
29749	Ibuprofen 200mg tablets (Ranbaxy (UK) Ltd) Ibuprofen 200mg Tablet Oral
32365	Relcofen 400mg tablets (Actavis UK Ltd) Ibuprofen 400mg Tablet Oral
30806	Rhumalgan 50mg Tablet (Lagap) Diclofenac sodium 50mg Gastro-resistant tablet Oral
47994	Naproxen 250mg Gastro-resistant tablet (Almus Pharmaceuticals Ltd) Naproxen 250mg Gastro-resistant tablet Oral
60368	Diclofenac 10mg/5ml oral solution Diclofenac sodium 2mg/1ml Oral solution Oral
58415	Diclofenac sodium 50mg gastro-resistant / Misoprostol 200microgram tablets (A A H Pharmaceuticals Ltd) Diclofenac sodium/Misoprostol 50mg + 200microgram Gastro-resistant tablet Oral
53397	Brufen 100mg/5ml syrup (Mawdsley-Brooks & Company Ltd) Ibuprofen 20mg/1ml Oral suspension Oral
22206	Nurofen Long Lasting 300mg capsules (Crookes Healthcare Ltd) Ibuprofen 300mg Modified-release capsule Oral
46921	Ibuprofen 400mg tablets (Ranbaxy (UK) Ltd) Ibuprofen 400mg Tablet Oral
73981	Feldene Melt 20mg tablets (Sigma Pharmaceuticals Plc) Piroxicam 20mg Orodispersible tablet Oral
1030	Junifen 100mg/5ml Oral suspension (Crookes Healthcare Ltd) Ibuprofen 100mg/5ml Oral Suspension Oral
7426	Lederfen 300mg Capsule (Wyeth Pharmaceuticals) Fenbufen 300mg Capsule Oral
17525	Fenactol Retard 100mg tablets (Dexcel-Pharma Ltd) Diclofenac sodium 100mg Modified-release tablet Oral
40516	Anadin Ultra 200mg capsules (Pfizer Consumer Healthcare Ltd) Ibuprofen 200mg Capsule Oral
60930	Indometacin 25mg capsules (Alliance Healthcare (Distribution) Ltd) Indometacin 25mg Capsule Oral
3431	Naproxen 250mg gastro-resistant tablets Naproxen 250mg Gastro-resistant tablet Oral
77694	Piroxicam 10mg capsules (Approved Prescription Services Ltd) Piroxicam 10mg Capsule Oral
74806	Brufen Retard 800mg tablets (Dowelhurst Ltd) Ibuprofen 800mg Modified-release tablet Oral
38881	Diclomax SR 75mg capsules (Galen Ltd) Diclofenac sodium 75mg Modified-release capsule Oral
80679	Naproxen 50mg/ml oral suspension (DE Pharmaceuticals) Naproxen 50mg/1ml Oral suspension Oral

75305 Galprofen Long Lasting 300mg capsules (Galpharm International Ltd) Ibuprofen 300mg Modified-release capsule Oral

24308 Slo-Indo 75mg capsules (Mylan) Indometacin 75mg Modified-release capsule Oral

20621 Dicloflex 75mg SR tablets (Kent Pharmaceuticals Ltd) Diclofenac sodium 75mg Modified-release tablet Oral

34738 Naproxen 250mg gastro-resistant tablets (A A H Pharmaceuticals Ltd) Naproxen 250mg Gastro-resistant tablet Oral

11495 Piroxicam betadex 20mg tablets Piroxicam betadex 20mg Tablet Oral

50314 Brufen 600mg effervescent granules sachets (DE Pharmaceuticals) Ibuprofen 600mg Effervescent granules Oral

20978 Anadin Ultra liquid capsules (Wyeth Consumer Healthcare) Ibuprofen 200mg Capsule Oral

4806 Voltarol 100mg suppositories (Novartis Pharmaceuticals UK Ltd) Diclofenac sodium 100mg Suppository Rectal

65528 Arthrotec 75 gastro-resistant tablets (Lexon (UK) Ltd) Diclofenac sodium/Misoprostol 75mg + 200microgram Gastro-resistant tablet Oral

917 Diclofenac sodium 50mg tablets Diclofenac Sodium 50mg Tablets Oral

34977 Naproxen 500mg Gastro-resistant tablet (Galen Ltd) Naproxen 500mg Gastro-resistant tablet Oral

14672 Defanac 75mg SR tablets (Ranbaxy (UK) Ltd) Diclofenac sodium 75mg Modified-release tablet Oral

80671 Celecoxib 100mg capsules (Brown & Burk UK Ltd) Celecoxib 100mg Capsule Oral

28256 Diclofenac 50mg Tablet (Berk Pharmaceuticals Ltd) Diclofenac sodium 50mg Gastro-resistant tablet Oral

75136 Diclofenac 25mg Tablet (C P Pharmaceuticals Ltd) Diclofenac sodium 25mg Gastro-resistant tablet Oral

53980 Naproxen 250mg tablets (Phoenix Healthcare Distribution Ltd) Naproxen 250mg Tablet Oral

45256 Indometacin 25mg Capsule (Meridian Healthcare (UK) Ltd) Indometacin 25mg Capsule Oral

3416 Diclofenac sodium 100mg modified-release tablets Diclofenac sodium 100mg Modified-release tablet Oral

40086 Acoflam 50mg gastro-resistant tablets (Mercury Pharma Group Ltd) Diclofenac sodium 50mg Gastro-resistant tablet Oral

71584 Boots Ibuprofen 3 Months Plus 100mg/5ml oral suspension strawberry (The Boots Company Plc) Ibuprofen 20mg/1ml Oral suspension Oral

67803 Oruvail 200 modified-release capsules (Lexon (UK) Ltd) Ketoprofen 200mg Modified-release capsule Oral

46342 Medifen 3with months 100mg/5ml Oral suspension (SSL International Plc) Ibuprofen 100mg/5ml Oral Suspension Oral

39085 Naproxen 250mg tablets (A A H Pharmaceuticals Ltd) Naproxen 250mg Tablet Oral

156 Diclofenac diethylammonium 1.16% gel Diclofenac diethylammonium 11.6mg/1gram Gel Transdermal

17124 Dicloflex sr 100mg Tablet (IVAX Pharmaceuticals UK Ltd) Diclofenac sodium 100mg Modified-release tablet Oral

29316 Care ibuprofen 400mg Tablet (Thornton & Ross Ltd) Ibuprofen 400mg Tablet Oral

Diclofenac potassium 50mg tablets (Accord Healthcare Ltd) Diclofenac potassium
43045 50mg Tablet Oral

34729 Ibuprofen 400mg tablets (OBG Pharmaceuticals Ltd) Ibuprofen 400mg Tablet Oral
416 Ibuprofen 200mg tablets Ibuprofen 200mg Tablet Oral

45842 Ibuprofen 600mg Tablet (Celltech Pharma Europe Ltd) Ibuprofen 600mg Tablet Oral
37587 Etoricoxib 30mg tablets Etoricoxib 30mg Tablet Oral

76191 Meloxicam 7.5mg tablets (A A H Pharmaceuticals Ltd) Meloxicam 7.5mg Tablet Oral
34757 Ibuprofen 400mg Tablet (Unichem) Ibuprofen 400mg Tablet Oral
Butazolidin 200mg Tablet (Novartis Pharmaceuticals UK Ltd) Phenylbutazone 200mg
29674 Tablet Oral
Flamatak MR 75mg tablets (Actavis UK Ltd) Diclofenac sodium 75mg Modified-
20395 release tablet Oral
Dicloflex Retard 100mg tablets (Dexcel-Pharma Ltd) Diclofenac sodium 100mg
39264 Modified-release tablet Oral
27723 Phenylbutazone 200mg tablets Phenylbutazone 200mg Tablet Oral
Naproxen 375mg Modified-release tablet Naproxen 375mg Modified-Release Tablet
15023 Oral
Apsifen 400mg Tablet (Approved Prescription Services Ltd) Ibuprofen 400mg Tablet
27968 Oral
69266 Fusidic acid 2% cream (Teva UK Ltd) Fusidic acid 20mg/1gram Cream Cutaneous
Orbifen For Children 100mg/5ml oral suspension (Orbis Consumer Products Ltd)
51943 Ibuprofen 20mg/1ml Oral suspension Oral
Diclofenac 12.5mg/5ml oral suspension Diclofenac sodium 2.5mg/1ml Oral
68849 suspension Oral
Mefenamic acid 250mg capsules (A A H Pharmaceuticals Ltd) Mefenamic acid 250mg
34438 Capsule Oral
Naproxen 250mg Gastro-resistant tablet (Galen Ltd) Naproxen 250mg Gastro-
34670 resistant tablet Oral
72972 Indometacin Oral solution Indometacin Oral Solution

37553 Ibucalm 400mg tablets (Aspar Pharmaceuticals Ltd) Ibuprofen 400mg Tablet Oral
Rhumalgan XL 100mg capsules (Sandoz Ltd) Diclofenac sodium 100mg Modified-
26631 release capsule Oral
Galprofen Long Lasting 200mg capsules (Galpharm International Ltd) Ibuprofen
28888 200mg Modified-release capsule Oral
Apsifen 200mg Tablet (Approved Prescription Services Ltd) Ibuprofen 200mg Tablet
31482 Oral

28695 Piroflam 10mg Capsule (Opus Pharmaceuticals Ltd) Piroxicam 10mg Capsule Oral
Clotam 200mg Capsule (Thames Laboratories Ltd) Tolfenamic Acid 200mg Capsule
20036 Oral

41622 Piroxicam 10mg capsules (A A H Pharmaceuticals Ltd) Piroxicam 10mg Capsule Oral
Naproxen 250mg gastro-resistant tablets (Sigma Pharmaceuticals Plc) Naproxen
67363 250mg Gastro-resistant tablet Oral
Mefenamic acid 500mg tablets (Waymade Healthcare Plc) Mefenamic acid 500mg
66452 Tablet Oral

21123	Piroxicam 20mg Capsule (Berk Pharmaceuticals Ltd) Piroxicam 20mg Capsule Oral
25361	Diclovol 25mg gastro-resistant tablets (Arun Pharmaceuticals Ltd) Diclofenac sodium 25mg Gastro-resistant tablet Oral
4965	Piroxicam 20mg orodispersible tablets sugar free Piroxicam 20mg Orodispersible tablet Oral
58213	Naproxen 500mg tablets (Milpharm Ltd) Naproxen 500mg Tablet Oral
46942	Ibuprofen 600mg tablets (IVAX Pharmaceuticals UK Ltd) Ibuprofen 600mg Tablet Oral
13893	Nurofen Plus tablets (Reckitt Benckiser Healthcare (UK) Ltd) Ibuprofen/Codeine phosphate 200mg + 12.8mg Tablet Oral
48138	Ibuprofen 200mg tablets (Aspar Pharmaceuticals Ltd) Ibuprofen 200mg Tablet Oral
17165	Nycopren 500mg gastro-resistant tablets (Ardern Healthcare Ltd) Naproxen 500mg Gastro-resistant tablet Oral
58523	Indometacin 50mg capsules (Almus Pharmaceuticals Ltd) Indometacin 50mg Capsule Oral
78978	Ibuprofen Seven Plus Pain Relief 200mg/5ml oral suspension (Aspire Pharma Ltd) Ibuprofen 40mg/1ml Oral suspension Oral
34923	Naproxen 250mg Tablet (Berk Pharmaceuticals Ltd) Naproxen 250mg Tablet Oral
32641	Indometacin 25mg capsules (A A H Pharmaceuticals Ltd) Indometacin 25mg Capsule Oral
54518	Diclofenac sodium 50mg gastro-resistant tablets (Phoenix Healthcare Distribution Ltd) Diclofenac sodium 50mg Gastro-resistant tablet Oral
27082	Ketpron XL 100mg capsules (Mercury Pharma Group Ltd) Ketoprofen 100mg Modified-release capsule Oral
25330	Solpaflex tablets (GlaxoSmithKline Consumer Healthcare) Ibuprofen/Codeine phosphate 200mg + 12.8mg Tablet Oral
41677	Mefenamic acid 250mg Capsule (IVAX Pharmaceuticals UK Ltd) Mefenamic acid 250mg Capsule Oral
1571	Ketoprofen 100mg modified-release capsules Ketoprofen 100mg Modified-release capsule Oral
58708	Naproxen 500mg gastro-resistant tablets (Ranbaxy (UK) Ltd) Naproxen 500mg Gastro-resistant tablet Oral
35890	Nurofen 200mg caplets (Reckitt Benckiser Healthcare (UK) Ltd) Ibuprofen 200mg Tablet Oral
51829	Naproxen 250mg tablets (Kent Pharmaceuticals Ltd) Naproxen 250mg Tablet Oral
18364	Ibular 400mg Tablet (Lagap) Ibuprofen 400mg Tablet Oral
76869	Naproxen 500mg gastro-resistant tablets (Sigma Pharmaceuticals Plc) Naproxen 500mg Gastro-resistant tablet Oral
60408	Naproxen 500mg gastro-resistant tablets (Waymade Healthcare Plc) Naproxen 500mg Gastro-resistant tablet Oral
61878	Nurofen Express Period Pain 200mg capsules (Reckitt Benckiser Healthcare (UK) Ltd) Ibuprofen 200mg Capsule Oral
65952	Naproxen 500mg gastro-resistant tablets (Almus Pharmaceuticals Ltd) Naproxen 500mg Gastro-resistant tablet Oral
77680	Naproxen 500mg gastro-resistant tablets (IVAX Pharmaceuticals UK Ltd) Naproxen 500mg Gastro-resistant tablet Oral

25643	Surgam 300mg Sachets (Sanofi) Tiaprofenic Acid 300mg Sachets Oral
1766	Voltarol sr 75mg Modified-release tablet (Novartis Pharmaceuticals UK Ltd) Diclofenac sodium 75mg Modified-release tablet Oral
2234	Nabumetone 500mg tablets Nabumetone 500mg Tablet Oral
74211	Diclofenac sodium 50mg gastro-resistant tablets (DE Pharmaceuticals) Diclofenac sodium 50mg Gastro-resistant tablet Oral
78985	Naproxen 125mg/5ml oral suspension sugar free (A A H Pharmaceuticals Ltd) Naproxen 25mg/1ml Oral suspension Oral
2904	Diclofenac sodium 75mg gastro-resistant modified-release capsules Diclofenac sodium 75mg Modified-release capsule Oral
407	Brufen 600mg effervescent granules sachets (Mylan) Ibuprofen 600mg Effervescent granules Oral
32916	Diclofenac 75mg Modified-release capsule (Sandoz Ltd) Diclofenac sodium 75mg Modified-release capsule Oral
24007	Valrox 500mg Tablet (Shire Pharmaceuticals Ltd) Naproxen 500mg Tablet Oral
56925	Naproxen 250mg tablets (Actavis UK Ltd) Naproxen 250mg Tablet Oral
34091	Diclofenac sodium 25mg gastro-resistant tablets (Sandoz Ltd) Diclofenac sodium 25mg Gastro-resistant tablet Oral
46940	Ketoprofen 100mg capsules (Mylan) Ketoprofen 100mg Capsule Oral
17750	Indomax 25mg Capsule (Ashbourne Pharmaceuticals Ltd) Indometacin 25mg Capsule Oral
9637	Keral 25mg tablets (A. Menarini Farmaceutica Internazionale SRL) Dexketoprofen trometamol 25mg Tablet Oral
27783	Ibuprofen 400mg tablets sugar coated (Actavis UK Ltd) Ibuprofen 400mg Tablet Oral
70145	Voltarol Rapid 50mg tablets (Stephar (U.K.) Ltd) Diclofenac potassium 50mg Tablet Oral
80366	Ibuprofen 200mg tablets (Phoenix Healthcare Distribution Ltd) Ibuprofen 200mg Tablet Oral
10325	Dexibuprofen 300mg tablets Dexibuprofen 300mg Tablet Oral
10678	Fenopron 300 tablets (Typharm Ltd) Fenoprofen calcium 300mg Tablet Oral
5455	Etodolac 600mg modified-release tablets Etodolac 600mg Modified-release tablet Oral
7483	Butazolidin 100mg Tablet (Novartis Pharmaceuticals UK Ltd) Phenylbutazone 100mg Gastro-resistant tablet Oral
42905	Diclofenac 75mg Modified-release tablet (Actavis UK Ltd) Diclofenac sodium 75mg Modified-release tablet Oral
40	Diclofenac sodium 50mg gastro-resistant tablets Diclofenac sodium 50mg Gastro-resistant tablet Oral
51284	Arcoxia 60mg tablets (Sigma Pharmaceuticals Plc) Etoricoxib 60mg Tablet Oral
68708	Indometacin 75mg modified-release capsules (DE Pharmaceuticals) Indometacin 75mg Modified-release capsule Oral
70828	Naproxen 500mg tablets (DE Pharmaceuticals) Naproxen 500mg Tablet Oral
37053	Migrafen 200mg tablets (Chatfield Laboratories) Ibuprofen 200mg Tablet Oral
5173	Dexketoprofen 25mg tablets Dexketoprofen trometamol 25mg Tablet Oral
72047	Naproxen 250mg gastro-resistant tablets (Phoenix Healthcare Distribution Ltd) Naproxen 250mg Gastro-resistant tablet Oral
71779	Boots Ibuprofen Long Lasting 200mg capsules (The Boots Company Plc) Ibuprofen 200mg Modified-release capsule Oral

25701	Ketovail 200mg modified-release capsules (Teva UK Ltd) Ketoprofen 200mg Modified-release capsule Oral
11550	Nurofen Meltlets 200mg tablets (Reckitt Benckiser Healthcare (UK) Ltd) Ibuprofen 200mg Orodispersible tablet Oral
16221	Diclozip 25mg gastro-resistant tablets (Ashbourne Pharmaceuticals Ltd) Diclofenac sodium 25mg Gastro-resistant tablet Oral
15104	Naproxen 500mg Granules Naproxen 500mg Granules Oral
30892	Mandafen for Children 100mg/5ml oral suspension sugar free (M & A Pharmachem Ltd) Ibuprofen 20mg/1ml Oral suspension Oral
65877	Diclofenac sodium 75mg modified-release tablets (Mawdsley-Brooks & Company Ltd) Diclofenac sodium 75mg Modified-release tablet Oral
15201	Volraman 50mg gastro-resistant tablets (LPC Medical (UK) Ltd) Diclofenac sodium 50mg Gastro-resistant tablet Oral
9465	Diclotard 100 100mg Modified-release tablet (Galen Ltd) Diclofenac sodium 100mg Modified-release tablet Oral
7535	Nurofen 200mg Capsule (Crookes Healthcare Ltd) Ibuprofen 200mg Capsule Oral
70904	Indometacin 25mg capsules (Crescent Pharma Ltd) Indometacin 25mg Capsule Oral
29524	Ibumetin 600mg Tablet (Alfred Benzon (UK) Ltd) Ibuprofen 600mg Tablet Oral
23121	Arthroxen 500mg Tablet (C P Pharmaceuticals Ltd) Naproxen 500mg Tablet Oral
40083	Ibuprofen 200mg caplets (Galpharm International Ltd) Ibuprofen 200mg Tablet Oral
7058	Calprofen 100mg/5ml Oral suspension (McNeil Products Ltd) Ibuprofen 100mg/5ml Oral Suspension Oral
1139	Voltarol 25mg Tablet (Novartis Pharmaceuticals UK Ltd) Diclofenac sodium 25mg Gastro-resistant tablet Oral
10033	Etodolac 300mg capsules Etodolac 300mg Capsule Oral
54304	Naproxen 500mg tablets (Actavis UK Ltd) Naproxen 500mg Tablet Oral
19007	Naprosyn 500mg Granules (Roche Products Ltd) Naproxen 500mg Granules Oral
38992	Flamrase 75mg SR tablets (Teva UK Ltd) Diclofenac sodium 75mg Modified-release tablet Oral
34359	Ibuprofen 400mg tablets (Vantage) Ibuprofen 400mg Tablet Oral
39354	Galpharm ibuprofen for children 100mg/5ml Oral suspension (Galpharm International Ltd) Ibuprofen 100mg/5ml Oral Suspension Oral
29455	Flexotard MR 100mg tablets (Pfizer Ltd) Diclofenac sodium 100mg Modified-release tablet Oral
60786	Voltarol 50mg dispersible tablets (Sigma Pharmaceuticals Plc) Diclofenac sodium 50mg Dispersible tablet Oral
71709	Naproxen 50mg/ml oral suspension (A A H Pharmaceuticals Ltd) Naproxen 50mg/1ml Oral suspension Oral
49133	Nurofen for Children 100mg/5ml oral suspension strawberry (Reckitt Benckiser Healthcare (UK) Ltd) Ibuprofen 20mg/1ml Oral suspension Oral
560	Diflunisal 250mg tablets Diflunisal 250mg Tablet Oral
71307	Diclofenac sodium 100mg modified-release capsules (DE Pharmaceuticals) Diclofenac sodium 100mg Modified-release capsule Oral
126	Ponstan 250mg capsules (Chemidex Pharma Ltd) Mefenamic acid 250mg Capsule Oral
1688	Indocid 50mg capsules (Merck Sharp & Dohme Ltd) Indometacin 50mg Capsule Oral

30391	Contraflam 500mg Tablet (Berk Pharmaceuticals Ltd) Mefenamic acid 500mg Tablet Oral
39873	Cuprofen Maximum Strength 400mg tablets (Reckitt Benckiser Healthcare (UK) Ltd) Ibuprofen 400mg Tablet Oral
75569	Mefenamic acid 250mg capsules (Waymade Healthcare Plc) Mefenamic acid 250mg Capsule Oral
33357	Pacifene 200mg tablets (Sussex Pharmaceutical Ltd) Ibuprofen 200mg Tablet Oral
1469	Meloxicam 15mg tablets Meloxicam 15mg Tablet Oral
38493	Anadin Joint Pain 200mg tablets (Pfizer Consumer Healthcare Ltd) Ibuprofen 200mg Tablet Oral
11168	Volsaid Retard 100 tablets (Chiesi Ltd) Diclofenac sodium 100mg Modified-release tablet Oral
61581	Mefenamic acid 500mg tablets (Essential Generics Ltd) Mefenamic acid 500mg Tablet Oral
77951	Naproxen 250mg tablets (Mawdsley-Brooks & Company Ltd) Naproxen 250mg Tablet Oral
70221	Mefenamic acid 250mg capsules (Alliance Healthcare (Distribution) Ltd) Mefenamic acid 250mg Capsule Oral
48062	Ibuprofen 200mg Tablet (Wockhardt UK Ltd) Ibuprofen 200mg Tablet Oral
75095	Naproxen 250mg gastro-resistant tablets (DE Pharmaceuticals) Naproxen 250mg Gastro-resistant tablet Oral
21419	Seractil 300mg tablets (Thornton & Ross Ltd) Dexibuprofen 300mg Tablet Oral
16222	Diclozip 50mg gastro-resistant tablets (Ashbourne Pharmaceuticals Ltd) Diclofenac sodium 50mg Gastro-resistant tablet Oral
20385	Arthrosin 500 tablets (Ashbourne Pharmaceuticals Ltd) Naproxen 500mg Tablet Oral
59878	Naproxen 250mg gastro-resistant tablets (Almus Pharmaceuticals Ltd) Naproxen 250mg Gastro-resistant tablet Oral
73156	Nimesulide (roi) 100mg Tablet Nimesulide 100mg Tablet
9688	Diclovol 75mg SR tablets (Mylan) Diclofenac sodium 75mg Modified-release tablet Oral
33308	Butazone 100mg Tablet (DDSA Pharmaceuticals Ltd) Phenylbutazone 100mg Gastro-resistant tablet Oral
77359	Indocid R 75mg capsules (Dowelhurst Ltd) Indometacin 75mg Modified-release capsule Oral
55313	Ibuprofen 400mg tablets (Boston Healthcare Ltd) Ibuprofen 400mg Tablet Oral
15286	Ketocid 200 modified-release capsules (Chiesi Ltd) Ketoprofen 200mg Modified-release capsule Oral
30811	Proflex 300mg Modified-release capsule (Novartis Consumer Health UK Ltd) Ibuprofen 300mg Modified-release capsule Oral
6498	Arcoxia 90mg tablets (Merck Sharp & Dohme Ltd) Etoricoxib 90mg Tablet Oral
39461	Solpadeine Migraine Ibuprofen & Codeine tablets (Omega Pharma Ltd) Ibuprofen/Codeine phosphate 200mg + 12.8mg Tablet Oral
73433	Ibuprofen 600mg tablets (Almus Pharmaceuticals Ltd) Ibuprofen 600mg Tablet Oral
40253	Ibuprofen 600mg Tablet (Sovereign Medical Ltd) Ibuprofen 600mg Tablet Oral
54075	Voltarol 50mg dispersible tablets (Stephar (U.K.) Ltd) Diclofenac sodium 50mg Dispersible tablet Oral
8451	Etodolac 200mg Tablet Etodolac 200mg Tablet Oral

4320	Naprosyn 125mg/5ml oral suspension (Roche Products Ltd) Naproxen 25mg/1ml Oral suspension Oral
649	Diclofenac sodium 25mg gastro-resistant tablets Diclofenac sodium 25mg Gastro-resistant tablet Oral
60115	Naproxen 250mg tablets (Alliance Healthcare (Distribution) Ltd) Naproxen 250mg Tablet Oral
80882	Diclofenac 15mg/5ml oral suspensionDiclofenac sodium3mg/1mlOral suspensionOral
81056	Naproxen 500mg gastro-resistant tablets (Medreich Plc)Naproxen500mgGastro-resistant tabletOral
81125	Ibuprofen 100mg/5ml oral suspension sugar free (Actavis UK Ltd)Ibuprofen20mg/1mlOral suspensionOral
81403	Celecoxib 200mg capsules (Brown & Burk UK Ltd)Celecoxib200mgCapsuleOral
81441	Naproxen 500mg tablets (Almus Pharmaceuticals Ltd)Naproxen500mgTabletOral
81636	Ibuprofen 200mg tablets (Sussex Pharmaceutical Ltd)Ibuprofen200mgTabletOral
81644	Masidemen 75mg/200microgram gastro-resistant tablets (Actavis UK Ltd)Diclofenac sodium/Misoprostol75mg + 200microgramGastro-resistant tabletOral
81720	Etoricoxib 90mg tablets (Mylan)Etoricoxib90mgTabletOral
81765	Naproxen 375mg gastro-resistant tablets (Actavis UK Ltd)Naproxen375mgGastro-resistant tabletOral
81769	Naproxen 375mg gastro-resistant tablets (Alliance Healthcare (Distribution) Ltd)Naproxen375mgGastro-resistant tabletOral
81770	Naproxen 375mg gastro-resistant tablets (DE Pharmaceuticals)Naproxen375mgGastro-resistant tabletOral
81771	Naproxen 375mg gastro-resistant tablets (A A H Pharmaceuticals Ltd)Naproxen375mgGastro-resistant tabletOral
81801	Diclo-SR 75mg tablets (Strides Pharma UK Ltd)Diclofenac sodium75mgModified-release tabletOral
81837	Arcoxia 90mg tablets (CST Pharma Ltd)Etoricoxib90mgTabletOral
82104	Naproxen 500mg tablets (Bristol Laboratories Ltd)Naproxen500mgTabletOral
82137	Ibuprofen 400mg tablets (Wockhardt UK Ltd)Ibuprofen400mgTabletOral
82181	Etoricoxib 60mg tablets (Mylan)Etoricoxib60mgTabletOral
82301	Arcoxia 90mg tablets (Necessity Supplies Ltd)Etoricoxib90mgTabletOral
82347	Naproxen 375mg gastro-resistant tablets (Sigma Pharmaceuticals Plc)Naproxen375mgGastro-resistant tabletOral
82354	Feldene Melt 20mg tablets (Lexon (UK) Ltd)Piroxicam20mgOrodispersible tabletOral
82373	Etodolac 600mg modified-release tablets (Zentiva)Etodolac600mgModified-release tabletOral
82426	Arcoxia 30mg tablets (Waymade Healthcare Plc)Etoricoxib30mgTabletOral
82428	Arcoxia 120mg tablets (Lexon (UK) Ltd)Etoricoxib120mgTabletOral
82503	Brufen Retard 800mg tablets (Waymade Healthcare Plc)Ibuprofen800mgModified-release tabletOral
82517	Voltarol 75mg SR tablets (Dowelhurst Ltd)Diclofenac sodium75mgModified-release tabletOral
82550	Arthrotec 75 gastro-resistant tablets (Waymade Healthcare Plc)Diclofenac sodium/Misoprostol75mg + 200microgramGastro-resistant tabletOral

82690	Diclofenac potassium 50mg tablets (Medihealth (Northern) Ltd)Diclofenac potassium50mgTabletOral
82721	Flurbiprofen 100mg tablets (Mylan)Flurbiprofen100mgTabletOral
82737	Diclofenac sodium 25mg capsulesDiclofenac Sodium
82815	Ibuprofen 200mg tablets (Noumed Life Sciences Ltd)Ibuprofen200mgTabletOral

Colchicine

**DRUG_CODE**	DESCRIPTION
8643	COLCHICINE .5 MG TAB
12086	COLCHICINE 250 MCG CAP
77032	Colchicine 500microgram tablets (Teva UK Ltd) Colchicine 500microgram Tablet Oral
76421	Colchicine 400micrograms/5ml oral suspension Colchicine 80microgram/1ml Oral suspension Oral
34469	Colchicine 500microgram tablets (Wockhardt UK Ltd) Colchicine 500microgram Tablet Oral
34329	Colchicine 500microgram tablets (UCB Pharma Ltd) Colchicine 500microgram Tablet Oral
66679	PROBENECID/COLCHICINE 500 MG TAB
71853	Colchicine 500microgram tablets (DE Pharmaceuticals) Colchicine 500microgram Tablet Oral
762	Colchicine 500microgram tablets Colchicine 500microgram Tablet Oral
46962	Colchicine 500microgram tablets (A A H Pharmaceuticals Ltd) Colchicine 500microgram Tablet Oral
357	COLCHICINE 250 MCG TAB
66469	Colchicine 500microgram tablets (Alliance Healthcare (Distribution) Ltd) Colchicine 500microgram Tablet Oral
29961	COLCHICUM & SODIUM SALICYLATE MIX
71052	Colchicine 500microgram tablets (Kent Pharmaceuticals Ltd) Colchicine 500microgram Tablet Oral
21063	COLCHICINE .1 MG TAB
80866	COLCHICINE 300 MCG TAB

Urate lowering therapy

DRUG_CODE_	DESCRIPTION
34005	Allopurinol 300mg tablets (Teva UK Ltd) Allopurinol 300mg Tablet Oral
41612	Allopurinol 300mg tablets (Actavis UK Ltd) Allopurinol 300mg Tablet Oral
54139	Allopurinol 300mg/5ml oral suspension Allopurinol 60mg/1ml Oral suspension Oral
23368	Hamarin 100 Tablet (Nicholas Laboratories Ltd) Allopurinol 100mg Tablet Oral
46941	Allopurinol 300mg Tablet (Lagap) Allopurinol 300mg Tablet Oral
44239	Cosuric 100mg Tablet (DDSA Pharmaceuticals Ltd) Allopurinol 100mg Tablet Oral
368	Zyloric 100mg tablets (Aspen Pharma Trading Ltd) Allopurinol 100mg Tablet Oral
67347	Allopurinol 300mg/5ml oral solution Allopurinol 60mg/1ml Oral solution Oral
19037	Xanthomax 300 tablets (Ashbourne Pharmaceuticals Ltd) Allopurinol 300mg Tablet Oral
64906	Allopurinol 300mg tablets (Almus Pharmaceuticals Ltd) Allopurinol 300mg Tablet Oral
42859	Adenuric 80mg tablets (A. Menarini Farmaceutica Internazionale SRL) Febuxostat 80mg Tablet Oral
41520	Allopurinol 300mg tablets (IVAX Pharmaceuticals UK Ltd) Allopurinol 300mg Tablet Oral
34566	Allopurinol 300mg tablets (Mylan) Allopurinol 300mg Tablet Oral
43336	Adenuric 120mg tablets (A. Menarini Farmaceutica Internazionale SRL) Febuxostat 120mg Tablet Oral
67256	Allopurinol 300mg tablets (Alliance Healthcare (Distribution) Ltd) Allopurinol 300mg Tablet Oral
72223	Allopurinol 300mg tablets (Crescent Pharma Ltd) Allopurinol 300mg Tablet Oral
34711	Allopurinol 100mg tablets (Mylan) Allopurinol 100mg Tablet Oral
44240	Cosuric 300mg Tablet (DDSA Pharmaceuticals Ltd) Allopurinol 300mg Tablet Oral
24215	Caplenal 100mg tablets (Teva UK Ltd) Allopurinol 100mg Tablet Oral
67748	Allopurinol 100mg tablets (Almus Pharmaceuticals Ltd) Allopurinol 100mg Tablet Oral
34930	Allopurinol 100mg tablets (Wockhardt UK Ltd) Allopurinol 100mg Tablet Oral
76616	Allopurinol 300mg Tablet (Celltech Pharma Europe Ltd) Allopurinol 300mg Tablet Oral
30768	Allopurinol 100mg tablets (A A H Pharmaceuticals Ltd) Allopurinol 100mg Tablet Oral
57739	Allopurinol 100mg/5ml oral solution Allopurinol 20mg/1ml Oral solution Oral
19201	Allopurinol 100mg tablets (IVAX Pharmaceuticals UK Ltd) Allopurinol 100mg Tablet Oral
33484	Allopurinol 100mg tablets (Actavis UK Ltd) Allopurinol 100mg Tablet Oral
34947	Allopurinol 100mg Tablet (Lagap) Allopurinol 100mg Tablet Oral
65820	Allopurinol 200mg/5ml oral suspension Allopurinol 40mg/1ml Oral suspension Oral
71008	Allopurinol 100mg tablets (Milpharm Ltd) Allopurinol 100mg Tablet Oral
41541	Allopurinol 100mg tablets (Teva UK Ltd) Allopurinol 100mg Tablet Oral
76	Allopurinol 300mg tablets Allopurinol 300mg Tablet Oral

74645	Allopurinol 300mg Tablet (Regent Laboratories Ltd) Allopurinol 300mg Tablet Oral
34278	Allopurinol 300mg tablets (A A H Pharmaceuticals Ltd) Allopurinol 300mg Tablet Oral
78470	Allopurinol 300mg tablets (Bristol Laboratories Ltd) Allopurinol 300mg Tablet Oral
78713	Allopurinol 50mg/5ml oral suspension Allopurinol 10mg/1ml Oral suspension Oral
77467	Zyloric 100mg tablets (Lexon (UK) Ltd) Allopurinol 100mg Tablet Oral
34573	Allopurinol 300mg tablets (Wockhardt UK Ltd) Allopurinol 300mg Tablet Oral
11975	Allopurinol 100mg/5ml sugar free oral suspension Allopurinol 100mg/5ml Suspension Sugar-Free Oral
68025	Allopurinol 100mg tablets (DE Pharmaceuticals) Allopurinol 100mg Tablet Oral
76324	Allopurinol 100mg tablets (Accord Healthcare Ltd) Allopurinol 100mg Tablet Oral
7805	Zyloric 300mg tablets (Aspen Pharma Trading Ltd) Allopurinol 300mg Tablet Oral
42536	Febuxostat 80mg tablets Febuxostat 80mg Tablet Oral
413	Allopurinol 100mg tablets Allopurinol 100mg Tablet Oral
41664	Allopurinol 100mg Tablet (Celltech Pharma Europe Ltd) Allopurinol 100mg Tablet Oral
71717	Uricto 300mg tablets (Ennogen Pharma Ltd) Allopurinol 300mg Tablet Oral
78521	Allopurinol 300mg tablets (Accord Healthcare Ltd) Allopurinol 300mg Tablet Oral
5182	Xanthomax 100 tablets (Ashbourne Pharmaceuticals Ltd) Allopurinol 100mg Tablet Oral
17255	Hamarin 300 Tablet (Roche Products Ltd) Allopurinol 300mg Tablet Oral
72153	Allopurinol 100mg tablets (Waymade Healthcare Plc) Allopurinol 100mg Tablet Oral
45352	Allopurinol 300mg tablets (Ranbaxy (UK) Ltd) Allopurinol 300mg Tablet Oral
13467	Caplenal 300mg tablets (Teva UK Ltd) Allopurinol 300mg Tablet Oral
52409	Allopurinol 100mg/5ml oral suspension Allopurinol 20mg/1ml Oral suspension Oral
43161	Febuxostat 120mg tablets Febuxostat 120mg Tablet Oral

Oral diuretics

DRUG_CODE_
17462
33837
60020
66517
64907
1288
60780
38995
12651
25965
5728
40247
27447
26217
47616
3293
21182
57488
64066
18903
78257
78262
7543
19258
43915

67664

38964

1211
13246
47647

10627

2982
9143

21231
59030
4068
31013
3997

24190

9456

11133

57610
31820
21849
77681
5721
20538
34374

30773

12318

22923

38882
8836

2811
76323

1369
27489

62249
7799
39447

8058
6
29583

60620

11864

59884

3056

4983
26529

47573
43523
30272
55

43508

11338

45916

72086
58
41828

25382
27664
43516

26219

11526

33724

3050

34449

67780

13352

31773

9223

24268
19683

71682

8558

9680

74800
22525

7066

77386

3691
66149

7351

56975
34006

46675

39021
25625
23486
24693
23382

21423

19056

46952

35162

6786

29529

74172

4406
12546
64677

57908

12054
24702

793

76840
74942
30592

52887

23456

60258

1125

16632
4044

55399

47844
21848
2612

9374

33353

2772
30129

14126

24189
20785
49268

55259

41556

50607

77449

28738
22004

23134
31932
61365

56244

48099

4034

42488

51258
542
18716

11469

74017
64798
4332
31548

72914

31150
12313

38634

66997

77466

30519

3203
19352

2255

8673
60465
33415

52900

27689
66017

923
3285

19892
71950

13871

47804
41897

42142

24632

24280

2979
56375

37294
30625

40190
21867
54679

4796

9431

47727

25505

29634

69445

2002
54341

35380

6877
37400

4211
3902
46302

20426

38831
20779
20513

23131

1301

39807

46687

77641

55358

27946

37710
19192
7618

8987
19015
77850

62207

16060
25764

14057
24832
14387
605
23271
53812

24484

53508

31470
814

4852

2046

39137

34622

581
58224

25730
61846

6437

48039
13525
51983
76157
44168
25334

17960

40149

66598

32094
34557

32277

9178
25462

12926
49752
77387

37747

72042

3793
15493
12294
1209

14837
562
3054
59834

38865

52858

6794
1170

69277

18497

14587

72083
8602

52189

33527

9764

18726

30875

3526

1721

764
26328

22539
23256

67801

5218
41405

18332

46116
25051

12226

69108

71115
67910
45305

18287
2833

6309
34551
34750

18200
69334

15341
7698

10422

26741

1124

26248

10267

62911

40886

31375
19687

52045

56
74153

12517

42388

62771

8102

77406

16786

8897

37978
30035

37650

2493
71406

20066
29696

8189
4334

18743

26220

8521

78229
2681

5868
15457

1213
30368
19194
46525

39227

8061

78374

34012
32896
31670
18096
75957

71377

17655

19055
74926

38367
7806
15577
7888

4873

15031

74040

1776
26120
32918

19890

13435

34034
76165

34803

34825
75260

14438

55160

62537

71618

65636

16161
21025

52145

33659
32002
62066
48132
24741
7709
27696

17149

18983
9211

78220

62376

35481

4429
40898

5727

65602
3517

48745
16701

31131
8052

7961

23505

21938
19695
34934

5220

46792
77333

48098

56760

21346

193
27556
13472

39147

60007

12547
29976

15042

14870

78488
64255
55050
36767
60603

41885

22839
73152

28177

7734

6359

24893

67767

17252
41861
34613
34059
16498
18244

65504

57600

26256

26275
73441
55777
63237

47467
41889

79677
3287

60149
17720

73480

34124

348
25717
79798

33658
18650

8891
71871

52276
25075

70509

1297
28157
18267

53220

75069

211
27555

35196
21803

18263
74677
23483
36190
30367

41572

34602

10882
30365
46930

77457
18973

15874
39602
54643

41719

17689

73337
16206
76934
11351

1021

76920

38855

19142

56157

46916
56051

39242

15127
53674

7136
27926

78441

74215
12360
46948

1520

55548
2179

5330

13526
26807

48870
41517
68068
2

78728

37725

71748

2001

67737

924

30034
8303

70922
25630

38459
46699

5189
26675

11384

74874

29427

56204

14228

8147
74381

36519
64745

11561

4661
7625
54825
66195
12354

11265

15108

38632
6816
79904
25787

18202
63227

37908
2495

1788

8464

20093

4540
20057
11487

22912

10902

2961
2788

34280

59616

12440
29780

63890
30913

46715

69338

27256
58078
77162

17561
4705
12110

40738

32166
73171

11448

28129

517

62337
11268
3962

60354

6160
54329

68432

33651
6118

38889
9783

9935

73993

24835

57539

49529

40907
62024
65583
77566

18606
59939
56804

70989
24520
19611
19721

3118

7441

25363

12367
73195

59911

59412

7740
47815

15135
26292
79368
32091
44254
3248

56296

14738

12456

3701
27690
7606

59290
31235
10066

8369

31708

77414

4258

25500

62700
63555

34367

21873

18361

14283
17143

6468

19300
55889
61475
29991

70754

5416

55738
34324
14761

38818
5249

10323

15488

78265

74008

27520

34899

13363

4605
22658
3548

78887
10392

7582

8623

23427

67738

75855

5112

70650

20431
60291

46355

10781
53967
4182
75383

76031
42906
23492
43184

9240
7641
62516
41292
1060
8526
74814
48079

11641
80095

71348
69009

15811
26731

18733

24008

54316
22242

1251
33083

10316

41533

41630

54201

57796

38901

27957

43322

81319

81326
81555

81590
81633

81641

81668

81772

82036

82312

82319

82328

82330

82378

82413

82488

82490

82505

82567
82639
82828

DESCRIPTION
Bisoprolol 10mg / Hydrochlorothiazide 6.25mg tablets Bisoprolol fumarate/Hydrochlorothiazide 10mg + 6.25mg Tablet Oral
Amiloride 5mg tablets (A A H Pharmaceuticals Ltd) Amiloride hydrochloride 5mg Tablet Oral
Indapamide 1.5mg modified-release tablets (Waymade Healthcare Plc) Indapamide 1.5mg Modified-release tablet Oral
Bendroflumethiazide 1.25mg/5ml oral suspension Bendroflumethiazide 250microgram/1ml Oral suspension Oral
Bendroflumethiazide 5mg tablets (Almus Pharmaceuticals Ltd) Bendroflumethiazide 5mg Tablet Oral
Tenoret 50mg/12.5mg tablets (AstraZeneca UK Ltd) Atenolol/Chlortalidone 50mg + 12.5mg Tablet Oral
Generic Sevikar HCT 20mg/5mg/12.5mg tablets Hydrochlorothiazide/Amlodipine besilate/Olmesartan medoxomil 12.5mg + 5mg + 20mg Tablet Oral
Zestoretic 20 tablets (AstraZeneca UK Ltd) Lisinopril/Hydrochlorothiazide 20mg + 12.5mg Tablet Oral
Timolol 10mg / Bendroflumethiazide 2.5mg tablets Timolol maleate/Bendroflumethiazide 10mg + 2.5mg Tablet Oral
Co-amilofruse 2.5mg/20mg tablets (Wockhardt UK Ltd) Amiloride hydrochloride/Furosemide 2.5mg + 20mg Tablet Oral
Furosemide 40mg/5ml oral solution sugar free Furosemide 8mg/1ml Oral solution Gastroenteral/Oral
Furosemide 10mg/ml Injection (Martindale Pharmaceuticals Ltd) Furosemide 10mg/ml Injection Intravenous Injection
Furosemide 40mg tablets (Wockhardt UK Ltd) Furosemide 40mg Tablet Oral
Berkamil 5mg Tablet (Berk Pharmaceuticals Ltd) Amiloride hydrochloride 5mg Tablet Oral
Sevikar HCT 40mg/10mg/12.5mg tablets (Daiichi Sankyo UK Ltd) Hydrochlorothiazide/Olmesartan medoxomil/Amlodipine besilate 12.5mg + 40mg + 10mg Tablet Oral
Moduretic Oral solution (Bristol-Myers Squibb Pharmaceuticals Ltd) Amiloride Hydrochloride/Hydrochlorothiazide Oral Solution Oral

Hydrochlorothiazide with timolol and amiloride 25mg with 10mg with 2.5mg Tablet Amiloride Hydrochloride/Hydrochlorothiazide/Timolol Maleate 25mg + 10mg + 2.5mg Tablet Oral
Hydrochlorothiazide Oral solution Hydrochlorothiazide Oral Solution

Indapamide 2.5mg/5ml oral suspension Indapamide 500microgram/1ml Oral suspension Oral
Olmesartan medoxomil 20mg / Hydrochlorothiazide 25mg tablets Hydrochlorothiazide/Olmesartan medoxomil 25mg + 20mg Tablet Oral
Furosemide 80mg/5ml oral suspension Furosemide 16mg/1ml Oral suspension Oral
Lisinopril 10mg / Hydrochlorothiazide 12.5mg tablets (Waymade Healthcare Plc) Lisinopril/Hydrochlorothiazide 10mg + 12.5mg Tablet Oral
Kalten capsules (M & A Pharmachem Ltd) Atenolol/Amiloride hydrochloride/Hydrochlorothiazide 50mg + 2.5mg + 25mg Capsule Oral
Furosemide 50mg/5ml solution for injection ampoules Furosemide 10mg/1ml Solution for injection Intravenous/Intramuscular
Olmetec Plus 40mg/12.5mg tablets (Daiichi Sankyo UK Ltd) Olmesartan medoxomil/Hydrochlorothiazide 40mg + 12.5mg Tablet Oral

Valsartan 160mg / Hydrochlorothiazide 12.5mg tablets (Teva UK Ltd)
Valsartan/Hydrochlorothiazide 160mg + 12.5mg Tablet Oral
Adizem-SR 120mg tablets (Napp Pharmaceuticals Ltd) Diltiazem hydrochloride 120mg Modified-release tablet Oral

Bendroflumethiazide 2.5mg / Potassium chloride 630mg (potassium 8.4mmol) modified-release tablets Potassium chloride/Bendroflumethiazide 630mg + 2.5mg Modified-release tablet Oral
Chlorothiazide 150mg/5ml oral suspension Chlorothiazide 30mg/1ml Oral suspension Oral
Co-amilofruse oral liquid Amiloride Hydrochloride/Furosemide Oral Liquid Oral
Co-Betaloc tablets (Pfizer Ltd) Metoprolol tartrate/Hydrochlorothiazide 100mg + 12.5mg Tablet Oral
Zestoretic 20- 20mg+12.5mg Tablet (AstraZeneca UK Ltd) Lisinopril/Hydrochlorothiazide 20mg + 12.5mg Tablet Oral
Viskaldix tablets (Advanz Pharma) Pindolol/Clopamide 10mg + 5mg Tablet Oral
Caralpha 20mg/12.5mg tablets (Actavis UK Ltd) Lisinopril/Hydrochlorothiazide 20mg + 12.5mg Tablet Oral
Furosemide 20mg/5ml oral solution Furosemide 4mg/1ml Oral solution Oral
Dytac 50mg capsules (Advanz Pharma) Triamterene 50mg Capsule Oral
Mersalyl 50mg/ml Injection Mersalyl 50mg/ml Injection
Hygroton 50mg tablets (Alliance Pharmaceuticals Ltd) Chlortalidone 50mg Tablet Oral
Neo-bendromax 5mg Tablet (Ashbourne Pharmaceuticals Ltd) Bendroflumethiazide 5mg Tablet Oral
Amiloride 5mg / furosemide 40mg tablets Amiloride Hydrochloride/Furosemide 5mg + 40mg Tablets Oral
Hydrochlorothiazide with captopril 25mg with 50mg Tablet Captopril/Hydrochlorothiazide 25mg + 50mg Tablet Oral
Furosemide 20mg/5ml oral solution sugar free (Advanz Pharma) Furosemide 4mg/1ml Oral solution Oral
Bendroflumethiazide 5mg tablets (Wockhardt UK Ltd) Bendroflumethiazide 5mg Tablet Oral
Dryptal 40mg Tablet (Berk Pharmaceuticals Ltd) Furosemide 40mg Tablet Oral
Bendroflumethiazide 5mg tablets (Mylan) Bendroflumethiazide 5mg Tablet Oral
Co-tenidone 100mg/25mg tablets Atenolol/Chlortalidone 100mg + 25mg Tablet Oral
Frumax 40mg Tablet (Ashbourne Pharmaceuticals Ltd) Furosemide 40mg Tablet Oral
Furosemide 40mg tablets (Teva UK Ltd) Furosemide 40mg Tablet Oral
Co-amilofruse 5mg+40mg Tablet (Berk Pharmaceuticals Ltd) Amiloride hydrochloride/Furosemide 5mg + 40mg Tablet Oral
Lasix 250mg/25ml Injection (Hoechst Marion Roussel) Furosemide 10mg/1ml Solution for injection Intravenous/Intramuscular
Hydrochlorothiazide with amiloride 50mg with 5mg Tablet Amiloride Hydrochloride/Hydrochlorothiazide 50mg + 5mg Tablet Oral
Adizem-XL 240mg capsules (Napp Pharmaceuticals Ltd) Diltiazem hydrochloride 240mg Modified-release capsule Oral
Chlorothiazide 500mg tablets Chlorothiazide 500mg Tablets Oral
Adizem sr 180mg Modified-release capsule (Napp Pharmaceuticals Ltd) Diltiazem Hydrochloride 180mg Modified-Release Capsule Oral
Furosemide 40mg tablets (Crescent Pharma Ltd) Furosemide 40mg Tablet Oral
Furosemide with amiloride 40mg+5mg Tablet Amiloride Hydrochloride/Furosemide 40mg+5mg Tablet Oral
CHLOROTHIAZIDE 25 MG LIQ

Co-amilozide 5mg/50mg tablets (Alliance Healthcare (Distribution) Ltd) Amiloride hydrochloride/Hydrochlorothiazide 5mg + 50mg Tablet Oral
Lasix 20mg tablets (Borg Medicare) Furosemide 20mg Tablet Oral
Varbim XL 1.5mg tablets (Teva UK Ltd) Indapamide 1.5mg Modified-release tablet Oral
Normetic Tablet (Abbott Laboratories Ltd) Amiloride hydrochloride/Hydrochlorothiazide 5mg + 50mg Tablet Oral
Furosemide 40mg tablets Furosemide 40mg Tablet Oral
MIN-I-JET 100MG FRUSEMIDE INJ INJ
Adizem-XL 240mg capsules (Waymade Healthcare Plc) Diltiazem hydrochloride 240mg Modified-release capsule Oral
Valsartan 160mg / Hydrochlorothiazide 12.5mg tablets Valsartan/Hydrochlorothiazide 160mg + 12.5mg Tablet Oral

Furosemide 20mg tablets (Phoenix Healthcare Distribution Ltd) Furosemide 20mg Tablet Oral
Natrilix SR 1.5mg tablets (Servier Laboratories Ltd) Indapamide 1.5mg Modified-release tablet Oral
Atenolol with amiloride and hydrochlorothiazide capsules Amiloride Hydrochloride/Atenolol/Hydrochlorothiazide Capsules Oral
Furosemide with penbutolol Tablet Furosemide/Penbutolol Tablet Oral

Sevikar HCT 40mg/5mg/12.5mg tablets (Daiichi Sankyo UK Ltd) Hydrochlorothiazide/Amlodipine besilate/Olmesartan medoxomil 12.5mg + 5mg + 40mg Tablet Oral
Amiloride 5mg tablets (Mylan) Amiloride hydrochloride 5mg Tablet Oral
Benthiazide with Triamterene capsules Triamterene/Benzthiazide Capsules Oral
Furosemide 20mg tablets Furosemide 20mg Tablet Oral
Co-amilofruse 5mg/40mg tablets (Sandoz Ltd) Amiloride hydrochloride/Furosemide 5mg + 40mg Tablet Oral
Bendroflumethiazide 5mg with Nadolol 40mg tablets Bendroflumethiazide/Nadolol 5mg + 40mg Tablets Oral
Hydroflumethiazide with spironolactone 50mg+50mg Tablet Spironolactone/Hydroflumethiazide 50mg+50mg Tablet Oral
Valsartan 160mg / Hydrochlorothiazide 12.5mg tablets (Actavis UK Ltd) Valsartan/Hydrochlorothiazide 160mg + 12.5mg Tablet Oral
Bendroflumethiazide 5mg tablets Bendroflumethiazide 5mg Tablet Oral
Furosemide 500mg tablets (Accord Healthcare Ltd) Furosemide 500mg Tablet Oral
Co-Diovan 160mg/25mg tablets (Novartis Pharmaceuticals UK Ltd) Valsartan/Hydrochlorothiazide 160mg + 25mg Tablet Oral
FRUSEMIDE PAED
Indapamide 2.5mg tablets (Actavis UK Ltd) Indapamide hemihydrate 2.5mg Tablet Oral
Zida-co 5mg+50mg Tablet (Opus Pharmaceuticals Ltd) Amiloride hydrochloride/Hydrochlorothiazide 5mg + 50mg Tablet Oral
CoAprovel 300mg/12.5mg tablets (Sanofi) Irbesartan/Hydrochlorothiazide 300mg + 12.5mg Tablet Oral
Diuril 250mg/5ml oral suspension (Imported (United States)) Chlorothiazide 50mg/1ml Oral suspension Oral
Furosemide with triamterene 40mgwith50mg Tablet Furosemide/Triamterene 40mg + 50mg Tablet Oral

Co-tenidone 50mg/12.5mg tablets (Mylan) Atenolol/Chlortalidone 50mg + 12.5mg Tablet Oral

Bendroflumethiazide 5mg tablets (DE Pharmaceuticals) Bendroflumethiazide 5mg Tablet Oral
Midamor 5mg Tablet (MSD Thomas Morson Pharmaceuticals) Amiloride hydrochloride 5mg Tablet Oral
Co-amilofruse 5mg/40mg tablets (Wockhardt UK Ltd) Amiloride hydrochloride/Furosemide 5mg + 40mg Tablet Oral
Triamterene with hydrochlorothiazide 50mg + 25mg Tablet Hydrochlorothiazide/Triamterene 50mg + 25mg Tablet Oral
Hydrochlorothiazide with valsartan 12.5mg with 80mg Tablet Hydrochlorothiazide/Valsartan 12.5mg + 80mg Tablet Oral
BURINEX K
Losartan 100mg / Hydrochlorothiazide 25mg tablets (Mawdsley-Brooks & Company Ltd) Losartan potassium/Hydrochlorothiazide 100mg + 25mg Tablet Oral
Adizem xl 120mg Capsule (Napp Pharmaceuticals Ltd) Diltiazem Hydrochloride 120mg Capsule Oral
Frusol 40mg/5ml oral solution (Rosemont Pharmaceuticals Ltd) Furosemide 8mg/1ml Oral solution Gastroenteral/Oral
Frumil 40mg/5mg tablets (Lexon (UK) Ltd) Amiloride hydrochloride/Furosemide 5mg + 40mg Tablet Oral
CHLOROTHIAZIDE 250 MG SYR
Metoprolol 100mg / Hydrochlorothiazide 12.5mg tablets Metoprolol tartrate/Hydrochlorothiazide 100mg + 12.5mg Tablet Oral
Dyazide 50mg/25mg tablets (Dowelhurst Ltd) Triamterene/Hydrochlorothiazide 50mg + 25mg Tablet Oral
Sotalol 160mg with hydrochlorothiazide 25mg tablet Hydrochlorothiazide/Sotalol Hydrochloride 160mg + 25mg Tablets Oral
Furosemide 4.5mg/5ml oral solution Furosemide 900microgram/1ml Oral solution Oral
Bendroflumethiazide 2.5mg/5ml oral suspension Bendroflumethiazide 500microgram/1ml Oral suspension Oral
Losartan 50mg / Hydrochlorothiazide 12.5mg tablets (A A H Pharmaceuticals Ltd) Losartan potassium/Hydrochlorothiazide 50mg + 12.5mg Tablet Oral
Furosemide 40mg tablets (Actavis UK Ltd) Furosemide 40mg Tablet Oral
Indapamide 1.5mg modified-release tablets (A A H Pharmaceuticals Ltd) Indapamide 1.5mg Modified-release tablet Oral
Hydrochlorothiazide with olmesartan medoxomil 25mg with 20mg tablet Hydrochlorothiazide/Olmesartan Medoxomil 25mg + 20mg Tablet Oral
FRUSEMIDE 40mg/8MMOL POTASSIUM S/R
FRUSEMIDE 40mg/10MMOL POTASSIUM S/R
CARACE (SPECIAL COMPLIANCE PACK)
CARACE (SPECIAL COMPLIANCE PACK)
Cozaar-Comp 100mg/25mg tablets (Merck Sharp & Dohme Ltd) Losartan potassium/Hydrochlorothiazide 100mg + 25mg Tablet Oral
Furosemide 50mg/5ml sugar free Oral solution (Rosemont Pharmaceuticals Ltd) Furosemide 10mg/1ml Oral solution Oral
Co-tenidone 100mg/25mg tablets (Actavis UK Ltd) Atenolol/Chlortalidone 100mg + 25mg Tablet Oral
Furosemide 20mg/2ml solution for injection ampoules Furosemide 10mg/1ml Solution for injection Intravenous/Intramuscular
Lisinopril 10mg / Hydrochlorothiazide 12.5mg tablets Lisinopril/Hydrochlorothiazide 10mg + 12.5mg Tablet Oral

Hydroflumethiazide with spironolactone 25mg+25mg Tablet Spironolactone/Hydroflumethiazide 25mg+25mg Tablet Oral
Indapamide 1.5mg modified-release tablets (Sigma Pharmaceuticals Plc) Indapamide 1.5mg Modified-release tablet Oral

Decaserpyl plus Tablet (Roussel Laboratories Ltd) Benzthiazide/Methoserpidine Tablet Oral
Kalspare Tablet (Dominion Pharma) Chlortalidone/Triamterene 50mg + 50mg Tablet Oral
Furosemide 40mg tablets (DE Pharmaceuticals) Furosemide 40mg Tablet Oral
Co-amilofruse 5mg/40mg tablets (Kent Pharmaceuticals Ltd) Amiloride hydrochloride/Furosemide 5mg + 40mg Tablet Oral
Propranolol 80mg / Bendroflumethiazide 2.5mg capsules Propranolol hydrochloride/Bendroflumethiazide 80mg + 2.5mg Capsule Oral
FRUSEMIDE
Adizem xl 240mg Capsule (Napp Pharmaceuticals Ltd) Diltiazem hydrochloride 240mg Modified-release capsule Oral
Actelsar HCT 40mg/12.5mg tablets (Actavis UK Ltd) Telmisartan/Hydrochlorothiazide 40mg + 12.5mg Tablet Oral
Chlortalidone 50mg/5ml oral suspension Chlortalidone 10mg/1ml Oral suspension Oral
CHLOROTHIAZIDE / SPIRONOLACTONE / GLUCOS 60 MG POW
Furosemide 20mg/2ml solution for injection ampoules (A A H Pharmaceuticals Ltd) Furosemide 10mg/1ml Solution for injection Intravenous/Intramuscular
Hydrochlorothiazide with valsartan 25mg with 160mg Tablet Hydrochlorothiazide/Valsartan 25mg + 160mg Tablet Oral
Co-amilofruse 2.5mg/20mg tablets (Milpharm Ltd) Amiloride hydrochloride/Furosemide 2.5mg + 20mg Tablet Oral
Navidrex -k Tablet (Novartis Pharmaceuticals UK Ltd) Potassium Chloride/Cyclopenthiazide Tablet Oral

Hydrosaluric 50mg tablets (Merck Sharp & Dohme Ltd) Hydrochlorothiazide 50mg Tablet Oral
Diurexan 20mg tablets (Mylan) Xipamide 20mg Tablet Oral
Lisinopril 20mg / Hydrochlorothiazide 12.5mg tablets (A A H Pharmaceuticals Ltd) Lisinopril/Hydrochlorothiazide 20mg + 12.5mg Tablet Oral
Bendroflumethiazide 2.5mg tablets (Kent Pharmaceuticals Ltd) Bendroflumethiazide 2.5mg Tablet Oral
AMILOSPARE 5 MG TAB
Indapamide 2.5mg tablets Indapamide hemihydrate 2.5mg Tablet Oral
Adizem 60mg Modified-release tablet (Napp Pharmaceuticals Ltd) Diltiazem hydrochloride 60mg Modified-release tablet Oral
Lisinopril 20mg / Hydrochlorothiazide 12.5mg tablets (Teva UK Ltd) Lisinopril/Hydrochlorothiazide 20mg + 12.5mg Tablet Oral
Lasoride 5mg/40mg tablets (Sanofi) Amiloride hydrochloride/Furosemide 5mg + 40mg Tablet Oral
Abicol Tablet (Knoll Ltd) Bendroflumethiazide/Reserpine Tablet Oral
Acebutolol 200mg / Hydrochlorothiazide 12.5mg tablets Acebutolol hydrochloride/Hydrochlorothiazide 200mg + 12.5mg Tablet Oral
Neo-bendromax 2.5mg Tablet (Ashbourne Pharmaceuticals Ltd) Bendroflumethiazide 2.5mg Tablet Oral
FRUMIL
Furosemide 50mg/5ml oral suspension Furosemide 10mg/1ml Oral suspension Oral

Indapamide 2.5mg tablets (Kent Pharmaceuticals Ltd) Indapamide hemihydrate 2.5mg Tablet Oral
Co-amilozide 5mg/50mg tablets (Teva UK Ltd) Amiloride hydrochloride/Hydrochlorothiazide 5mg + 50mg Tablet Oral
Perindopril arginine 2mg with Indapamide 625 micrograms tablet Indapamide/Perindopril Erbumine
Natrilix SR 1.5mg tablets (Waymade Healthcare Plc) Indapamide 1.5mg Modified-release tablet Oral
Methyldopa with hydrochlorothiazide Tablet Hydrochlorothiazide/Methyldopa Anhydrous Tablet Oral
CARACE (SPECIAL COMPLIANCE PACK)
Nadolol 40mg / Bendroflumethiazide 5mg tablets Nadolol/Bendroflumethiazide 40mg + 5mg Tablet Oral
Bumetanide 1mg tablets (C P Pharmaceuticals Ltd) Bumetanide 1mg Tablet Oral
Furosemide 40mg/5ml oral suspension Furosemide 8mg/1ml Oral suspension Oral
Lisinopril 20mg / Hydrochlorothiazide 12.5mg tablets (Tillomed Laboratories Ltd) Lisinopril/Hydrochlorothiazide 20mg + 12.5mg Tablet Oral
Indapamide 2.5mg tablets (A A H Pharmaceuticals Ltd) Indapamide hemihydrate 2.5mg Tablet Oral
Amiloride 5mg / hydrochlorothiazide 50mg/5ml solution Amiloride Hydrochloride/Hydrochlorothiazide 5mg + 50mg/5ml Solution Oral
Furosemide 40mg/5ml oral solution sugar free (A A H Pharmaceuticals Ltd) Furosemide 8mg/1ml Oral solution Gastroenteral/Oral
Coversyl Arginine Plus 5mg/1.25mg tablets (DE Pharmaceuticals) Perindopril arginine/Indapamide 5mg + 1.25mg Tablet Oral
Hydrochlorothiazide 25mg tablets Hydrochlorothiazide 25mg Tablet Oral
Dryptal 10mg/ml Injection (Berk Pharmaceuticals Ltd) Furosemide 10mg/ml Injection
Irbesartan 300mg / Hydrochlorothiazide 12.5mg tablets Irbesartan/Hydrochlorothiazide 300mg + 12.5mg Tablet Oral
Moduretic 5mg/50mg tablets (Waymade Healthcare Plc) Amiloride hydrochloride/Hydrochlorothiazide 5mg + 50mg Tablet Oral
Chlorothiazide 120mg/5ml oral solution Chlorothiazide 24mg/1ml Oral solution Oral
Metolazone 5mg tablets Metolazone 5mg Tablet Oral
Furosemide 20mg tablets (Actavis UK Ltd) Furosemide 20mg Tablet Oral
Bendroflumethiazide 2.5mg Tablet (Celltech Pharma Europe Ltd) Bendroflumethiazide 2.5mg Tablet Oral
Co-amilozide 5mg/50mg tablets (IVAX Pharmaceuticals UK Ltd) Amiloride hydrochloride/Hydrochlorothiazide 5mg + 50mg Tablet Oral
Carace 20mg tablets (Bristol-Myers Squibb Pharmaceuticals Ltd) Lisinopril 20mg Tablet Oral
Adizem-XL 300mg capsules (Napp Pharmaceuticals Ltd) Diltiazem hydrochloride 300mg Modified-release capsule Oral
MicardisPlus 40mg/12.5mg tablets (Waymade Healthcare Plc) Telmisartan/Hydrochlorothiazide 40mg + 12.5mg Tablet Oral
Co-tenidone 50mg/12.5mg tablets (Kent Pharmaceuticals Ltd) Atenolol/Chlortalidone 50mg + 12.5mg Tablet Oral
Amiloride with timolol with hydrochlorothiazide tablets Amiloride Hydrochloride/Hydrochlorothiazide/Timolol Maleate Tablets Oral
Capozide LS Tablet (E R Squibb and Sons Ltd) Hydrochlorothiazide/Captopril 12.5mg + 25mg Tablet Oral
Xuret 0.5mg Tablet (Galen Ltd) Metolazone 0.5mg Tablet Oral

Navispare 2.5mg/250microgram tablets (Advanz Pharma) Amiloride hydrochloride/Cyclopenthiazide 2.5mg + 250microgram Tablet Oral
Oxprenolol with cyclopenthiazide 160mg+0.25mg Modified-release tablet Oxprenolol Hydrochloride/Cyclopenthiazide 160mg+0.25mg Modified-Release Tablet Oral
Furosemide 5mg/5ml oral solution Furosemide 1mg/1ml Oral solution Oral
Bendroflumethiazide 2.5mg tablets (Mylan) Bendroflumethiazide 2.5mg Tablet Oral
Furosemide 80mg/8ml solution for injection Minijet pre-filled syringes (UCB Pharma Ltd)
Furosemide 10mg/1ml Solution for injection Intravenous/Intramuscular
Bendroflumethiazide 2.5mg tablets (IVAX Pharmaceuticals UK Ltd) Bendroflumethiazide 2.5mg Tablet Oral
Furosemide 20mg tablets (Almus Pharmaceuticals Ltd) Furosemide 20mg Tablet Oral
Co-amilozide 5mg/50mg tablets Amiloride hydrochloride/Hydrochlorothiazide 5mg + 50mg Tablet Oral
AMILORIDE S/F 5 MG/5ML SOL
Serpasil -esidrex Tablet (Novartis Pharmaceuticals UK Ltd) Hydrochlorothiazide/Reserpine Tablet Oral
Furosemide 20mg tablets (Waymade Healthcare Plc) Furosemide 20mg Tablet Oral
Co-prenozide 160mg/0.25mg modified-release tablets Oxprenolol hydrochloride/Cyclopenthiazide 160mg + 250microgram Modified-release tablet Oral
Co-triamterzide 50mg/25mg tablets (A A H Pharmaceuticals Ltd) Triamterene/Hydrochlorothiazide 50mg + 25mg Tablet Oral
CO-BETALOC
Moduretic 5mg/50mg tablets (Merck Sharp & Dohme Ltd) Amiloride hydrochloride/Hydrochlorothiazide 5mg + 50mg Tablet Oral
Hydrochlorothiazide with losartan 25mg with 100mg Tablet Hydrochlorothiazide/Losartan Potassium 25mg + 100mg Tablet Oral
Totaretic 100mg+25mg Tablet (C P Pharmaceuticals Ltd) Atenolol/Chlortalidone 100mg + 25mg Tablet Oral
Centyl k Tablet (Edwin Burgess Ltd) Potassium chloride/Bendroflumethiazide 630mg + 2.5mg Modified-release tablet Oral
Furosemide 40mg tablets (Accord Healthcare Ltd) Furosemide 40mg Tablet Oral
Triamterene with chlortalidone 50mg + 25mg Tablet Chlortalidone/Triamterene 50mg + 25mg Tablet Oral
Furosemide 20mg tablets (A A H Pharmaceuticals Ltd) Furosemide 20mg Tablet Oral

Torasemide iv 20mg/4ml Intravenous injection Torasemide 20mg/4ml Intravenous Injection
Berkozide 5mg Tablet (Berk Pharmaceuticals Ltd) Bendroflumethiazide 5mg Tablet Oral
Chlorothiazide 250mg tablets Chlorothiazide 250mg Tablet Oral
Inderetic 80mg/2.5mg capsules (AstraZeneca UK Ltd) Propranolol hydrochloride/Bendroflumethiazide 80mg + 2.5mg Capsule Oral
Frusemek 40mg+5mg Tablet (Approved Prescription Services Ltd) Amiloride hydrochloride/Furosemide 5mg + 40mg Tablet Oral

Sevikar HCT 40mg/5mg/25mg tablets (Daiichi Sankyo UK Ltd) Hydrochlorothiazide/Amlodipine besilate/Olmesartan medoxomil 25mg + 5mg + 40mg Tablet Oral
Spiro-co 50mg+50mg Tablet (IVAX Pharmaceuticals UK Ltd) Spironolactone/Hydroflumethiazide 50mg + 50mg Tablet Oral
Olmetec Plus 20mg/12.5mg tablets (Daiichi Sankyo UK Ltd) Hydrochlorothiazide/Olmesartan medoxomil 12.5mg + 20mg Tablet Oral

Furosemide 40mg/5ml oral solution sugar free (Sigma Pharmaceuticals Plc) Furosemide 8mg/1ml Oral solution Gastroenteral/Oral
Amiloride 5mg / hydrochlorothiazide 50mg tablets Amiloride Hydrochloride/Hydrochlorothiazide 5mg + 50mg Tablets Oral
Chlorothiazide 5mg/5ml oral suspension Chlorothiazide 1mg/1ml Oral suspension Oral
Hydrochlorothiazide with olmesartan medoxomil 12.5mg with 20mg tablet Hydrochlorothiazide/Olmesartan Medoxomil 12.5mg + 20mg Tablet Oral
Co-Diovan 160mg/12.5mg tablets (Novartis Pharmaceuticals UK Ltd) Valsartan/Hydrochlorothiazide 160mg + 12.5mg Tablet Oral
MIN-I-JET 80MG FRUSEMIDE INJ MG INJ
Furosemide with amiloride 20mg+2.5mg Tablet Amiloride Hydrochloride/Furosemide 20mg+2.5mg Tablet Oral
FRUSEMIDE 20MG/SPIRONOLACTONE 50MG MG CAP
Neo-Naclex 2.5mg tablets (Advanz Pharma) Bendroflumethiazide 2.5mg Tablet Oral
Centyl k 2.5mg+7.7mmol Tablet (Edwin Burgess Ltd) Bendroflumethiazide/Potassium chloride 2.5mg + 573mg Modified-release tablet Oral
Adizem-SR 180mg capsules (Napp Pharmaceuticals Ltd) Diltiazem hydrochloride 180mg Modified-release capsule Oral
MODURETIC
LASIX 10 MG INJ

Bendroflumethiazide 5mg with Propanolol 160mg modified-release capsules Bendroflumethiazide/Propranolol Hydrochloride 5mg + 160mg Modified Release Capsules Oral
Frumil ls 20mg+2.5mg Tablet (Helios Healthcare Ltd) Amiloride hydrochloride/Furosemide 2.5mg + 20mg Tablet Oral

Frumil 40mg/5mg tablets (Sanofi) Amiloride hydrochloride/Furosemide 5mg + 40mg Tablet Oral

Olmesartan medoxomil with amlodipine and hydrochlorothiazide 20mg + 5mg + 12.5mg Tablet Amlodipine/Hydrochlorothiazide/Olmesartan Medoxomil 20mg + 5mg + 12.5mg Tablet Oral
CoAprovel 300mg/12.5mg tablets (Mawdsley-Brooks & Company Ltd) Irbesartan/Hydrochlorothiazide 300mg + 12.5mg Tablet Oral

Olmesartan medoxomil with amlodipine and hydrochlorothiazide 40mg + 10mg + 25mg Tablet Amlodipine/Hydrochlorothiazide/Olmesartan Medoxomil 40mg + 10mg + 25mg Tablet
Nadolol 80mg / Bendroflumethiazide 5mg tablets Nadolol/Bendroflumethiazide 80mg + 5mg Tablet Oral
Lisinopril 10mg / Hydrochlorothiazide 12.5mg tablets (Teva UK Ltd) Lisinopril/Hydrochlorothiazide 10mg + 12.5mg Tablet Oral
Furosemide 40mg Tablet (M & A Pharmachem Ltd) Furosemide 40mg Tablet Oral
Xipamide 20mg tablets Xipamide 20mg Tablet Oral
Propranolol 160mg modified-release / Bendroflumethiazide 5mg capsules Propranolol hydrochloride/Bendroflumethiazide 160mg + 5mg Modified-release capsule Oral
ADIZEM CONTINUS 120 MG TAB
Chlorothiazide 500mg/5ml oral solution Chlorothiazide 100mg/1ml Oral solution Oral
Adizem-SR 120mg capsules (Waymade Healthcare Plc) Diltiazem hydrochloride 120mg Modified-release capsule Oral
Valsartan 80mg / Hydrochlorothiazide 12.5mg tablets Valsartan/Hydrochlorothiazide 80mg + 12.5mg Tablet Oral
PINDOLOL 10MG/CLOPAMIDE 5MG

Pindolol 10mg / Clopamide 5mg tablets Pindolol/Clopamide 10mg + 5mg Tablet Oral
Lasipressin Tablet (Hoechst UK Ltd) Furosemide/Penbutolol Tablet Oral
Carace 5mg tablets (Bristol-Myers Squibb Pharmaceuticals Ltd) Lisinopril 5mg Tablet Oral
Chlortalidone 50mg tablets Chlortalidone 50mg Tablet Oral
FRUSEMIDE (2ML)
Bendroflumethiazide oral solution Bendroflumethiazide
Hydrochlorothiazide with valsartan 12.5mg with 160mg Tablet Hydrochlorothiazide/Valsartan 12.5mg + 160mg Tablet Oral
Spironolactone 5mg/5ml / Chlorothiazide 50mg/5ml oral suspension
Spironolactone/Chlorothiazide 1mg/1ml + 10mg/1ml Oral suspension Oral

Tenchlor 50mg/12.5mg tablets (Teva UK Ltd) Atenolol/Chlortalidone 50mg + 12.5mg Tablet Oral
Bumetanide 1mg tablets Bumetanide 1mg Tablet Oral
Adizem sr 120mg Modified-release tablet (Napp Pharmaceuticals Ltd) Diltiazem hydrochloride 120mg Modified-release tablet Oral

Navidrex 500microgram tablets (Advanz Pharma) Cyclopenthiazide 500microgram Tablet Oral
Zestoretic 10 tablets (AstraZeneca UK Ltd) Lisinopril/Hydrochlorothiazide 10mg + 12.5mg Tablet Oral
Co-amilofruse 10mg/80mg tablets (Wockhardt UK Ltd) Amiloride hydrochloride/Furosemide 10mg + 80mg Tablet Oral
Atenolol 50mg with Chlortalidone 12.5mg tablets Atenolol/Chlortalidone 50mg+12.5mg Tablets Oral
Furosemide 10mg/5ml oral solution Furosemide 2mg/1ml Oral solution Oral
Timolol maleate with amiloride and hydrochlorothiazide Tablet Amiloride Hydrochloride/Hydrochlorothiazide/Timolol Maleate Tablet Oral
Zaroxolyn 2.5mg tablets (IDIS) Metolazone 2.5mg Tablet Oral
Losartan 50mg / Hydrochlorothiazide 12.5mg tablets Losartan potassium/Hydrochlorothiazide 50mg + 12.5mg Tablet Oral
Losartan 100mg / Hydrochlorothiazide 12.5mg tablets (Teva UK Ltd) Hydrochlorothiazide/Losartan potassium 12.5mg + 100mg Tablet Oral
Hydrenox 50mg Tablet (Knoll Ltd) Hydroflumethiazide 50mg Tablet Oral
Furosemide 5mg/5ml oral suspension Furosemide 1mg/1ml Oral suspension Oral
Furosemide 50mg/5ml oral solution Furosemide 10mg/1ml Oral solution Oral
Indipam XL 1.5mg tablets (Actavis UK Ltd) Indapamide 1.5mg Modified-release tablet Oral
Furosemide 500mg tablets (A A H Pharmaceuticals Ltd) Furosemide 500mg Tablet Oral
Furosemide 20mg / Potassium chloride 750mg (potassium 10mmol) modified-release tablets Potassium chloride/Furosemide 750mg + 20mg Modified-release tablet Oral
Bendroflumethiazide 5mg tablets (IVAX Pharmaceuticals UK Ltd) Bendroflumethiazide 5mg Tablet Oral
Losartan 50mg / Hydrochlorothiazide 12.5mg tablets (Lupin Healthcare (UK) Ltd) Losartan potassium/Hydrochlorothiazide 50mg + 12.5mg Tablet Oral
Co-tenidone 50mg/12.5mg tablets (A A H Pharmaceuticals Ltd) Atenolol/Chlortalidone 50mg + 12.5mg Tablet Oral
Furosemide 40mg tablets (IVAX Pharmaceuticals UK Ltd) Furosemide 40mg Tablet Oral
Furosemide 80mg/8ml solution for injection pre-filled syringes Furosemide 10mg/1ml Solution for injection Intravenous/Intramuscular
Atenolol 25mg / Bendroflumethiazide 1.25mg capsules Atenolol/Bendroflumethiazide 25mg + 1.25mg Capsule Oral
Clopamide 5mg with Pindolol 10mg tablets Clopamide/Pindolol 5mg + 10mg Tablets Oral

Polythiazide 1mg tablets Polythiazide 1mg Tablet Oral
Metolazone 2.5mg/5ml oral solution Metolazone 500microgram/1ml Oral solution Oral
Viskaldix tablets (Waymade Healthcare Plc) Pindolol/Clopamide 10mg + 5mg Tablet Oral
Cozaar-Comp 100mg/12.5mg tablets (Merck Sharp & Dohme Ltd) Hydrochlorothiazide/Losartan potassium 12.5mg + 100mg Tablet Oral
Bendroflumethiazide 2.5mg tablets (Alliance Healthcare (Distribution) Ltd) Bendroflumethiazide 2.5mg Tablet Oral
Co-amilofruse 10mg/80mg tablets Amiloride hydrochloride/Furosemide 10mg + 80mg Tablet Oral
Reserpine with hydrochlorothiazide tablet Hydrochlorothiazide/Reserpine Tablets Oral
Burinex 5mg tablets (LEO Pharma) Bumetanide 5mg Tablet Oral
Neo-Naclex 5mg tablets (Mercury Pharma Group Ltd) Bendroflumethiazide 5mg Tablet Oral
Frusol 50mg/5ml oral solution (Rosemont Pharmaceuticals Ltd) Furosemide 10mg/1ml Oral solution Oral
Furosemide 10mg/ml Injection Furosemide 10mg/ml Injection
Hygroton 100mg Tablet (Alliance Pharmaceuticals Ltd) Chlortalidone 100mg Tablet Oral
Chlorothiazide 250mg/5ml oral solution Chlorothiazide 50mg/1ml Oral solution Oral
Adizem-XL 120mg capsules (Napp Pharmaceuticals Ltd) Diltiazem hydrochloride 120mg Modified-release capsule Oral
Co-Diovan 80mg/12.5mg tablets (Sigma Pharmaceuticals Plc) Valsartan/Hydrochlorothiazide 80mg + 12.5mg Tablet Oral
Perindopril erbumine 4mg / Indapamide 1.25mg tablets Perindopril erbumine/Indapamide 4mg + 1.25mg Tablet Oral
Cyclopenthiazide 500microgram tablets Cyclopenthiazide 500microgram Tablet Oral
Adizem-XL 240mg capsules (Lexon (UK) Ltd) Diltiazem hydrochloride 240mg Modified-release capsule Oral
Amiloride 10mg / furosemide 80mg tablets Amiloride Hydrochloride/Furosemide 10mg + 80mg Tablets Oral
Amiloride 5mg / Bumetanide 1mg tablets Bumetanide/Amiloride hydrochloride 1mg + 5mg Tablet Oral
Bendroflumethiazide 5mg tablets (Waymade Healthcare Plc) Bendroflumethiazide 5mg Tablet Oral
Metenix 5mg tablets (Sanofi) Metolazone 5mg Tablet Oral
Losartan 100mg / Hydrochlorothiazide 25mg tablets (A A H Pharmaceuticals Ltd) Losartan potassium/Hydrochlorothiazide 100mg + 25mg Tablet Oral
Co-amilofruse 5mg/40mg tablets (Mylan) Amiloride hydrochloride/Furosemide 5mg + 40mg Tablet Oral
Carace 20 Tablet (Bristol-Myers Squibb Pharmaceuticals Ltd) Lisinopril/Hydrochlorothiazide 20mg + 12.5mg Tablet Oral
Triamaxco 50mg/25mg tablets (Ashbourne Pharmaceuticals Ltd) Triamterene/Hydrochlorothiazide 50mg + 25mg Tablet Oral
Furosemide 250mg/25ml solution for injection ampoules Furosemide 10mg/1ml Solution for injection Intravenous/Intramuscular
Amiloride with atenolol with hydrochlorothiazide capsules Amiloride Hydrochloride/Atenolol/Hydrochlorothiazide Capsules Oral
Dyazide 50mg/25mg tablets (Advanz Pharma) Triamterene/Hydrochlorothiazide 50mg + 25mg Tablet Oral
Co-Diovan 80mg/12.5mg tablets (Novartis Pharmaceuticals UK Ltd) Valsartan/Hydrochlorothiazide 80mg + 12.5mg Tablet Oral
LASIX (25ML)

LASIX (2ML)
LASIX PAED
Dyazide 50mg/25mg tablets (Lexon (UK) Ltd) Triamterene/Hydrochlorothiazide 50mg + 25mg Tablet Oral

Bumetanide 1mg/5ml oral solution sugar free Bumetanide 200microgram/1ml Oral solution Oral
Furosemide 500mg tablets (Teva UK Ltd) Furosemide 500mg Tablet Oral
Aridil 20mg+2.5mg Tablet (C P Pharmaceuticals Ltd) Amiloride hydrochloride/Furosemide 2.5mg + 20mg Tablet Oral
Furosemide 10mg/ml Injection (Antigen Pharmaceuticals) Furosemide 10mg/ml Injection Intravenous Injection
CAPOZIDE

Burinex 1mg/5ml Oral solution (LEO Pharma) Bumetanide 200microgram/1ml Oral solution Oral
Adizem-XL 300mg capsules (Lexon (UK) Ltd) Diltiazem hydrochloride 300mg Modified-release capsule Oral
Zestoretic 10 tablets (Waymade Healthcare Plc) Lisinopril/Hydrochlorothiazide 10mg + 12.5mg Tablet Oral
Furosemide 40mg/5ml oral solution Furosemide 8mg/1ml Oral solution Oral
Bumetanide 1mg tablets (Teva UK Ltd) Bumetanide 1mg Tablet Oral
Co-Betaloc SA tablets (Pfizer Ltd) Metoprolol tartrate/Hydrochlorothiazide 200mg + 25mg Modified-release tablet Oral
CYCLOPENTHIAZIDE -K tablets Potassium Chloride/Cyclopenthiazide Tablets Oral
Adizem xl 300mg Capsule (Napp Pharmaceuticals Ltd) Diltiazem hydrochloride 300mg Modified-release capsule Oral
Indapamide 2.5mg tablets (Mylan) Indapamide hemihydrate 2.5mg Tablet Oral
Amiloride 5mg tablets (Actavis UK Ltd) Amiloride hydrochloride 5mg Tablet Oral
Olmesartan medoxomil 20mg / Hydrochlorothiazide 12.5mg tablets Hydrochlorothiazide/Olmesartan medoxomil 12.5mg + 20mg Tablet Oral
Bendroflumethiazide 5mg with Nadolol 80mg tablets Bendroflumethiazide/Nadolol
Burinex 0.5mg/ml Injection (LEO Pharma) Bumetanide 500microgram/1ml Solution for injection Intramuscular/Intravenous
Aprinox 5mg tablets (Amdipharm Plc) Bendroflumethiazide 5mg Tablet Oral
Lasix 50mg/5ml Injection (Hoechst UK Ltd) Furosemide 10mg/1ml Solution for injection Intravenous/Intramuscular
Totaretic 50mg+12.5mg Tablet (C P Pharmaceuticals Ltd) Atenolol/Chlortalidone 50mg + 12.5mg Tablet Oral
Tenoretic 100mg/25mg tablets (AstraZeneca UK Ltd) Atenolol/Chlortalidone 100mg + 25mg Tablet Oral

Tenchlor 100mg/25mg tablets (Teva UK Ltd) Atenolol/Chlortalidone 100mg + 25mg Tablet Oral
Adizem-XL 200mg capsules (Napp Pharmaceuticals Ltd) Diltiazem hydrochloride 200mg Modified-release capsule Oral
Losartan 50mg / Hydrochlorothiazide 12.5mg tablets (Teva UK Ltd) Losartan potassium/Hydrochlorothiazide 50mg + 12.5mg Tablet Oral
Bendroflumethiazide 2.5mg tablets (Almus Pharmaceuticals Ltd) Bendroflumethiazide 2.5mg Tablet Oral
Amilamont 5mg/5ml oral solution sugar free (Rosemont Pharmaceuticals Ltd) Amiloride hydrochloride 1mg/1ml Oral solution Oral
MODUCREN

Furosemide 250mg/5ml solution for injection vials Furosemide 10mg/1ml Solution for injection Intravenous/Intramuscular

Co-amilofruse 5mg/40mg tablets Amiloride hydrochloride/Furosemide 5mg + 40mg Tablet Oral
Chlorothiazide 10mg/5ml oral solution Chlorothiazide 2mg/1ml Oral solution Oral
Timolol maleate with bendroflumethiazide 20mg + 5mg Tablet Bendroflumethiazide/Timolol Maleate 20mg + 5mg Tablet Oral
Furosemide 40mg/5ml oral solution sugar free (Advanz Pharma) Furosemide 8mg/1ml Oral solution Gastroenteral/Oral
Indapamide 1.5mg modified-release tablets (DE Pharmaceuticals) Indapamide 1.5mg Modified-release tablet Oral
Furosemide 40mg / Potassium chloride 600mg (potassium 8mmol) modified-release tablets Potassium chloride/Furosemide 600mg + 40mg Modified-release tablet Oral
Natrilix 2.5mg tablets (Mawdsley-Brooks & Company Ltd) Indapamide hemihydrate 2.5mg Tablet Oral
Chlortalidone 25mg with Atenolol 100mg tablets Atenolol/Chlortalidone 25mg+100mg Tablets Oral
Triam-Co 50mg/25mg tablets (IVAX Pharmaceuticals UK Ltd) Triamterene/Hydrochlorothiazide 50mg + 25mg Tablet Oral
Perindopril arginine 5mg / Indapamide 1.25mg tablets Perindopril arginine/Indapamide 5mg + 1.25mg Tablet Oral
CHLOROTHIAZIDE 40MG /SPIRONOLACTONE 4MG POW
Losartan 100mg / Hydrochlorothiazide 12.5mg tablets Hydrochlorothiazide/Losartan potassium 12.5mg + 100mg Tablet Oral
Burinex A 5mg/1mg tablets (LEO Pharma) Bumetanide/Amiloride hydrochloride 1mg + 5mg Tablet Oral
Furosemide 1mg/5ml oral solution Furosemide 200microgram/1ml Oral solution Oral
Amil-Co 5mg/50mg tablets (IVAX Pharmaceuticals UK Ltd) Amiloride hydrochloride/Hydrochlorothiazide 5mg + 50mg Tablet Oral
Methoserpidine with benzthiazide Tablet Benzthiazide/Methoserpidine Tablet Oral
Secadrex 200mg/12.5mg tablets (Sanofi) Acebutolol hydrochloride/Hydrochlorothiazide 200mg + 12.5mg Tablet Oral
Metolazone 500microgram low dose Tablet Metolazone 500microgram Tablet Oral
Tenben 25mg/1.25mg capsules (Galen Ltd) Atenolol/Bendroflumethiazide 25mg + 1.25mg Capsule Oral
Delvas Tablet (Berk Pharmaceuticals Ltd) Amiloride hydrochloride/Hydrochlorothiazide 5mg + 50mg Tablet Oral
Spironolactone 25mg with hydroflumethiazide 25mg tablet Spironolactone/Hydroflumethiazide 25mg+25mg Tablets Oral
Furosemide 20mg/2ml solution for injection ampoules (Wockhardt UK Ltd) Furosemide 10mg/1ml Solution for injection Intravenous/Intramuscular
AMILORIDE 10 MG TAB
Frusol 20mg/5ml oral solution (Rosemont Pharmaceuticals Ltd) Furosemide 4mg/1ml Oral solution Oral
Baycaron 25mg Tablet (Bayer Plc) Mefruside 25mg Tablet Oral
Neo-Naclex-K modified-release tablets (Mercury Pharma Group Ltd) Potassium chloride/Bendroflumethiazide 630mg + 2.5mg Modified-release tablet Oral
CHLOROTHIAZIDE/SPIRONOLACTONE SACHETS 100 MG
Furosemide 20mg tablets (Teva UK Ltd) Furosemide 20mg Tablet Oral
Torasemide 5mg tablets (Teva UK Ltd) Torasemide 5mg Tablet Oral

Capozide LS 12.5mg/25mg tablets (Bristol-Myers Squibb Pharmaceuticals Ltd) Hydrochlorothiazide/Captopril 12.5mg + 25mg Tablet Oral
Sotalol 80mg with hydrochlorothiazide 12.5mg tablet Hydrochlorothiazide/Sotalol Hydrochloride 80mg + 12.5mg Tablets Oral
Bendroflumethiazide 1.25mg with Potassium 573mg modified-release tablets Potassium Chloride/Bendroflumethiazide
Co-tenidone 100mg/25mg tablets (IVAX Pharmaceuticals UK Ltd) Atenolol/Chlortalidone 100mg + 25mg Tablet Oral
Furosemide 40mg tablets (Ranbaxy (UK) Ltd) Furosemide 40mg Tablet Oral
Bendroflumethiazide 2.5mg tablets (Teva UK Ltd) Bendroflumethiazide 2.5mg Tablet Oral
Torasemide 10mg tablets Torasemide 10mg Tablet Oral
Chlorothiazide 5mg/5ml oral solution Chlorothiazide 1mg/1ml Oral solution Oral
Frumil LS 20mg/2.5mg tablets (Waymade Healthcare Plc) Amiloride hydrochloride/Furosemide 2.5mg + 20mg Tablet Oral
Carace 10 Tablet (Bristol-Myers Squibb Pharmaceuticals Ltd) Lisinopril/Hydrochlorothiazide 10mg + 12.5mg Tablet Oral
Chlortalidone 12.5mg with Atenolol 50mg tablets Atenolol/Chlortalidone 12.5mg+50mg Tablets Oral
Furosemide 20mg tablets (Crescent Pharma Ltd) Furosemide 20mg Tablet Oral
Hydrochlorothiazide with losartan 12.5mg with 100mg Tablet Hydrochlorothiazide/Losartan Potassium 12.5mg + 100mg Tablet Oral
Bumetanide 5mg tablets Bumetanide 5mg Tablet Oral
HYDROCHLOROTHIAZIDE 12.5MG/K 8.1MMOL S/R 12.5 MG TAB
FRUSEMIDE 40MG/8MMOL POTASSIUM S/R MG TAB
Fru-Co 5mg/40mg tablets (Teva UK Ltd) Amiloride hydrochloride/Furosemide 5mg + 40mg Tablet Oral
Accuretic 10mg/12.5mg tablets (Pfizer Ltd) Hydrochlorothiazide/Quinapril hydrochloride 12.5mg + 10mg Tablet Oral

Zestoretic 20 tablets (Lexon (UK) Ltd) Lisinopril/Hydrochlorothiazide 20mg + 12.5mg Tablet Oral
Burinex K modified-release tablets (LEO Pharma) Bumetanide/Potassium chloride 500microgram + 573mg Modified-release tablet Oral
CHLOROTHIAZIDE 50 MG SUS
Furosemide 20mg tablets (Sandoz Ltd) Furosemide 20mg Tablet Oral
Hydrochlorothiazide with amiloride 25mgwith2.5mg Tablet Amiloride Hydrochloride/Hydrochlorothiazide 25mg+2.5mg Tablet Oral
Frumil Forte 10mg/80mg tablets (Sanofi) Amiloride hydrochloride/Furosemide 10mg + 80mg Tablet Oral
Co-tenidone 50mg/12.5mg tablets (IVAX Pharmaceuticals UK Ltd) Atenolol/Chlortalidone 50mg + 12.5mg Tablet Oral
Amiloride 5mg tablets (Accord Healthcare Ltd) Amiloride hydrochloride 5mg Tablet Oral
Bendroflumethiazide 2.5mg Tablet (Regent Laboratories Ltd) Bendroflumethiazide 2.5mg Tablet Oral
Co-tenidone 50mg/12.5mg tablets (Teva UK Ltd) Atenolol/Chlortalidone 50mg + 12.5mg Tablet Oral
Zaroxolyn 2.5mg tablets (Imported (Canada)) Metolazone 2.5mg Tablet Oral
Corgaretic 80mg tablets (Sanofi-Synthelabo Ltd) Nadolol/Bendroflumethiazide 80mg + 5mg Tablet Oral
Cozaar-Comp 50mg/12.5mg tablets (Sigma Pharmaceuticals Plc) Losartan potassium/Hydrochlorothiazide 50mg + 12.5mg Tablet Oral

Co-tenidone 100mg/25mg tablets (DE Pharmaceuticals) Atenolol/Chlortalidone 100mg + 25mg Tablet Oral
Losartan 100mg / Hydrochlorothiazide 12.5mg tablets (A A H Pharmaceuticals Ltd) Hydrochlorothiazide/Losartan potassium 12.5mg + 100mg Tablet Oral
Adizem-XL 120mg capsules (Lexon (UK) Ltd) Diltiazem hydrochloride 120mg Modified-release capsule Oral
Telmisartan 80mg / Hydrochlorothiazide 12.5mg tablets Telmisartan/Hydrochlorothiazide 80mg + 12.5mg Tablet Oral
Prestim forte Tablet (LEO Pharma) Bendroflumethiazide/Timolol Maleate Tablet Oral
Cyclopenthiazide 0.25mg with oxprenolol 160mg modified-release tablets Oxprenolol Hydrochloride/Cyclopenthiazide
Hydrochlorothiazide with metoprolol tartrate 25mg with 200mg Modified-release tablet Hydrochlorothiazide/Metoprolol Tartrate 25mg + 200mg Modified-Release Tablet Oral
Etacrynic 50mg/vial injection Etacrynic Acid 50mg/vial Injection
Cardide SR 1.5mg tablets (Teva UK Ltd) Indapamide 1.5mg Modified-release tablet Oral
Hydrochlorothiazide Capsule Hydrochlorothiazide Capsule Oral
FRUSEMIDE
Arelix 6mg Capsule (Hoechst Marion Roussel) Piretanide 6mg Capsule Oral
Furosemide 40mg tablets (Kent Pharmaceuticals Ltd) Furosemide 40mg Tablet Oral
Monozide 10 tablets (Wyeth Pharmaceuticals) Bisoprolol fumarate/Hydrochlorothiazide 10mg + 6.25mg Tablet Oral
Brinaldix k Effervescent tablet (Berk Pharmaceuticals Ltd) Potassium Chloride/Clopamide Effervescent Tablet Oral
ADIZEM-XL 180 MG CAP

Bendroflumethiazide 5mg tablets (Sovereign Medical Ltd) Bendroflumethiazide 5mg Tablet Oral
Actelsar HCT 80mg/12.5mg tablets (Actavis UK Ltd) Telmisartan/Hydrochlorothiazide 80mg + 12.5mg Tablet Oral
Irbesartan 300mg / Hydrochlorothiazide 25mg tablets Hydrochlorothiazide/Irbesartan 25mg + 300mg Tablet Oral
Trasidrex modified-release tablets (Mercury Pharma Group Ltd) Oxprenolol hydrochloride/Cyclopenthiazide 160mg + 250microgram Modified-release tablet Oral
Torasemide 5mg tablets (A A H Pharmaceuticals Ltd) Torasemide 5mg Tablet Oral
Amiloride 2.5mg / Cyclopenthiazide 250microgram tablets Amiloride hydrochloride/Cyclopenthiazide 2.5mg + 250microgram Tablet Oral
Adizem-XL 120mg capsules (Waymade Healthcare Plc) Diltiazem hydrochloride 120mg Modified-release capsule Oral
Hydrochlorothiazide 50mg tablets Hydrochlorothiazide 50mg Tablet Oral
Timolol 10mg / Amiloride 2.5mg / Hydrochlorothiazide 25mg tablets Amiloride hydrochloride/Timolol maleate/Hydrochlorothiazide 2.5mg + 10mg + 25mg Tablet Oral
Carace 10mg tablets (Bristol-Myers Squibb Pharmaceuticals Ltd) Lisinopril 10mg Tablet Oral
Spiro-co 25mg+25mg Tablet (IVAX Pharmaceuticals UK Ltd) Spironolactone/Hydroflumethiazide 25mg + 25mg Tablet Oral
Torasemide 5mg tablets Torasemide 5mg Tablet Oral
Spironolactone 50mg with hydroflumethiazide 50mg tablet Spironolactone/Hydroflumethiazide 50mg+50mg Tablets Oral

Adizem xl plus 150mg+12.5mg Modified-release capsule (Napp Pharmaceuticals Ltd) Diltiazem Hydrochloride/Hydrochlorothiazide 150mg+12.5mg Modified-Release Capsule Oral

Froop Co 5mg/40mg tablets (Ashbourne Pharmaceuticals Ltd) Amiloride hydrochloride/Furosemide 5mg + 40mg Tablet Oral
AMILORIDE HYDROCHLORIDE
Bumetanide 1mg tablets (Mylan) Bumetanide 1mg Tablet Oral
Furosemide with amiloride 80mg+10mg Tablet Amiloride Hydrochloride/Furosemide 80mg+10mg Tablet Oral

Olmesartan medoxomil with amlodipine and hydrochlorothiazide 40mg + 5mg + 12.5mg Tablet Amlodipine/Hydrochlorothiazide/Olmesartan Medoxomil 40mg + 5mg + 12.5mg Tablet Oral
Aluzine 20mg Tablet (M A Steinhard Ltd) Furosemide 20mg Tablet Oral
Perindopril arginine 4mg with Indapamide 1.25mg tablet Indapamide/Perindopril Erbumine 4mg + 1.25mg Tablets Oral

Indapamide 2.5mg tablets (Strides Pharma UK Ltd) Indapamide hemihydrate 2.5mg Tablet Oral
Hydromet Tablet (MSD Thomas Morson Pharmaceuticals) Hydrochlorothiazide/Methyldopa Anhydrous Tablet Oral
Co-amilofruse 2.5mg/20mg tablets Amiloride hydrochloride/Furosemide 2.5mg + 20mg Tablet Oral
DYAZIDE
ESIDREX-K TAB
Carace 20 Plus tablets (Merck Sharp & Dohme Ltd) Lisinopril/Hydrochlorothiazide 20mg + 12.5mg Tablet Oral
Generic Sevikar HCT 40mg/10mg/12.5mg tablets Hydrochlorothiazide/Olmesartan medoxomil/Amlodipine besilate 12.5mg + 40mg + 10mg Tablet Oral
Triamterene 50mg / Chlortalidone 50mg tablets Chlortalidone/Triamterene 50mg + 50mg Tablet Oral
SOTAZIDE
Tolerzide Tablet (Bristol-Myers Squibb Pharmaceuticals Ltd) Hydrochlorothiazide/Sotalol Hydrochloride Tablet Oral
Telmisartan 40mg / Hydrochlorothiazide 12.5mg tablets Telmisartan/Hydrochlorothiazide 40mg + 12.5mg Tablet Oral
Bendroflumethiazide 5mg/5ml oral suspension Bendroflumethiazide 1mg/1ml Oral suspension Oral
Furosemide 2mg/5ml oral solution Furosemide 400microgram/1ml Oral solution Oral
CHLOROTHIAZIDE/SPIRONOLACTONE/LACT SACH 50 MG
Bumetanide 1mg tablets (IVAX Pharmaceuticals UK Ltd) Bumetanide 1mg Tablet Oral
Chlorothiazide 50mg/5ml oral suspension Chlorothiazide 10mg/1ml Oral suspension Oral
Ethibide XL 1.5mg tablets (Genus Pharmaceuticals Ltd) Indapamide 1.5mg Modified-release tablet Oral
Clopamide with Potassium effervescent tablets Potassium Chloride/Clopamide Effervescent Tablet Oral
Bumetanide 1mg tablets (Waymade Healthcare Plc) Bumetanide 1mg Tablet Oral
Hydrochlorothiazide with atenolol and amiloride Capsule Amiloride Hydrochloride/Atenolol/Hydrochlorothiazide Capsule Oral
Diumide-K Continus tablets (Teofarma) Potassium chloride/Furosemide 600mg + 40mg Modified-release tablet Oral
Zestoretic 10- 10mg+12.5mg Tablet (AstraZeneca UK Ltd) Lisinopril/Hydrochlorothiazide 10mg + 12.5mg Tablet Oral

Amilospare Tablet (Ashbourne Pharmaceuticals Ltd) Amiloride hydrochloride 5mg Tablet Oral

Lisinopril 10mg / Hydrochlorothiazide 12.5mg tablets (Almus Pharmaceuticals Ltd) Lisinopril/Hydrochlorothiazide 10mg + 12.5mg Tablet Oral

Esidrex 25mg Tablet (Novartis Pharmaceuticals UK Ltd) Hydrochlorothiazide 25mg Tablet Oral
Tensaid XL 1.5mg tablets (Mylan) Indapamide 1.5mg Modified-release tablet Oral
Bumetanide 5mg tablets (Teva UK Ltd) Bumetanide 5mg Tablet Oral
Bendroflumethiazide 2.5mg tablets (Actavis UK Ltd) Bendroflumethiazide 2.5mg Tablet Oral
Kalspare tablets (DHP Healthcare Ltd) Chlortalidone/Triamterene 50mg + 50mg Tablet Oral
ENDURONYL TAB
Adizem-SR 180mg capsules (Lexon (UK) Ltd) Diltiazem hydrochloride 180mg Modified-release capsule Oral
Furosemide 20mg/2ml solution for injection ampoules (Alliance Healthcare (Distribution) Ltd) Furosemide 10mg/1ml Solution for injection Intravenous/Intramuscular

Opumide 2.5mg Tablet (Opus Pharmaceuticals Ltd) Indapamide hemihydrate 2.5mg Tablet Oral

Nindaxa 2.5 tablets (Ashbourne Pharmaceuticals Ltd) Indapamide hemihydrate 2.5mg Tablet Oral
Chlorothiazide 200mg/5ml oral solution Chlorothiazide 40mg/1ml Oral solution Oral
Metolazone 5mg/5ml oral solution Metolazone 1mg/1ml Oral solution Oral
Furosemide 20mg tablets (Boston Healthcare Ltd) Furosemide 20mg Tablet Oral

Olmesartan medoxomil with amlodipine and hydrochlorothiazide 40mg + 5mg + 25mg Tablet Amlodipine/Hydrochlorothiazide/Olmesartan Medoxomil 40mg + 5mg + 25mg Tablet Oral
TRIAMTERENE 50MG HYDROCHLOROTHIAZIDE25MG

Furosemide 20mg/2ml Injection (Celltech Pharma Europe Ltd) Furosemide 20mg/2ml Injection
Furosemide 1mg/ml Oral solution Furosemide 1mg/ml Oral Solution Oral

Amiloride 5mg/5ml oral suspension Amiloride hydrochloride 1mg/1ml Oral suspension Oral
Saluric 500mg Tablet (Merck Sharp & Dohme Ltd) Chlorothiazide 500mg Tablet Oral
Furosemide 20mg/5ml sugar free Oral solution (Rosemont Pharmaceuticals Ltd) Furosemide 4mg/1ml Oral solution Oral

Bendroflumethiazide 5mg tablets (Accord Healthcare Ltd) Bendroflumethiazide 5mg Tablet Oral
Moduretic Tablet (Bristol-Myers Squibb Pharmaceuticals Ltd) Amiloride hydrochloride/Hydrochlorothiazide 5mg + 50mg Tablet Oral
Furosemide 40mg tablets (Mylan) Furosemide 40mg Tablet Oral
Chlorothiazide 500mg tablets Chlorothiazide 500mg Tablet Oral
Co-amilofruse 5mg/40mg tablets (A A H Pharmaceuticals Ltd) Amiloride hydrochloride/Furosemide 5mg + 40mg Tablet Oral
Edecrin 50mg Tablet (Merck Sharp & Dohme Ltd) Etacrynic Acid 50mg Tablet Oral
Hygroton -k Tablet (Novartis Pharmaceuticals UK Ltd) Potassium Chloride/Chlortalidone Tablet Oral
Chlorothiazide 60mg/5ml oral suspension Chlorothiazide 12mg/1ml Oral suspension Oral
Adizem-XL 180mg capsules (DE Pharmaceuticals) Diltiazem hydrochloride 180mg Modified-release capsule Oral
FRUSEMIDE 40MG/AMILORIDE HYD 5MG

Indapamide 2.5mg tablets (DE Pharmaceuticals) Indapamide hemihydrate 2.5mg Tablet Oral

Aldactide 50 tablets (Pfizer Ltd) Spironolactone/Hydroflumethiazide 50mg + 50mg Tablet Oral
Kalspare ls Tablet (Dominion Pharma) Chlortalidone/Triamterene Tablet Oral
Enduron 5mg Tablet (Abbott Laboratories Ltd) Methyclothiazide 5mg Tablet Oral

Sevikar HCT 40mg/10mg/25mg tablets (Daiichi Sankyo UK Ltd) Hydrochlorothiazide/Olmesartan medoxomil/Amlodipine besilate 25mg + 40mg + 10mg Tablet Oral
Co-amilozide 2.5mg/25mg tablets (Mawdsley-Brooks & Company Ltd) Amiloride hydrochloride/Hydrochlorothiazide 2.5mg + 25mg Tablet Oral
Frumil 40mg+5mg Tablet (Helios Healthcare Ltd) Amiloride hydrochloride/Furosemide 5mg + 40mg Tablet Oral
BURINEX
CoAprovel 300mg/25mg tablets (Sanofi) Hydrochlorothiazide/Irbesartan 25mg + 300mg Tablet Oral
Berkozide 2.5mg Tablet (Berk Pharmaceuticals Ltd) Bendroflumethiazide 2.5mg Tablet Oral
Acezide 25mg/50mg tablets (Bristol-Myers Squibb Pharmaceuticals Ltd) Hydrochlorothiazide/Captopril 25mg + 50mg Tablet Oral
Furosemide 40mg tablets (Mawdsley-Brooks & Company Ltd) Furosemide 40mg Tablet Oral
HYDROCHLOROTHIAZIDE 12.5MG/K 8.1MMOL S/R
Furosemide 5mg/5ml oral solution sugar free Furosemide 1mg/1ml Oral solution Oral
CHLOROTHIAZIDE 60MG / SPIRONOLACTONE 6MG POW
Co-tenidone 100mg/25mg tablets (Teva UK Ltd) Atenolol/Chlortalidone 100mg + 25mg Tablet Oral
Bendroflumethiazide 2.5mg tablets (Sovereign Medical Ltd) Bendroflumethiazide 2.5mg Tablet Oral

Carace 2.5mg tablets (Bristol-Myers Squibb Pharmaceuticals Ltd) Lisinopril 2.5mg Tablet Oral
CHLOROTHIAZIDE/SPIRONOLACTONE/LACTOSE MG POW
Amiloride 5mg tablets (Wockhardt UK Ltd) Amiloride hydrochloride 5mg Tablet Oral
Capozide 25mg/50mg tablets (Dowelhurst Ltd) Hydrochlorothiazide/Captopril 25mg + 50mg Tablet Oral
Centyl 2.5mg Tablet (Edwin Burgess Ltd) Bendroflumethiazide 2.5mg Tablet Oral
Amiloride 2.5mg / furosemide 20mg tablets Amiloride Hydrochloride/Furosemide 2.5mg + 20mg Tablets Oral
Bumetanide 1mg tablets (Actavis UK Ltd) Bumetanide 1mg Tablet Oral
Metolazone Oral solution Metolazone Oral Solution
Co-amilofruse 5mg/40mg tablets (Actavis UK Ltd) Amiloride hydrochloride/Furosemide 5mg + 40mg Tablet Oral
MicardisPlus 80mg/12.5mg tablets (Boehringer Ingelheim Ltd) Telmisartan/Hydrochlorothiazide 80mg + 12.5mg Tablet Oral
Co-amilozide 5mg/50mg tablets (Wockhardt UK Ltd) Amiloride hydrochloride/Hydrochlorothiazide 5mg + 50mg Tablet Oral
Froop 40mg tablets (Ashbourne Pharmaceuticals Ltd) Furosemide 40mg Tablet Oral
Furosemide 6.5mg/5ml oral solution Furosemide 1.3mg/1ml Oral solution Oral
Co-zidocapt 25mg/50mg tablets Hydrochlorothiazide/Captopril 25mg + 50mg Tablet Oral
Innozide 20mg/12.5mg tablets (Merck Sharp & Dohme Ltd) Enalapril maleate/Hydrochlorothiazide 20mg + 12.5mg Tablet Oral
Enalapril 20mg / Hydrochlorothiazide 12.5mg tablets (Tillomed Laboratories Ltd) Enalapril maleate/Hydrochlorothiazide 20mg + 12.5mg Tablet Oral

Adizem-XL 180mg capsules (Napp Pharmaceuticals Ltd) Diltiazem hydrochloride 180mg Modified-release capsule Oral
Bendroflumethiazide 2.5mg with Timolol maleate 10mg tablets Bendroflumethiazide/Timolol Maleate 2.5mg + 10mg Tablets Oral
Perindopril tosilate 5mg / Indapamide 1.25mg tablets Perindopril tosilate/Indapamide 5mg + 1.25mg Tablet Oral
Co-amilozide 5mg/50mg tablets (A A H Pharmaceuticals Ltd) Amiloride hydrochloride/Hydrochlorothiazide 5mg + 50mg Tablet Oral
Furosemide 20mg tablets (Kent Pharmaceuticals Ltd) Furosemide 20mg Tablet Oral
Carace 10 Plus tablets (Merck Sharp & Dohme Ltd) Lisinopril/Hydrochlorothiazide 10mg + 12.5mg Tablet Oral
Hydrochlorothiazide with triamterene 25mgwith50mg Tablet Hydrochlorothiazide/Triamterene 25mg+50mg Tablet Oral
Metolazone 2.5mg tablets Metolazone 2.5mg Tablet Oral
Dytide capsules (Mercury Pharma Group Ltd) Triamterene/Benzthiazide 50mg + 25mg Capsule Oral
Furosemide 20mg tablets (Mylan) Furosemide 20mg Tablet Oral
Co-amilozide 5mg/50mg/5ml oral solution Amiloride hydrochloride/Hydrochlorothiazide 1mg/1ml + 10mg/1ml Oral solution Oral
Losartan 50mg / Hydrochlorothiazide 12.5mg tablets (DE Pharmaceuticals) Losartan potassium/Hydrochlorothiazide 50mg + 12.5mg Tablet Oral
Nephril 1mg Tablet (Pfizer Ltd) Polythiazide 1mg Tablet Oral
Furosemide 40mg tablets (Arrow Generics Ltd) Furosemide 40mg Tablet Oral
Capozide 25mg/50mg tablets (Bristol-Myers Squibb Pharmaceuticals Ltd) Hydrochlorothiazide/Captopril 25mg + 50mg Tablet Oral

Bumetanide 1mg tablets (Alliance Healthcare (Distribution) Ltd) Bumetanide 1mg Tablet Oral
Triamterene 50mg capsules Triamterene 50mg Capsule Oral
Corgaretic 40mg tablets (Sanofi-Synthelabo Ltd) Nadolol/Bendroflumethiazide 40mg + 5mg Tablet Oral
Atenix Co 100 tablets (Ashbourne Pharmaceuticals Ltd) Atenolol/Chlortalidone 100mg + 25mg Tablet Oral
TENORETIC
Adizem-SR 90mg capsules (DE Pharmaceuticals) Diltiazem hydrochloride 90mg Modified-release capsule Oral
Bendroflumethiazide 5mg tablets (Teva UK Ltd) Bendroflumethiazide 5mg Tablet Oral
Furosemide 4mg/5ml oral solution Furosemide 800microgram/1ml Oral solution Oral
Bendroflumethiazide 2.5mg tablets Bendroflumethiazide 2.5mg Tablet Oral
Co-amilofruse 5mg/40mg/5ml oral suspension Amiloride hydrochloride/Furosemide 1mg/1ml + 8mg/1ml Oral suspension Oral

Co-tenidone 100mg/25mg tablets (Mylan) Atenolol/Chlortalidone 100mg + 25mg Tablet Oral
Actelsar HCT 80mg/25mg tablets (Actavis UK Ltd) Hydrochlorothiazide/Telmisartan 25mg + 80mg Tablet Oral

Aldactide 25 tablets (Pfizer Ltd) Spironolactone/Hydroflumethiazide 25mg + 25mg Tablet Oral
Bendroflumethiazide 2.5mg tablets (Dr Reddy's Laboratories (UK) Ltd) Bendroflumethiazide 2.5mg Tablet Oral
Co-amilozide 2.5mg/25mg tablets Amiloride hydrochloride/Hydrochlorothiazide 2.5mg + 25mg Tablet Oral

CHLOROTHIAZIDE / SPIRONOLACTONE 100 MG POW
Tenavoid Tablet (Edwin Burgess Ltd) Bendroflumethiazide/Meprobamate Tablet Oral
Losartan 100mg / Hydrochlorothiazide 12.5mg tablets (Phoenix Healthcare Distribution Ltd) Hydrochlorothiazide/Losartan potassium 12.5mg + 100mg Tablet Oral
BRINALDIX K EFFERVESCENT
Telmisartan 80mg / Hydrochlorothiazide 25mg tablets Hydrochlorothiazide/Telmisartan 25mg + 80mg Tablet Oral
Furosemide 40mg tablets (Almus Pharmaceuticals Ltd) Furosemide 40mg Tablet Oral
Enalapril 20mg / Hydrochlorothiazide 12.5mg tablets Enalapril maleate/Hydrochlorothiazide 20mg + 12.5mg Tablet Oral
XIPAMIDE
Co-flumactone 50mg/50mg tablets Spironolactone/Hydroflumethiazide 50mg + 50mg Tablet Oral
Zestoretic 20 tablets (DE Pharmaceuticals) Lisinopril/Hydrochlorothiazide 20mg + 12.5mg Tablet Oral
Hydrochlorothiazide with metoprolol tartrate 12.5mg with 100mg tablet Hydrochlorothiazide/Metoprolol Tartrate 12.5mg + 100mg Tablet Oral
Losartan 50mg / Hydrochlorothiazide 12.5mg tablets (Actavis UK Ltd) Losartan potassium/Hydrochlorothiazide 50mg + 12.5mg Tablet Oral
Coversyl Plus tablets (Servier Laboratories Ltd) Perindopril erbumine/Indapamide 4mg + 1.25mg Tablet Oral
Lopresoretic Tablet (Novartis Pharmaceuticals UK Ltd) Chlortalidone/Metoprolol Tartrate Tablet Oral
Furosemide 3mg/5ml oral suspension Furosemide 600microgram/1ml Oral suspension Oral
Esidrex -k Tablet (Novartis Pharmaceuticals UK Ltd) Potassium Chloride/Hydrochlorothiazide Tablet Oral
Furosemide 8mg/5ml oral suspension Furosemide 1.6mg/1ml Oral suspension Oral

Co-zidocapt 12.5mg/25mg tablets Hydrochlorothiazide/Captopril 12.5mg + 25mg Tablet Oral
Spironolactone 50mg / Furosemide 20mg capsules Furosemide/Spironolactone 20mg + 50mg Capsule Oral
FRUSEMIDE 20MG/POTASSIUM 10MMOL S/R MG TAB
Furosemide 20mg tablets (Sigma Pharmaceuticals Plc) Furosemide 20mg Tablet Oral
Bumetanide 1mg tablets (Almus Pharmaceuticals Ltd) Bumetanide 1mg Tablet Oral
Etacrynic 50mg tablets Etacrynic Acid 50mg Tablets Oral
Triamterene 50mg / Furosemide 40mg tablets Triamterene/Furosemide 50mg + 40mg Tablet Oral
Quinapril 10mg / Hydrochlorothiazide 12.5mg tablets Hydrochlorothiazide/Quinapril hydrochloride 12.5mg + 10mg Tablet Oral
Adizem-SR 90mg capsules (Napp Pharmaceuticals Ltd) Diltiazem hydrochloride 90mg Modified-release capsule Oral
Chlorothiazide 250mg/5ml oral suspension Chlorothiazide 50mg/1ml Oral suspension Oral
Furosemide 40mg tablets (Boston Healthcare Ltd) Furosemide 40mg Tablet Oral
TOTARETIC 100 TAB
MicardisPlus 40mg/12.5mg tablets (Boehringer Ingelheim Ltd) Telmisartan/Hydrochlorothiazide 40mg + 12.5mg Tablet Oral
Chlorothiazide 70mg/5ml oral solution Chlorothiazide 14mg/1ml Oral solution Oral
Coversyl Arginine Plus 5mg/1.25mg tablets (Servier Laboratories Ltd) Perindopril arginine/Indapamide 5mg + 1.25mg Tablet Oral
Bumetanide with Amiloride tablets Amiloride Hydrochloride/Bumetanide Tablets Oral

Atenolol 100mg with Chlortalidone 25mg tablets Atenolol/Chlortalidone 100mg+25mg Tablets Oral

Meprobamate with bendroflumethiazide Tablet Bendroflumethiazide/Meprobamate Tablet Oral
Metoprolol 200mg modified-release / Hydrochlorothiazide 25mg tablets Metoprolol tartrate/Hydrochlorothiazide 200mg + 25mg Modified-release tablet Oral
Cozaar-Comp 50mg/12.5mg tablets (Merck Sharp & Dohme Ltd) Losartan potassium/Hydrochlorothiazide 50mg + 12.5mg Tablet Oral
Methyclothiazide 5mg Tablet Methyclothiazide 5mg Tablet Oral
Torasemide 2.5mg tablets Torasemide 2.5mg Tablet Oral
Bendroflumethiazide 2.5mg with Propanolol 80mg capsules Bendroflumethiazide/Propranolol Hydrochloride 2.5mg + 80mg Capsules Oral
Captopril 50mg with Hydrochlorothiazide 25mg tablets Captopril/Hydrochlorothiazide 50mg + 25mg Tablets Oral
Frusene 50mg/40mg tablets (Orion Pharma (UK) Ltd) Triamterene/Furosemide 50mg + 40mg Tablet Oral
Burinex 1mg tablets (LEO Pharma) Bumetanide 1mg Tablet Oral
Co-amilofruse 2.5mg/20mg tablets (Sandoz Ltd) Amiloride hydrochloride/Furosemide 2.5mg + 20mg Tablet Oral

Rawel XL 1.5mg tablets (Consilient Health Ltd) Indapamide 1.5mg Modified-release tablet Oral

Hydrosaluric 25mg tablets (Merck Sharp & Dohme Ltd) Hydrochlorothiazide 25mg Tablet Oral
Furosemide 20mg Tablet (C P Pharmaceuticals Ltd) Furosemide 20mg Tablet Oral
MicardisPlus 80mg/12.5mg tablets (Waymade Healthcare Plc) Telmisartan/Hydrochlorothiazide 80mg + 12.5mg Tablet Oral
Betinex 1mg Tablet (Berk Pharmaceuticals Ltd) Bumetanide 1mg Tablet Oral

Olmesartan medoxomil with amlodipine and hydrochlorothiazide 40mg + 10mg + 12.5mg Tablet Amlodipine/Hydrochlorothiazide/Olmesartan Medoxomil 40mg + 10mg + 12.5mg Tablet Oral
Furosemide 40mg/5ml sugar free Oral solution (Rosemont Pharmaceuticals Ltd) Furosemide 8mg/1ml Oral solution Gastroenteral/Oral

Bendroflumethiazide 2.5mg tablets (Wockhardt UK Ltd) Bendroflumethiazide 2.5mg Tablet Oral
Furosemide 8mg/5ml oral solution Furosemide 1.6mg/1ml Oral solution Oral
Chlorothiazide 12.5mg/5ml oral solution Chlorothiazide 2.5mg/1ml Oral solution Oral

Bendroflumethiazide 2.5mg / Potassium chloride 573mg (potassium 7.7mmol) modified-release tablets Bendroflumethiazide/Potassium chloride 2.5mg + 573mg Modified-release tablet Oral
Furosemide 20mg/2ml Injection Furosemide 20mg/2ml Injection Intravenous Injection
Hydroflumethiazide 50mg Tablet Hydroflumethiazide 50mg Tablet Oral
Torem iv 10mg/2ml Intravenous injection (Boehringer Mannheim UK Ltd) Torasemide 10mg/2ml Intravenous Injection
Capto-co 25mg+50mg Tablet (IVAX Pharmaceuticals UK Ltd) Hydrochlorothiazide/Captopril 25mg + 50mg Tablet Oral
Furosemide Oral solution Furosemide Oral Solution
Irbesartan 150mg / Hydrochlorothiazide 12.5mg tablets Hydrochlorothiazide/Irbesartan 12.5mg + 150mg Tablet Oral
Co-amilofruse 5mg/40mg tablets (Teva UK Ltd) Amiloride hydrochloride/Furosemide 5mg + 40mg Tablet Oral

Adizem sr 120mg Modified-release capsule (Napp Pharmaceuticals Ltd) Diltiazem Hydrochloride 120mg Modified-Release Capsule Oral
Irbesartan 300mg / Hydrochlorothiazide 12.5mg tablets (Actavis UK Ltd) Irbesartan/Hydrochlorothiazide 300mg + 12.5mg Tablet Oral
Torem 2.5mg tablets (Mylan) Torasemide 2.5mg Tablet Oral
TRIAMTERENE 50MG HYDROCHLOROTHIAZIDE25MG TAB
Co-amilozide 2.5mg/25mg tablets (Kent Pharmaceuticals Ltd) Amiloride hydrochloride/Hydrochlorothiazide 2.5mg + 25mg Tablet Oral

Bumetanide 500microgram / Potassium chloride 573mg (potassium 7.7mmol) modified-release tablets Bumetanide/Potassium chloride 500microgram + 573mg Modified-release tablet Oral
Metolazone 5mg/5ml oral suspension Metolazone 1mg/1ml Oral suspension Oral

Metolazone 2.5mg/5ml oral suspension Metolazone 500microgram/1ml Oral suspension Oral
Bendroflumethiazide 2.5mg tablets (A A H Pharmaceuticals Ltd) Bendroflumethiazide 2.5mg Tablet Oral
Furosemide 20mg/5ml oral solution sugar free Furosemide 4mg/1ml Oral solution Oral
MicardisPlus 80mg/25mg tablets (Boehringer Ingelheim Ltd) Hydrochlorothiazide/Telmisartan 25mg + 80mg Tablet Oral
Co-tenidone 50mg/12.5mg tablets Atenolol/Chlortalidone 50mg + 12.5mg Tablet Oral

Amiloride 5mg/5ml oral solution sugar free Amiloride hydrochloride 1mg/1ml Oral solution Oral
Frumil 40mg/5mg tablets (Sigma Pharmaceuticals Plc) Amiloride hydrochloride/Furosemide 5mg + 40mg Tablet Oral
Min-i-jet furosemide 10mg/ml Injection (Celltech Pharma Europe Ltd) Furosemide 10mg/ml Injection Intravenous Injection
Zestoretic 10 tablets (Sigma Pharmaceuticals Plc) Lisinopril/Hydrochlorothiazide 10mg + 12.5mg Tablet Oral
Indapamide 2.5mg tablets (Phoenix Healthcare Distribution Ltd) Indapamide hemihydrate 2.5mg Tablet Oral
Indapamide 2.5mg tablets (Genus Pharmaceuticals Ltd) Indapamide hemihydrate 2.5mg Tablet Oral
Bumetanide 5mg tablets (A A H Pharmaceuticals Ltd) Bumetanide 5mg Tablet Oral
Furosemide 3mg/5ml oral solution Furosemide 600microgram/1ml Oral solution Oral
Furosemide 40mg tablets (Bristol Laboratories Ltd) Furosemide 40mg Tablet Oral
Diltiazem and hydrochlorothiazide 150mg+12.5mg modified-release capsules Diltiazem Hydrochloride/Hydrochlorothiazide 150mg + 12.5mg Modified Release Capsules Oral
Furosemide 20mg/5ml oral suspension Furosemide 4mg/1ml Oral suspension Oral
Chlorothiazide 25mg/5ml oral suspension Chlorothiazide 5mg/1ml Oral suspension Oral
Bendroflumethiazide 2.5mg tablets (Genesis Pharmaceuticals Ltd) Bendroflumethiazide 2.5mg Tablet Oral
HYDROCHLOROTHIAZIDE /METOPROLOL TARTRATE 25 MG TAB
AMILORIDE 5MG/HYDROCHLORTHIAZIDE 50MG
AMILORIDE 5MG/HYDROCHLORTHIAZIDE 50MG
Adizem sr 90mg Modified-release capsule (Napp Pharmaceuticals Ltd) Diltiazem hydrochloride 90mg Modified-release capsule Oral

Lasilactone 20mg/50mg capsules (Sanofi) Furosemide/Spironolactone 20mg + 50mg Capsule Oral
Prestim tablets (Meda Pharmaceuticals Ltd) Timolol maleate/Bendroflumethiazide 10mg + 2.5mg Tablet Oral

Piretanide 6mg capsule Piretanide 6mg Capsules Oral
Bumetanide 1mg tablets (DE Pharmaceuticals) Bumetanide 1mg Tablet Oral

Furosemide 40mg tablets (Alliance Healthcare (Distribution) Ltd) Furosemide 40mg Tablet Oral
Co-amilofruse 5mg/40mg tablets (Waymade Healthcare Plc) Amiloride hydrochloride/Furosemide 5mg + 40mg Tablet Oral
Triamterene 50mg / Benzthiazide 25mg capsules Triamterene/Benzthiazide 50mg + 25mg Capsule Oral
Furosemide 20mg Tablet (Celltech Pharma Europe Ltd) Furosemide 20mg Tablet Oral
Hydrochlorothiazide with captopril 12.5mg with 25mg Tablet Captopril/Hydrochlorothiazide 12.5mg + 25mg Tablet Oral
Diuresal 40mg Tablet (Lagap) Furosemide 40mg Tablet Oral
Furosemide 500mg Tablet (Berk Pharmaceuticals Ltd) Furosemide 500mg Tablet Oral
Bumetanide 1mg tablets (A A H Pharmaceuticals Ltd) Bumetanide 1mg Tablet Oral
Amiloride 5.67mg tablets Amiloride Hydrochloride 5.67mg Tablets Oral
Furosemide 500mg tablets Furosemide 500mg Tablet Oral

Indapamide 2.5mg tablets (Boston Healthcare Ltd) Indapamide hemihydrate 2.5mg Tablet Oral
Hydrochlorothiazide with losartan 12.5mg with 50mg Tablet Hydrochlorothiazide/Losartan Potassium 12.5mg + 50mg Tablet Oral
Sotazide Tablet (Bristol-Myers Squibb Pharmaceuticals Ltd) Hydrochlorothiazide/Sotalol Hydrochloride Tablet Oral
Amiloride 2.5mg / hydrochlorothiazide 25mg tablets Amiloride Hydrochloride/Hydrochlorothiazide 2.5mg+25mg Tablets Oral
Furosemide 40mg tablets (A A H Pharmaceuticals Ltd) Furosemide 40mg Tablet Oral
Lasix 40mg tablets (Sanofi) Furosemide 40mg Tablet Oral

Furosemide 20mg tablets (Alliance Healthcare (Distribution) Ltd) Furosemide 20mg Tablet Oral
CHLOROTHIAZIDE SACHETS 60 MG
Torem 5mg tablets (Mylan) Torasemide 5mg Tablet Oral
Inderex 160mg/5mg modified-release capsules (AstraZeneca UK Ltd) Propranolol hydrochloride/Bendroflumethiazide 160mg + 5mg Modified-release capsule Oral
Co-tenidone 50mg/12.5mg tablets (Actavis UK Ltd) Atenolol/Chlortalidone 50mg + 12.5mg Tablet Oral
Tenoretic 100mg/25mg tablets (Dowelhurst Ltd) Atenolol/Chlortalidone 100mg + 25mg Tablet Oral
Lasix 20mg/2ml solution for injection ampoules (Sanofi) Furosemide 10mg/1ml Solution for injection Intravenous/Intramuscular
Hypertane 50 Tablet (Schwarz Pharma Ltd) Amiloride hydrochloride/Hydrochlorothiazide 5mg + 50mg Tablet Oral
Co-amilozide 5mg/50mg tablets (Phoenix Healthcare Distribution Ltd) Amiloride hydrochloride/Hydrochlorothiazide 5mg + 50mg Tablet Oral
Bumetanide 1mg tablets (Phoenix Healthcare Distribution Ltd) Bumetanide 1mg Tablet Oral
Co-amilozide 2.5mg/25mg tablets (Wockhardt UK Ltd) Amiloride hydrochloride/Hydrochlorothiazide 2.5mg + 25mg Tablet Oral
Atenix Co 50 tablets (Ashbourne Pharmaceuticals Ltd) Atenolol/Chlortalidone 50mg + 12.5mg Tablet Oral
Amilmaxco 5mg/50mg tablets (Ashbourne Pharmaceuticals Ltd) Amiloride hydrochloride/Hydrochlorothiazide 5mg + 50mg Tablet Oral

Valsartan 160mg / Hydrochlorothiazide 25mg tablets Valsartan/Hydrochlorothiazide 160mg + 25mg Tablet Oral
Mefruside 25mg Tablet Mefruside 25mg Tablet Oral
Lisinopril 20mg / Hydrochlorothiazide 12.5mg tablets Lisinopril/Hydrochlorothiazide 20mg + 12.5mg Tablet Oral
Bumetanide 2mg/4ml solution for injection ampoules Bumetanide 500microgram/1ml Solution for injection Intramuscular/Intravenous
Chlorothiazide oral solution Chlorothiazide
Furosemide 20mg tablets (DE Pharmaceuticals) Furosemide 20mg Tablet Oral
Centyl 5mg Tablet (Edwin Burgess Ltd) Bendroflumethiazide 5mg Tablet Oral
Losartan 50mg / Hydrochlorothiazide 12.5mg tablets (Ranbaxy (UK) Ltd) Losartan potassium/Hydrochlorothiazide 50mg + 12.5mg Tablet Oral

Co-triamterzide 50mg/25mg tablets Triamterene/Hydrochlorothiazide 50mg + 25mg Tablet Oral
Furosemide 50mg/5ml solution for injection ampoules (Hameln Pharmaceuticals Ltd) Furosemide 10mg/1ml Solution for injection Intravenous/Intramuscular
Amiloride 5mg tablets (Teva UK Ltd) Amiloride hydrochloride 5mg Tablet Oral
Frusid 40mg tablets (Dr Reddy's Laboratories (UK) Ltd) Furosemide 40mg Tablet Oral
Adizem-SR 120mg capsules (Napp Pharmaceuticals Ltd) Diltiazem hydrochloride 120mg Modified-release capsule Oral
Furosemide 50mg/5ml oral solution sugar free Furosemide 10mg/1ml Oral solution Oral
Losartan 100mg / Hydrochlorothiazide 25mg tablets Losartan potassium/Hydrochlorothiazide 100mg + 25mg Tablet Oral

Metoprolol tartrate with chlortalidone Tablet Chlortalidone/Metoprolol Tartrate Tablet Oral
Furosemide 20mg/5ml oral solution sugar free (A A H Pharmaceuticals Ltd) Furosemide 4mg/1ml Oral solution Oral
Zestoretic 20 tablets (Waymade Healthcare Plc) Lisinopril/Hydrochlorothiazide 20mg + 12.5mg Tablet Oral
Olmetec Plus 20mg/25mg tablets (Daiichi Sankyo UK Ltd) Hydrochlorothiazide/Olmesartan medoxomil 25mg + 20mg Tablet Oral
Co-tenidone 100mg/25mg tablets (A A H Pharmaceuticals Ltd) Atenolol/Chlortalidone 100mg + 25mg Tablet Oral

Esidrex 50mg Tablet (Novartis Pharmaceuticals UK Ltd) Hydrochlorothiazide 50mg Tablet Oral
Moducren tablets (Merck Sharp & Dohme Ltd) Timolol maleate/Hydrochlorothiazide/Amiloride hydrochloride 10mg + 25mg + 2.5mg Tablet Oral
Torem 10mg tablets (Mylan) Torasemide 10mg Tablet Oral
Chlortalidone 100mg tablets Chlortalidone 100mg Tablets Oral
Enalapril 20mg / Hydrochlorothiazide 12.5mg tablets (A A H Pharmaceuticals Ltd) Enalapril maleate/Hydrochlorothiazide 20mg + 12.5mg Tablet Oral
Lasix 500mg tablets (Sanofi) Furosemide 500mg Tablet Oral
Lasikal modified-release tablets (Borg Medicare) Potassium chloride/Furosemide 750mg + 20mg Modified-release tablet Oral
Prestim Tablet (ICN Pharmaceuticals France S.A.) Timolol maleate/Bendroflumethiazide 10mg + 2.5mg Tablet Oral
Bendroflumethiazide 5mg tablets (A A H Pharmaceuticals Ltd) Bendroflumethiazide 5mg Tablet Oral
Bendroflumethiazide 5mg tablets (Dr Reddy's Laboratories (UK) Ltd) Bendroflumethiazide 5mg Tablet Oral

Furosemide 10mg/5ml oral suspension Furosemide 2mg/1ml Oral suspension Oral

Indapamide 1.5mg modified-release tablets Indapamide 1.5mg Modified-release tablet Oral
Furosemide 50mg/5ml solution for injection ampoules (Peckforton Pharmaceuticals Ltd) Furosemide 10mg/1ml Solution for injection Intravenous/Intramuscular
Centyl K modified-release tablets (LEO Pharma) Bendroflumethiazide/Potassium chloride 2.5mg + 573mg Modified-release tablet Oral
Furosemide 40mg tablets (Advanz Pharma) Furosemide 40mg Tablet Oral

Sevikar HCT 20mg/5mg/12.5mg tablets (Daiichi Sankyo UK Ltd) Hydrochlorothiazide/Amlodipine besilate/Olmesartan medoxomil 12.5mg + 5mg + 20mg Tablet Oral
Lasix with k Tablet (Hoechst Marion Roussel) Potassium chloride/Furosemide 600mg + 40mg Modified-release tablet Oral
Furosemide 20mg tablets (Bristol Laboratories Ltd) Furosemide 20mg Tablet Oral
Lasix 5mg/5ml oral solution (Borg Medicare) Furosemide 1mg/1ml Oral solution Oral
Chlortalidone 25mg tablets Chlortalidone 25mg Tablet Oral
Chlorothiazide 250mg/5ml oral solution (Special Order) Chlorothiazide 50mg/1ml Oral solution Oral
Indapamide 2.5mg tablets (Niche Generics Ltd) Indapamide hemihydrate 2.5mg Tablet Oral
CYCLOPENTHIAZIDE 250MCG/K 8.1MMOL
Mapemid XL 1.5mg tablets (Teva UK Ltd) Indapamide 1.5mg Modified-release tablet Oral
Adizem xl 180mg Capsule (Napp Pharmaceuticals Ltd) Diltiazem Hydrochloride 180mg Capsule Oral
Natrilix 2.5mg tablets (Servier Laboratories Ltd) Indapamide hemihydrate 2.5mg Tablet Oral
Hydrochlorothiazide 12.5mg tablets Hydrochlorothiazide 12.5mg Tablet Oral
Furosemide 20mg tablets (Wockhardt UK Ltd) Furosemide 20mg Tablet Oral
Amiloride 5mg tablets Amiloride hydrochloride 5mg Tablet Oral
Aprinox 2.5mg tablets (Advanz Pharma) Bendroflumethiazide 2.5mg Tablet Oral
Bumetanide 1mg tablets (Niche Generics Ltd) Bumetanide 1mg Tablet Oral
Indapamide 2.5mg tablets (Zentiva) Indapamide hemihydrate 2.5mg Tablet Oral
Captopril 25mg with Hydrochlorothiazide 12.5mg tablets Captopril/Hydrochlorothiazide 25mg + 12.5mg Tablets Oral
Chlorothiazide 40mg/5ml oral suspension Chlorothiazide 8mg/1ml Oral suspension Oral
Frumil 40mg/5mg tablets (Waymade Healthcare Plc) Amiloride hydrochloride/Furosemide 5mg + 40mg Tablet Oral
Zaroxolyn 5mg tablets (Imported (Canada)) Metolazone 5mg Tablet Oral
Co-flumactone 25mg/25mg tablets Spironolactone/Hydroflumethiazide 25mg + 25mg Tablet Oral
TOTARETIC 50 TAB
Co-amilozide 5mg with 50mg/ml oral solution Amiloride Hydrochloride/Hydrochlorothiazide 5mg + 50mg/5ml Oral Solution Oral
Vasetic Tablet (Shire Pharmaceuticals Ltd) Amiloride hydrochloride/Hydrochlorothiazide 5mg + 50mg Tablet Oral
Indapamide 2.5mg tablets (Alliance Healthcare (Distribution) Ltd) Indapamide hemihydrate 2.5mg Tablet Oral
NAVIDREX
Moduret 25 tablets (Merck Sharp & Dohme Ltd) Amiloride hydrochloride/Hydrochlorothiazide 2.5mg + 25mg Tablet Oral
Indapamide 2.5mg tablets (Teva UK Ltd) Indapamide hemihydrate 2.5mg Tablet Oral

CoAprovel 150mg/12.5mg tablets (Sanofi) Hydrochlorothiazide/Irbesartan 12.5mg + 150mg Tablet Oral
Co-amilofruse 2.5mg/20mg tablets (Teva UK Ltd) Amiloride hydrochloride/Furosemide 2.5mg + 20mg Tablet Oral

Amiloride 5mg Tablet (IVAX Pharmaceuticals UK Ltd) Amiloride hydrochloride 5mg Tablet Oral
Lisinopril 20mg / Hydrochlorothiazide 12.5mg tablets (Almus Pharmaceuticals Ltd) Lisinopril/Hydrochlorothiazide 20mg + 12.5mg Tablet Oral
Cozaar-Comp 50mg/12.5mg tablets (DE Pharmaceuticals) Losartan potassium/Hydrochlorothiazide 50mg + 12.5mg Tablet Oral
Frumil LS 20mg/2.5mg tablets (Sanofi) Amiloride hydrochloride/Furosemide 2.5mg + 20mg Tablet Oral

Natramid 2.5mg Tablet (Trinity Pharmaceuticals Ltd) Indapamide hemihydrate 2.5mg Tablet Oral
Olmesartan medoxomil 40mg / Hydrochlorothiazide 12.5mg tablets Olmesartan medoxomil/Hydrochlorothiazide 40mg + 12.5mg Tablet Oral

Bendroflumethiazide 2.5mg tablets (Crescent Pharma Ltd)Bendroflumethiazide2.5mgTabletOral

Lorvacs XL 1.5mg tablets (Torrent Pharma (UK) Ltd)Indapamide1.5mgModified-release tabletOral
Furosemide 40mg tablets (Sigma Pharmaceuticals Plc)Furosemide40mgTabletOral
Co-zidocapt 25mg/50mg tablets (Tillomed Laboratories Ltd)Hydrochlorothiazide/Captopril25mg + 50mgTabletOral
Furosemide 10mg/ml Injection (Hameln Pharmaceuticals Ltd)Furosemide10mg/mlInjection
Bendroflumethiazide 2.5mg tablets (Phoenix Healthcare Distribution Ltd)Bendroflumethiazide2.5mgTabletOral
Bendroflumethiazide 2.5mg tablets (Sigma Pharmaceuticals Plc)Bendroflumethiazide2.5mgTabletOral
Losartan 100mg / Hydrochlorothiazide 25mg tablets (Alliance Healthcare (Distribution) Ltd)Losartan potassium/Hydrochlorothiazide100mg + 25mgTabletOral
Lisinopril 10mg / Hydrochlorothiazide 12.5mg tablets (A A H Pharmaceuticals Ltd)Lisinopril/Hydrochlorothiazide10mg + 12.5mgTabletOral
Valsartan 160mg / Hydrochlorothiazide 12.5mg tablets (Arrow Generics Ltd)Valsartan/Hydrochlorothiazide160mg + 12.5mgTabletOral
Co-tenidone 100mg/25mg tablets (Medreich Plc)Atenolol/Chlortalidone100mg + 25mgTabletOral
Co-tenidone 50mg/12.5mg tablets (Medreich Plc)Atenolol/Chlortalidone50mg + 12.5mgTabletOral
Losartan 100mg / Hydrochlorothiazide 25mg tablets (Sandoz Ltd)Losartan potassium/Hydrochlorothiazide100mg + 25mgTabletOral
Valsartan 80mg / Hydrochlorothiazide 12.5mg tablets (Arrow Generics Ltd)Valsartan/Hydrochlorothiazide80mg + 12.5mgTabletOral
Losartan 50mg / Hydrochlorothiazide 12.5mg tablets (Sandoz Ltd)Hydrochlorothiazide/Losartan potassium12.5mg + 50mgTabletOral
CoAprovel 300mg/12.5mg tablets (Waymade Healthcare Plc)Irbesartan/Hydrochlorothiazide300mg + 12.5mgTabletOral
CoAprovel 150mg/12.5mg tablets (Waymade Healthcare Plc)Irbesartan/Hydrochlorothiazide150mg + 12.5mgTabletOral
Capozide 25mg/50mg tablets (Waymade Healthcare Plc)Hydrochlorothiazide/Captopril25mg + 50mgTabletOral

Generic Kalten capsulesAtenolol/Amiloride hydrochloride/Hydrochlorothiazide50mg + 2.5mg + 25mgCapsuleOral
Furosemide 20mg tablets (Genesis Pharmaceuticals Ltd)Furosemide20mgTabletOral
Furosemide 40mg tablets (Sovereign Medical Ltd)Furosemide40mgTabletOral

Aspirin

DRUG_CODE_	DESCRIPTION
68752	Aspirin 75mg tablets (Sigma Pharmaceuticals Plc) Aspirin 75mg Tablet Oral
66861	Aspirin 75mg effervescent tablets Aspirin 75mg Effervescent tablet Oral
34434	Aspirin 75mg dispersible tablets (Thornton & Ross Ltd) Aspirin 75mg Dispersible tablet Oral
71663	Aspirin 75mg tablets (Accord Healthcare Ltd) Aspirin 75mg Tablet Oral
4523	ASPIRIN 50 MG CAP
7944	ASPIRIN SOLUBLE 40 MG CAP
54284	Aspirin 75mg dispersible tablets (Almus Pharmaceuticals Ltd) Aspirin 75mg Dispersible tablet Oral
37541	Aspirin 227mg medicated chewing-gum Aspirin 227mg Chewing Gum Oromucosal
31954	Aspirin 75mg dispersible tablets (Teva UK Ltd) Aspirin 75mg Dispersible tablet Oral
59021	Aspirin 75mg gastro-resistant tablets (Bristol Laboratories Ltd) Aspirin 75mg Gastro-resistant tablet Oral
56995	Aspirin 75mg dispersible tablets (Phoenix Healthcare Distribution Ltd) Aspirin 75mg Dispersible tablet Oral
25718	Angettes 75 tablets (Bristol-Myers Squibb Pharmaceuticals Ltd) Aspirin 75mg Tablet Oral
49685	Aspirin 75mg dispersible tablets (Sigma Pharmaceuticals Plc) Aspirin 75mg Dispersible tablet Oral
32036	Aspirin 75mg dispersible tablets (Actavis UK Ltd) Aspirin 75mg Dispersible tablet Oral
18329	Enprin 75mg gastro-resistant tablets (Galpharm International Ltd) Aspirin 75mg Gastro-resistant tablet Oral
34385	Aspirin 75mg Soluble tablet (Co-operative) Aspirin 75mg Dispersible tablet Oral
6696	Micropirin 75mg gastro-resistant tablets (Dexcel-Pharma Ltd) Aspirin 75mg Gastro-resistant tablet Oral
60694	Aspirin 25mg/5ml oral solution Aspirin 5mg/1ml Oral solution Oral
32992	Aspirin 75mg gastro-resistant tablets (Mylan) Aspirin 75mg Gastro-resistant tablet Oral
60777	Aspirin 75mg gastro-resistant tablets (DE Pharmaceuticals) Aspirin 75mg Gastro-resistant tablet Oral
41512	Aspirin 75mg gastro-resistant tablets (Teva UK Ltd) Aspirin 75mg Gastro-resistant tablet Oral
7486	ASPIRIN 37.5 MG TAB
34	Aspirin 75mg gastro-resistant tablets Aspirin 75mg Gastro-resistant tablet Oral
53178	Aspirin 75mg gastro-resistant tablets (Wockhardt UK Ltd) Aspirin 75mg Gastro-resistant tablet Oral
9144	Caprin 75mg gastro-resistant tablets (Wockhardt UK Ltd) Aspirin 75mg Gastro-resistant tablet Oral
42061	ASPIRIN 65 MG SUP
74786	Aspirin 75mg gastro-resistant tablets (Alliance Healthcare (Distribution) Ltd) Aspirin 75mg Gastro-resistant tablet Oral
34485	Aspirin 75mg gastro-resistant tablets (IVAX Pharmaceuticals UK Ltd) Aspirin 75mg Gastro-resistant tablet Oral
7417	ASPIRIN 40 MG TAB
60127	Aspirin 75mg tablets (DE Pharmaceuticals) Aspirin 75mg Tablet Oral
40381	Aspirin 75mg Soluble tablet (C P Pharmaceuticals Ltd) Aspirin 75mg Dispersible tablet Oral
52618	Aspirin 75mg dispersible tablets (Bristol Laboratories Ltd) Aspirin 75mg Dispersible tablet Oral

Aspirin 75mg gastro-resistant tablets (Sterwin Medicines) Aspirin 75mg Gastro-resistant
33293 tablet Oral
67521 Aspirin 15mg/5ml oral suspension Aspirin 3mg/1ml Oral suspension Oral

70549 Danamep 75mg dispersible tablets (Ecogen Europe Ltd) Aspirin 75mg Dispersible tablet Oral
Nu-seals aspirin ec 75mg Gastro-resistant tablet (Eli Lilly and Company Ltd) Aspirin 75mg
2628 Gastro-resistant tablet Oral
74096 Aspirin 50mg/5ml oral suspension Aspirin 10mg/1ml Oral suspension Oral
54997 Aspirin 75mg dispersible tablets (Dowelhurst Ltd) Aspirin 75mg Dispersible tablet Oral
111 ASPIRIN 40 MG CAP
8734 ASPIRIN disp 37.5 MG TAB
8733 JUNIOR ASPIRIN 37.5 MG TAB
79812 Aspirin 75mg tablets (Intrapharm Laboratories Ltd) Aspirin 75mg Tablet Oral
50949 Aspirin 75mg tablets (A A H Pharmaceuticals Ltd) Aspirin 75mg Tablet Oral
74302 Aspirin 75mg/5ml oral suspension Aspirin 15mg/1ml Oral suspension Oral
Boots Aspirin 75mg gastro-resistant tablets (The Boots Company Plc) Aspirin 75mg Gastro-
80356 resistant tablet Oral
Aspirin 75mg dispersible tablets (Kent Pharmaceuticals Ltd) Aspirin 75mg Dispersible tablet
33676 Oral

57057 Aspirin 75mg dispersible tablets (Wockhardt UK Ltd) Aspirin 75mg Dispersible tablet Oral
15397 ASPIRIN SOLUBLE 50 MG TAB
71192 Aspirin 75mg tablets (Kent Pharmaceuticals Ltd) Aspirin 75mg Tablet Oral
67858 Aspirin 25mg capsules Aspirin 25mg Capsule Oral
Aspirin 75mg dispersible tablets (Alliance Healthcare (Distribution) Ltd) Aspirin 75mg
49060 Dispersible tablet Oral
Aspirin 75mg Dispersible tablet (Numark Management Ltd) Aspirin 75mg Dispersible tablet
80481 Oral
34942 Aspirin 75mg Dispersible tablet (Nucare Plc) Aspirin 75mg Dispersible tablet Oral
Aspirin 75mg dispersible tablets (IVAX Pharmaceuticals UK Ltd) Aspirin 75mg Dispersible
31953 tablet Oral
Aspirin 75mg gastro-resistant tablets (Phoenix Healthcare Distribution Ltd) Aspirin 75mg
66563 Gastro-resistant tablet Oral
Solprin 75mg Tablet (Reckitt Benckiser Healthcare (UK) Ltd) Aspirin 75mg Dispersible tablet
22618 Oral
80662 Aspirin 75mg tablets (Medreich Plc) Aspirin 75mg Tablet Oral
383 ASPIRIN 60 MG TAB
54430 Aspirin 75mg tablets (Alliance Healthcare (Distribution) Ltd) Aspirin 75mg Tablet Oral
48021 Aspirin 75mg Tablet (Hillcross Pharmaceuticals Ltd) Aspirin 75mg Tablet Oral
Aspirin 75mg dispersible tablets (A A H Pharmaceuticals Ltd) Aspirin 75mg Dispersible tablet
33656 Oral
Micropirin 75mg Gastro-resistant tablet (Ratiopharm UK Ltd) Aspirin 75mg Gastro-resistant
19189 tablet Oral
66546 Aspirin 75mg dispersible tablets (Numark Ltd) Aspirin 75mg Dispersible tablet Oral
16 Aspirin 75mg tablets Aspirin 75mg Tablet Oral

66345 Aspirin 75mg dispersible tablets (DE Pharmaceuticals) Aspirin 75mg Dispersible tablet Oral
Aspirin 75mg gastro-resistant tablets (Almus Pharmaceuticals Ltd) Aspirin 75mg Gastro-
43709 resistant tablet Oral

54565	Aspirin 75mg dispersible tablets (Lloyds Pharmacy Ltd) Aspirin 75mg Dispersible tablet Oral
80766	Aspirin 75mg dispersible tablets (M & A Pharmachem Ltd) Aspirin 75mg Dispersible tablet Oral
33320	Aspirin 75mg Dispersible tablet (Sovereign Medical Ltd) Aspirin 75mg Dispersible tablet Oral
25335	PostMI 75 EC tablets (Ashbourne Pharmaceuticals Ltd) Aspirin 75mg Gastro-resistant tablet Oral
59728	Aspirin 75mg tablets (Alissa Healthcare Research Ltd) Aspirin 75mg Tablet Oral
56996	Aspirin 75mg dispersible tablets (Waymade Healthcare Plc) Aspirin 75mg Dispersible tablet Oral
1486	ASPIRIN 75 MG SUP
31938	Aspirin 75mg gastro-resistant tablets (Sandoz Ltd) Aspirin 75mg Gastro-resistant tablet Oral
47992	Aspirin 75mg gastro-resistant tablets (A A H Pharmaceuticals Ltd) Aspirin 75mg Gastro-resistant tablet Oral
56883	Aspirin 75mg tablets (Waymade Healthcare Plc) Aspirin 75mg Tablet Oral
20206	ASPIRIN 50 MG SUP
6006	Nu-Seals 75 gastro-resistant tablets (Alliance Pharmaceuticals Ltd) Aspirin 75mg Gastro-resistant tablet Oral
34797	Aspirin 75mg gastro-resistant tablets (Actavis UK Ltd) Aspirin 75mg Gastro-resistant tablet Oral
50926	Aspirin 75mg dispersible tablets (The Boots Company Plc) Aspirin 75mg Dispersible tablet Oral
48974	Aspirin 75mg tablets (Phoenix Healthcare Distribution Ltd) Aspirin 75mg Tablet Oral
34611	Aspirin 75mg gastro-resistant tablets (C P Pharmaceuticals Ltd) Aspirin 75mg Gastro-resistant tablet Oral
31870	Aspirin 320mg tablets Aspirin 320mg Tablets Oral
31211	Aspirin 75mg Dispersible tablet (A A H Pharmaceuticals Ltd) Aspirin 75mg Dispersible tablet Oral
23495	ASPIRIN
71676	Aspirin 75mg dispersible tablets (Mawdsley-Brooks & Company Ltd) Aspirin 75mg Dispersible tablet Oral
59253	Aspirin 75mg gastro-resistant tablets (Waymade Healthcare Plc) Aspirin 75mg Gastro-resistant tablet Oral
3	Aspirin 75mg dispersible tablets Aspirin 75mg Dispersible tablet Oral
11941	ASPIRIN SACHETS 30 MG
23878	Nu-seals cardio ec 75mg Gastro-resistant tablet (Genus Pharmaceuticals Ltd) Aspirin 75mg Gastro-resistant tablet Oral
216	ASPIRIN 70 MG TAB
31956	Aspirin 75mg gastro-resistant tablets (Kent Pharmaceuticals Ltd) Aspirin 75mg Gastro-resistant tablet Oral
59791	Aspirin 75mg dispersible tablets (Aspar Pharmaceuticals Ltd) Aspirin 75mg Dispersible tablet Oral
47937	Aspirin 75mg dispersible tablets (Wockhardt UK Ltd) Aspirin 75mg Dispersible tablet Oral
73994	Aspirin 75mg gastro-resistant tablets (Greenfield Pharmaceuticals Ltd) Aspirin 75mg Gastro-resistant tablet Oral
51561	Aspirin 75mg gastro-resistant tablets (Zanza Specials International Ltd) Aspirin 75mg Gastro-resistant tablet Oral

60693 Aspirin 15mg/5ml oral solution Aspirin 3mg/1ml Oral solution Oral

45643 Aspirin 75mg Soluble tablet (Celltech Pharma Europe Ltd) Aspirin 75mg Dispersible tablet Oral
PostMI 75 dispersible tablets (Ashbourne Pharmaceuticals Ltd) Aspirin 75mg Dispersible
23593 tablet Oral

34796 Aspirin 75mg Gastro-resistant tablet (Galen Ltd) Aspirin 75mg Gastro-resistant tablet Oral
Aspirin 75mg gastro-resistant tablets (Lloyds Pharmacy Ltd)Aspirin75mgGastro-resistant
81756 tabletOral

Hypertension

READ_CODE	DESCRIPTION
G2...00	Hypertensive disease
G20..11	High blood pressure
G20..00	Essential hypertension
G201.00	Benign essential hypertension
G20z.11	Hypertension NOS
G202.00	Systolic hypertension
G22..00	Hypertensive renal disease
G2z..00	Hypertensive disease NOS
G24..00	Secondary hypertension
G2...11	BP - hypertensive disease
G21z011	Cardiomegaly - hypertensive
G20z.00	Essential hypertension NOS
G22z.00	Hypertensive renal disease NOS
G200.00	Malignant essential hypertension
G24z.00	Secondary hypertension NOS
G21zz00	Hypertensive heart disease NOS
G21..00	Hypertensive heart disease
G22..11	Nephrosclerosis
G2y..00	Other specified hypertensive disease
G232.00	Hypertensive heart&renal dis wth (congestive) heart failure
G241000	Secondary benign renovascular hypertension
G233.00	Hypertensive heart and renal disease with renal failure
G22z.11	Renal hypertension
G24z100	Hypertension secondary to drug
G24z000	Secondary renovascular hypertension NOS
G21z.00	Hypertensive heart disease NOS
G240.00	Secondary malignant hypertension
G222.00	Hypertensive renal disease with renal failure
G244.00	Hypertension secondary to endocrine disorders
G220.00	Malignant hypertensive renal disease
G24zz00	Secondary hypertension NOS
G221.00	Benign hypertensive renal disease
G210.00	Malignant hypertensive heart disease
G241z00	Secondary benign hypertension NOS
G211100	Benign hypertensive heart disease with CCF
G211.00	Benign hypertensive heart disease
G241.00	Secondary benign hypertension
G234.00	Hyperten heart&renal dis+both(congestv)heart and renal fail
G240000	Secondary malignant renovascular hypertension
G21z000	Hypertensive heart disease NOS without CCF
G211000	Benign hypertensive heart disease without CCF
G21z100	Hypertensive heart disease NOS with CCF
G231.00	Benign hypertensive heart and renal disease
G23..00	Hypertensive heart and renal disease
G230.00	Malignant hypertensive heart and renal disease
G23z.00	Hypertensive heart and renal disease NOS
Gyu2.00	[X]Hypertensive diseases
G210100	Malignant hypertensive heart disease with CCF

G240z00	Secondary malignant hypertension NOS
G203.00	Diastolic hypertension
G210000	Malignant hypertensive heart disease without CCF
Gyu2100	[X]Hypertension secondary to other renal disorders
Gyu2000	[X]Other secondary hypertension
G210z00	Malignant hypertensive heart disease NOS
G28..00	Stage 2 hypertension (NICE - Nat Ins for Hth Clin Excl 2011)
G25..11	Stage 1 hypertension
G25..00	Stage 1 hypertension (NICE - Nat Ins for Hth Clin Excl 2011)
G27..00	Hypertension resistant to drug therapy
G26..11	Severe hypertension
G211z00	Benign hypertensive heart disease NOS
G26..00	Severe hypertension (Nat Inst for Health Clinical Ex 2011)
G20..12	Primary hypertension
G250.00	Stage 1 hyperten (NICE 2011) without evidnce end organ damge
G251.00	Stage 1 hyperten (NICE 2011) with evidnce end organ damge

Type 1 Diabetes

READ_CODE	DESCRIPTION
C100011	Insulin dependent diabetes mellitus
C10E.00	Type 1 diabetes mellitus
C108.00	Insulin dependent diabetes mellitus
C108700	Insulin dependent diabetes mellitus with retinopathy
C108800	Insulin dependent diabetes mellitus - poor control
C10ED00	Type 1 diabetes mellitus with nephropathy
C10EM00	Type 1 diabetes mellitus with ketoacidosis
C10E.11	Type I diabetes mellitus
C108F11	Type I diabetes mellitus with diabetic cataract
C108.12	Type 1 diabetes mellitus
C108J12	Type 1 diabetes mellitus with neuropathic arthropathy
C10E700	Type 1 diabetes mellitus with retinopathy
C108.11	IDDM-Insulin dependent diabetes mellitus
C10EH00	Type 1 diabetes mellitus with arthropathy
C10E500	Type 1 diabetes mellitus with ulcer
C108012	Type 1 diabetes mellitus with renal complications
C10EP00	Type 1 diabetes mellitus with exudative maculopathy
C108.13	Type I diabetes mellitus
C100000	Diabetes mellitus, juvenile type, no mention of complication
C108B00	Insulin dependent diabetes mellitus with mononeuropathy
C108400	Unstable insulin dependent diabetes mellitus
C10EL00	Type 1 diabetes mellitus with persistent microalbuminuria
C10EK00	Type 1 diabetes mellitus with persistent proteinuria
C108900	Insulin dependent diabetes maturity onset
C10E800	Type 1 diabetes mellitus - poor control
C108711	Type I diabetes mellitus with retinopathy
C10EE00	Type 1 diabetes mellitus with hypoglycaemic coma
C108J00	Insulin dependent diab mell with neuropathic arthropathy
C102000	Diabetes mellitus, juvenile type, with hyperosmolar coma
C10E900	Type 1 diabetes mellitus maturity onset
C10EN00	Type 1 diabetes mellitus with ketoacidotic coma
C108712	Type 1 diabetes mellitus with retinopathy
C108C00	Insulin dependent diabetes mellitus with polyneuropathy
C103000	Diabetes mellitus, juvenile type, with ketoacidotic coma
C108E11	Type I diabetes mellitus with hypoglycaemic coma
C10E200	Type 1 diabetes mellitus with neurological complications
C10E400	Unstable type 1 diabetes mellitus
C108F00	Insulin dependent diabetes mellitus with diabetic cataract
C108E00	Insulin dependent diabetes mellitus with hypoglycaemic coma
C108500	Insulin dependent diabetes mellitus with ulcer
C10E312	Insulin dependent diabetes mellitus with multiple complicat
C108812	Type 1 diabetes mellitus - poor control
C10EC00	Type 1 diabetes mellitus with polyneuropathy
C108811	Type I diabetes mellitus - poor control
C108000	Insulin-dependent diabetes mellitus with renal complications
C10E000	Type 1 diabetes mellitus with renal complications
C10E100	Type 1 diabetes mellitus with ophthalmic complications
C10E300	Type 1 diabetes mellitus with multiple complications

C108211	Type I diabetes mellitus with neurological complications
C108100	Insulin-dependent diabetes mellitus with ophthalmic comps
C10EF00	Type 1 diabetes mellitus with diabetic cataract
C10E411	Unstable type I diabetes mellitus
C10E.12	Insulin dependent diabetes mellitus
C108511	Type I diabetes mellitus with ulcer
C108300	Insulin dependent diabetes mellitus with multiple complicatn
C108200	Insulin-dependent diabetes mellitus with neurological comps
C101000	Diabetes mellitus, juvenile type, with ketoacidosis
C10EJ00	Type 1 diabetes mellitus with neuropathic arthropathy
C10E412	Unstable insulin dependent diabetes mellitus
C10EQ00	Type 1 diabetes mellitus with gastroparesis
C108A00	Insulin-dependent diabetes without complication
C108D00	Insulin dependent diabetes mellitus with nephropathy
C108411	Unstable type I diabetes mellitus
C108J11	Type I diabetes mellitus with neuropathic arthropathy
C108600	Insulin dependent diabetes mellitus with gangrene
C108011	Type I diabetes mellitus with renal complications
C108212	Type 1 diabetes mellitus with neurological complications
C10EM11	Type I diabetes mellitus with ketoacidosis
C108H11	Type I diabetes mellitus with arthropathy
C10EA11	Type I diabetes mellitus without complication
C108911	Type I diabetes mellitus maturity onset
C108G00	Insulin dependent diab mell with peripheral angiopathy
C108H00	Insulin dependent diabetes mellitus with arthropathy
C10EN11	Type I diabetes mellitus with ketoacidotic coma
C108D11	Type I diabetes mellitus with nephropathy
C106000	Diabetes mellitus, juvenile, + neurological manifestation
C10EB00	Type 1 diabetes mellitus with mononeuropathy
C108512	Type 1 diabetes mellitus with ulcer
C10z000	Diabetes mellitus, juvenile type, + unspecified complication
C107300	IDDM with peripheral circulatory disorder
C10EA00	Type 1 diabetes mellitus without complication
C105000	Diabetes mellitus, juvenile type, + ophthalmic manifestation
C10E600	Type 1 diabetes mellitus with gangrene
C107000	Diabetes mellitus, juvenile +peripheral circulatory disorder
C108E12	Type 1 diabetes mellitus with hypoglycaemic coma
C10E812	Insulin dependent diabetes mellitus - poor control
C10E311	Type I diabetes mellitus with multiple complications
C10EC11	Type I diabetes mellitus with polyneuropathy
C10EG00	Type 1 diabetes mellitus with peripheral angiopathy
C10E712	Insulin dependent diabetes mellitus with retinopathy
C10E511	Type I diabetes mellitus with ulcer
C104000	Diabetes mellitus, juvenile type, with renal manifestation
C10E711	Type I diabetes mellitus with retinopathy
C108A11	Type I diabetes mellitus without complication
C10E911	Type I diabetes mellitus maturity onset
C108912	Type 1 diabetes mellitus maturity onset
C108412	Unstable type 1 diabetes mellitus
C10E912	Insulin dependent diabetes maturity onset

C10EP11	Type I diabetes mellitus with exudative maculopathy
C10E112	Insulin-dependent diabetes mellitus with ophthalmic comps
C10C.12	Maturity onset diabetes in youth type 1
C10E512	Insulin dependent diabetes mellitus with ulcer
C108B11	Type I diabetes mellitus with mononeuropathy
C10E111	Type I diabetes mellitus with ophthalmic complications
C10EE12	Insulin dependent diabetes mellitus with hypoglycaemic coma
C10EA12	Insulin-dependent diabetes without complication
C10EF12	Insulin dependent diabetes mellitus with diabetic cataract
C10EC12	Insulin dependent diabetes mellitus with polyneuropathy
C10E212	Insulin-dependent diabetes mellitus with neurological comps
C10E611	Type I diabetes mellitus with gangrene
C10ED12	Insulin dependent diabetes mellitus with nephropathy
C10EL11	Type I diabetes mellitus with persistent microalbuminuria
C108112	Type 1 diabetes mellitus with ophthalmic complications
C10E012	Insulin-dependent diabetes mellitus with renal complications
C10E811	Type I diabetes mellitus - poor control
C108311	Type I diabetes mellitus with multiple complications
C10P000	Type I diabetes mellitus in remission
C10EQ11	Type I diabetes mellitus with gastroparesis
C10E612	Insulin dependent diabetes mellitus with gangrene
C10P011	Type 1 diabetes mellitus in remission
C10E011	Type I diabetes mellitus with renal complications
C108F12	Type 1 diabetes mellitus with diabetic cataract
C10y000	Diabetes mellitus, juvenile, + other specified manifestation
C108A12	Type 1 diabetes mellitus without complication
C108D12	Type 1 diabetes mellitus with nephropathy
C10ED11	Type I diabetes mellitus with nephropathy
C108C11	Type I diabetes mellitus with polyneuropathy
C108612	Type 1 diabetes mellitus with gangrene

Type 2 Diabetes

READ_CODE	DESCRIPTION
C100112	Non-insulin dependent diabetes mellitus
C10F.00	Type 2 diabetes mellitus
C10FJ00	Insulin treated Type 2 diabetes mellitus
C109.00	Non-insulin dependent diabetes mellitus
C109.11	NIDDM - Non-insulin dependent diabetes mellitus
C109700	Non-insulin dependent diabetes mellitus - poor control
C10FC00	Type 2 diabetes mellitus with nephropathy
C10F500	Type 2 diabetes mellitus with gangrene
C100100	Diabetes mellitus, adult onset, no mention of complication
C109600	Non-insulin-dependent diabetes mellitus with retinopathy
C109.12	Type 2 diabetes mellitus
C109G11	Type II diabetes mellitus with arthropathy
C109012	Type 2 diabetes mellitus with renal complications
C109.13	Type II diabetes mellitus
C109J12	Insulin treated Type II diabetes mellitus
C109J00	Insulin treated Type 2 diabetes mellitus
C10FM00	Type 2 diabetes mellitus with persistent microalbuminuria
C10FB00	Type 2 diabetes mellitus with polyneuropathy
C10F600	Type 2 diabetes mellitus with retinopathy
C10F000	Type 2 diabetes mellitus with renal complications
C10F.11	Type II diabetes mellitus
C109711	Type II diabetes mellitus - poor control
C109G00	Non-insulin dependent diabetes mellitus with arthropathy
C109C12	Type 2 diabetes mellitus with nephropathy
C10FQ00	Type 2 diabetes mellitus with exudative maculopathy
C10F700	Type 2 diabetes mellitus - poor control
C10FL00	Type 2 diabetes mellitus with persistent proteinuria
C109900	Non-insulin-dependent diabetes mellitus without complication
C10FN00	Type 2 diabetes mellitus with ketoacidosis
C10F200	Type 2 diabetes mellitus with neurological complications
C10FK00	Hyperosmolar non-ketotic state in type 2 diabetes mellitus
C109400	Non-insulin dependent diabetes mellitus with ulcer
C104100	Diabetes mellitus, adult onset, with renal manifestation
C10FH00	Type 2 diabetes mellitus with neuropathic arthropathy
C109K00	Hyperosmolar non-ketotic state in type 2 diabetes mellitus
C10D.00	Diabetes mellitus autosomal dominant type 2
C109J11	Insulin treated non-insulin dependent diabetes mellitus
C10FF00	Type 2 diabetes mellitus with peripheral angiopathy
C106100	Diabetes mellitus, adult onset, + neurological manifestation
C10F811	Metabolic syndrome X
C109500	Non-insulin dependent diabetes mellitus with gangrene
C109H00	Non-insulin dependent d m with neuropathic arthropathy
C105100	Diabetes mellitus, adult onset, + ophthalmic manifestation
C109612	Type 2 diabetes mellitus with retinopathy
C102100	Diabetes mellitus, adult onset, with hyperosmolar coma
C10F311	Type II diabetes mellitus with multiple complications
C109D00	Non-insulin dependent diabetes mellitus with hypoglyca coma
C109E12	Type 2 diabetes mellitus with diabetic cataract

C10FE00	Type 2 diabetes mellitus with diabetic cataract
C109B00	Non-insulin dependent diabetes mellitus with polyneuropathy
C109712	Type 2 diabetes mellitus - poor control
C109212	Type 2 diabetes mellitus with neurological complications
C109512	Type 2 diabetes mellitus with gangrene
C10FD00	Type 2 diabetes mellitus with hypoglycaemic coma
C10F711	Type II diabetes mellitus - poor control
C10F100	Type 2 diabetes mellitus with ophthalmic complications
C109B11	Type II diabetes mellitus with polyneuropathy
C109H11	Type II diabetes mellitus with neuropathic arthropathy
C10F900	Type 2 diabetes mellitus without complication
C109E11	Type II diabetes mellitus with diabetic cataract
C10F400	Type 2 diabetes mellitus with ulcer
C10F611	Type II diabetes mellitus with retinopathy
C109G12	Type 2 diabetes mellitus with arthropathy
C109011	Type II diabetes mellitus with renal complications
C109100	Non-insulin-dependent diabetes mellitus with ophthalm comps
C10FB11	Type II diabetes mellitus with polyneuropathy
C109A11	Type II diabetes mellitus with mononeuropathy
C10FP00	Type 2 diabetes mellitus with ketoacidotic coma
C109000	Non-insulin-dependent diabetes mellitus with renal comps
C10F911	Type II diabetes mellitus without complication
C109F00	Non-insulin-dependent d m with peripheral angiopath
C101100	Diabetes mellitus, adult onset, with ketoacidosis
C109F11	Type II diabetes mellitus with peripheral angiopathy
C109411	Type II diabetes mellitus with ulcer
C109200	Non-insulin-dependent diabetes mellitus with neuro comps
C109D11	Type II diabetes mellitus with hypoglycaemic coma
C107400	NIDDM with peripheral circulatory disorder
C10F011	Type II diabetes mellitus with renal complications
C109611	Type II diabetes mellitus with retinopathy
C10FG00	Type 2 diabetes mellitus with arthropathy
C109C00	Non-insulin dependent diabetes mellitus with nephropathy
C109111	Type II diabetes mellitus with ophthalmic complications
C10D.11	Maturity onset diabetes in youth type 2
C109F12	Type 2 diabetes mellitus with peripheral angiopathy
C10FL11	Type II diabetes mellitus with persistent proteinuria
C109D12	Type 2 diabetes mellitus with hypoglycaemic coma
C109511	Type II diabetes mellitus with gangrene
C109300	Non-insulin-dependent diabetes mellitus with multiple comps
C10FA00	Type 2 diabetes mellitus with mononeuropathy
C107100	Diabetes mellitus, adult, + peripheral circulatory disorder
C10y100	Diabetes mellitus, adult, + other specified manifestation
C10FR00	Type 2 diabetes mellitus with gastroparesis
C10z100	Diabetes mellitus, adult onset, + unspecified complication
C109C11	Type II diabetes mellitus with nephropathy
C10FJ11	Insulin treated Type II diabetes mellitus
C10F300	Type 2 diabetes mellitus with multiple complications
C109412	Type 2 diabetes mellitus with ulcer
C109H12	Type 2 diabetes mellitus with neuropathic arthropathy

C109211	Type II diabetes mellitus with neurological complications
C103100	Diabetes mellitus, adult onset, with ketoacidotic coma
C109E00	Non-insulin depend diabetes mellitus with diabetic cataract
C109112	Type 2 diabetes mellitus with ophthalmic complications
C109A00	Non-insulin dependent diabetes mellitus with mononeuropathy
C10FM11	Type II diabetes mellitus with persistent microalbuminuria
C10F411	Type II diabetes mellitus with ulcer
C10FE11	Type II diabetes mellitus with diabetic cataract
C10FA11	Type II diabetes mellitus with mononeuropathy
C10F211	Type II diabetes mellitus with neurological complications
C10FD11	Type II diabetes mellitus with hypoglycaemic coma
C10F111	Type II diabetes mellitus with ophthalmic complications
C10FC11	Type II diabetes mellitus with nephropathy
C10FG11	Type II diabetes mellitus with arthropathy
C10F511	Type II diabetes mellitus with gangrene
C10FF11	Type II diabetes mellitus with peripheral angiopathy
C109912	Type 2 diabetes mellitus without complication
C10FP11	Type II diabetes mellitus with ketoacidotic coma
C10FN11	Type II diabetes mellitus with ketoacidosis
C10FK11	Hyperosmolar non-ketotic state in type II diabetes mellitus
C10P100	Type II diabetes mellitus in remission
C109312	Type 2 diabetes mellitus with multiple complications
C109911	Type II diabetes mellitus without complication
C10FH11	Type II diabetes mellitus with neuropathic arthropathy
C109B12	Type 2 diabetes mellitus with polyneuropathy
C10P111	Type 2 diabetes mellitus in remission
C10FQ11	Type II diabetes mellitus with exudative maculopathy
C109311	Type II diabetes mellitus with multiple complications
C10FR11	Type II diabetes mellitus with gastroparesis

Chronic Kidney Disease stages 3-5

READ_CODE	DESCRIPTION
K050.00	End stage renal failure
1Z13.00	Chronic kidney disease stage 4
1Z12.00	Chronic kidney disease stage 3
1Z14.00	Chronic kidney disease stage 5
K05..12	End stage renal failure
1Z1B.00	Chronic kidney disease stage 3 with proteinuria
1Z15.00	Chronic kidney disease stage 3A
1Z1H.00	Chronic kidney disease stage 4 with proteinuria
1Z1C.00	Chronic kidney disease stage 3 without proteinuria
1Z1B.11	CKD stage 3 with proteinuria
1Z1E.00	Chronic kidney disease stage 3A without proteinuria
1Z1E.11	CKD stage 3A without proteinuria
1Z1G.00	Chronic kidney disease stage 3B without proteinuria
1Z1F.00	Chronic kidney disease stage 3B with proteinuria
1Z16.00	Chronic kidney disease stage 3B
1Z1F.11	CKD stage 3B with proteinuria
1Z1C.11	CKD stage 3 without proteinuria
1Z1L.00	Chronic kidney disease stage 5 without proteinuria
1Z1J.00	Chronic kidney disease stage 4 without proteinuria
1Z1D.00	Chronic kidney disease stage 3A with proteinuria
1Z1K.00	Chronic kidney disease stage 5 with proteinuria
1Z1D.11	CKD stage 3A with proteinuria
1Z1J.11	CKD stage 4 without proteinuria
1Z1L.11	CKD stage 5 without proteinuria
1Z1K.11	CKD stage 5 with proteinuria
1Z1H.11	CKD stage 4 with proteinuria
1Z1G.11	CKD stage 3B without proteinuria
K053.00	Chronic kidney disease stage 3
K054.00	Chronic kidney disease stage 4
K055.00	Chronic kidney disease stage 5
1Z1Y.00	CKD with GFR category G3b & albuminuria category A2
1Z1T.00	CKD with GFR category G3a & albuminuria category A1
1Z1V.00	CKD with GFR category G3a & albuminuria category A2
1Z1b.00	CKD with GFR category G4 & albuminuria category A2
1Z1W.00	CKD with GFR category G3a & albuminuria category A3
1Z1X.00	CKD with GFR category G3b & albuminuria category A1
1Z1a.00	CKD with GFR category G4 & albuminuria category A1
1Z1e.00	CKD with GFR category G5 & albuminuria category A2
1Z1Z.00	CKD with GFR category G3b & albuminuria category A3
1Z1d.00	CKD with GFR category G5 & albuminuria category A1
1Z1f.00	CKD with GFR category G5 & albuminuria category A3
1Z1c.00	CKD with GFR category G4 & albuminuria category A3

Heart failure

READ_CODE	DESCRIPTION
G580.00	Congestive heart failure
G581.00	Left ventricular failure
G58..11	Cardiac failure
G58..00	Heart failure
G580.11	Congestive cardiac failure
G58z.00	Heart failure NOS
G554000	Congestive cardiomyopathy
G581000	Acute left ventricular failure
G581.13	Impaired left ventricular function
G580.14	Biventricular failure
1O1..00	Heart failure confirmed
G580300	Compensated cardiac failure
662T.00	Congestive heart failure monitoring
G58z.11	Weak heart
662g.00	New York Heart Association classification - class II
G58z.12	Cardiac failure NOS
8HBE.00	Heart failure follow-up
662f.00	New York Heart Association classification - class I
662h.00	New York Heart Association classification - class III
G232.00	Hypertensive heart&renal dis wth (congestive) heart failure
G581.11	Asthma - cardiac
G580000	Acute congestive heart failure
8B29.00	Cardiac failure therapy
G580200	Decompensated cardiac failure
G582.00	Acute heart failure
662W.00	Heart failure annual review
G580100	Chronic congestive heart failure
8H2S.00	Admit heart failure emergency
G581.12	Pulmonary oedema - acute
662i.00	New York Heart Association classification - class IV
G234.00	Hyperten heart&renal dis+both(congestv)heart and renal fail
SP11200	Cardiorespiratory failure as a complication of care
SP11111	Heart failure as a complication of care
662p.00	Heart failure 6 month review
G580400	Congestive heart failure due to valvular disease
G5y4z00	Post cardiac operation heart failure NOS
G583.11	HFNEF - heart failure with normal ejection fraction
G583.00	Heart failure with normal ejection fraction
679W100	Education about deteriorating heart failure
661M500	Heart failure self-management plan agreed
8HTL000	Referral to rapid access heart failure clinic
G583.12	Heart failure with preserved ejection fraction
2JZ..00	On optimal heart failure therapy
661N500	Heart failure self-management plan review

READ_CODE	DESCRIPTION
G3...00	Ischaemic heart disease
G30..00	Acute myocardial infarction
G580.00	Congestive heart failure
G65..00	Transient cerebral ischaemia
G64..12	Infarction - cerebral
G581.00	Left ventricular failure
G30..14	Heart attack
G58..11	Cardiac failure
G66..11	CVA unspecified
G340.12	Coronary artery disease
G33z300	Angina on effort
G33..00	Angina pectoris
G311.13	Unstable angina
G65..12	Transient ischaemic attack
G66..00	Stroke and cerebrovascular accident unspecified
G73z000	Intermittent claudication
G340.11	Triple vessel disease of the heart
G3z..00	Ischaemic heart disease NOS
G30..15	MI - acute myocardial infarction
G308.00	Inferior myocardial infarction NOS
G71..00	Aortic aneurysm
G60..00	Subarachnoid haemorrhage
G3...13	IHD - Ischaemic heart disease
G73..12	Ischaemia of legs
G714.00	Abdominal aortic aneurysm without mention of rupture
G65z.00	Transient cerebral ischaemia NOS
G58..00	Heart failure
G341000	Ventricular cardiac aneurysm
G631.11	Stenosis, carotid artery
G65..13	Vertebro-basilar insufficiency
G6...00	Cerebrovascular disease
G30..12	Coronary thrombosis
G634.00	Carotid artery stenosis
G73zz00	Peripheral vascular disease NOS
G580.11	Congestive cardiac failure
G65..11	Drop attack
G64z.00	Cerebral infarction NOS
G73z.00	Peripheral vascular disease NOS
G61z.00	Intracerebral haemorrhage NOS
G72z.00	Aneurysm NOS
G307.00	Acute subendocardial infarction
G672.00	Hypertensive encephalopathy
G340000	Single coronary vessel disease
G32..00	Old myocardial infarction
G58z.00	Heart failure NOS
G631.12	Thrombosis, carotid artery
G631.00	Carotid artery occlusion
G621.00	Subdural haemorrhage - nontraumatic
G74..00	Arterial embolism and thrombosis

G673.00	Cerebral aneurysm, nonruptured
G311.11	Crescendo angina
G61..00	Intracerebral haemorrhage
G554000	Congestive cardiomyopathy
G670.11	Precerebral atherosclerosis
G64z111	Lateral medullary syndrome
G340100	Double coronary vessel disease
G581000	Acute left ventricular failure
G650.11	Insufficiency - basilar artery
G64..11	CVA - cerebral artery occlusion
G301.00	Other specified anterior myocardial infarction
G340.00	Coronary atherosclerosis
G64z.12	Cerebellar infarction
G73..11	Peripheral ischaemic vascular disease
G581.13	Impaired left ventricular function
G73..00	Other peripheral vascular disease
G66..13	CVA - Cerebrovascular accident unspecified
G64..13	Stroke due to cerebral arterial occlusion
G68X.00	Sequelae of stroke,not specfd as h'morrhage or infarction
G66..12	Stroke unspecified
G341.00	Aneurysm of heart
G655.00	Transient global amnesia
G723000	Aneurysm of femoral artery
G73..13	Peripheral ischaemia
G73z011	Claudication
G71z.00	Aortic aneurysm NOS
G61..11	CVA - cerebrovascular accid due to intracerebral haemorrhage
G343.00	Ischaemic cardiomyopathy
G311100	Unstable angina
G76z100	Femoral artery occlusion
G33z200	Syncope anginosa
G667.00	Left sided CVA
G614.00	Pontine haemorrhage
G663.00	Brain stem stroke syndrome
G37..00	Cardiac syndrome X
G64..00	Cerebral arterial occlusion
G302.00	Acute inferolateral infarction
G74..11	Arterial embolus and thrombosis
G31y000	Acute coronary insufficiency
G74..12	Thrombosis - arterial
G31y.00	Other acute and subacute ischaemic heart disease
G72..00	Other aneurysm
G307000	Acute non-Q wave infarction
G580.14	Biventricular failure
G76z200	Popliteal artery occlusion
G33z500	Post infarct angina
G604.00	Subarachnoid haemorrhage from posterior communicating artery
G718.00	Leaking abdominal aortic aneurysm
1O1..00	Heart failure confirmed
G678.00	Cereb autosom dominant arteriop subcort infarcts leukoenceph

G64z200	Left sided cerebral infarction
G6z..00	Cerebrovascular disease NOS
G674000	Cerebral amyloid angiopathy
G64z300	Right sided cerebral infarction
G307100	Acute non-ST segment elevation myocardial infarction
G656.00	Vertebrobasilar insufficiency
G331.11	Variant angina pectoris
G670.00	Cerebral atherosclerosis
G580300	Compensated cardiac failure
G715000	Thoracoabdominal aortic aneurysm, ruptured
G311500	Acute coronary syndrome
G300.00	Acute anterolateral infarction
G30X000	Acute ST segment elevation myocardial infarction
662T.00	Congestive heart failure monitoring
G671z00	Generalised ischaemic cerebrovascular disease NOS
G58z.11	Weak heart
G673200	Carotid artery dissection
C10F500	Type 2 diabetes mellitus with gangrene
G33z700	Stable angina
G668.00	Right sided CVA
G331.00	Prinzmetal's angina
662g.00	New York Heart Association classification - class II
G613.00	Cerebellar haemorrhage
G30..11	Attack - heart
G30..16	Thrombosis - coronary
G713.11	Ruptured abdominal aortic aneurysm
G67..00	Other cerebrovascular disease
G30z.00	Acute myocardial infarction NOS
G301z00	Anterior myocardial infarction NOS
G305.00	Lateral myocardial infarction NOS
G641.00	Cerebral embolism
G64z.11	Brainstem infarction NOS
G740.00	Embolism and thrombosis of the abdominal aorta
G715.00	Ruptured aortic aneurysm NOS
G310.11	Dressler's syndrome
G34z.00	Other chronic ischaemic heart disease NOS
G65zz00	Transient cerebral ischaemia NOS
G73z100	Spasm of peripheral artery
G716.00	Aortic aneurysm without mention of rupture NOS
G723100	Aneurysm of popliteal artery
G722000	Aneurysm of common iliac artery
G32..11	Healed myocardial infarction
G65z100	Intermittent cerebral ischaemia
G640.00	Cerebral thrombosis
G710.00	Dissecting aortic aneurysm
G711.11	Ruptured thoracic aortic aneurysm
G669.00	Cerebral palsy, not congenital or infantile, acute
G30A.00	Mural thrombosis
G58z.12	Cardiac failure NOS
G311200	Angina at rest

G664.00	Cerebellar stroke syndrome
G60X.00	Subarachnoid haemorrh from intracranial artery, unspecif
G714.11	AAA - Abdominal aortic aneurysm without mention of rupture
G32..12	Personal history of myocardial infarction
G722.00	Aneurysm of iliac artery
G30..17	Silent myocardial infarction
G622.00	Subdural haematoma - nontraumatic
G713.00	Abdominal aortic aneurysm which has ruptured
8HBE.00	Heart failure follow-up
G301100	Acute anteroseptal infarction
G311400	Worsening angina
G330000	Nocturnal angina
G721.00	Aneurysm of renal artery
G61..12	Stroke due to intracerebral haemorrhage
G660.00	Middle cerebral artery syndrome
G35..00	Subsequent myocardial infarction
662f.00	New York Heart Association classification - class I
G34z000	Asymptomatic coronary heart disease
G623.00	Subdural haemorrhage NOS
662h.00	New York Heart Association classification - class III
G61X100	Right sided intracerebral haemorrhage, unspecified
G662.00	Posterior cerebral artery syndrome
G661.00	Anterior cerebral artery syndrome
G65y.00	Other transient cerebral ischaemia
G602.00	Subarachnoid haemorrhage from middle cerebral artery
G311.14	Angina at rest
G330.00	Angina decubitus
G62z.00	Intracranial haemorrhage NOS
G3...12	Atherosclerotic heart disease
G651000	Vertebro-basilar artery syndrome
G232.00	Hypertensive heart&renal dis wth (congestive) heart failure
G31y300	Transient myocardial ischaemia
G673000	Dissection of cerebral arteries, nonruptured
G3y..00	Other specified ischaemic heart disease
G674.00	Cerebral arteritis
G34y100	Chronic myocardial ischaemia
G68..00	Late effects of cerebrovascular disease
G652.00	Subclavian steal syndrome
G581.11	Asthma - cardiac
G731000	Buerger's disease
G712.00	Thoracic aortic aneurysm without mention of rupture
G310.00	Postmyocardial infarction syndrome
G60z.00	Subarachnoid haemorrhage NOS
G63y000	Cerebral infarct due to thrombosis of precerebral arteries
G580000	Acute congestive heart failure
G361.00	Atrial septal defect/curr comp folow acut myocardal infarct
G73y100	Peripheral angiopathic disease EC NOS
G304.00	Posterior myocardial infarction NOS
G650.00	Basilar artery syndrome
G360.00	Haemopericardium/current comp folow acut myocard infarct

G671100	Chronic cerebral ischaemia
G63y100	Cerebral infarction due to embolism of precerebral arteries
8B29.00	Cardiac failure therapy
G34y000	Chronic coronary insufficiency
G3...11	Arteriosclerotic heart disease
G720100	Aneurysm of radial artery
G64z000	Brainstem infarction
G33z.00	Angina pectoris NOS
G64z400	Infarction of basal ganglia
G33z600	New onset angina
G72yA00	Aneurysm of hepatic artery
G341.11	Cardiac aneurysm
G711.00	Thoracic aortic aneurysm which has ruptured
G580200	Decompensated cardiac failure
G31..00	Other acute and subacute ischaemic heart disease
G582.00	Acute heart failure
G641000	Cerebral infarction due to embolism of cerebral arteries
G31yz00	Other acute and subacute ischaemic heart disease NOS
G74..13	Arterial embolic and thrombotic occlusion
G34..00	Other chronic ischaemic heart disease
G61X000	Left sided intracerebral haemorrhage, unspecified
G33zz00	Angina pectoris NOS
G30y000	Acute atrial infarction
G344.00	Silent myocardial ischaemia
G366.00	Thrombosis atrium,auric append&vent/curr comp foll acute MI
G303.00	Acute inferoposterior infarction
G30X.00	Acute transmural myocardial infarction of unspecif site
G330z00	Angina decubitus NOS
G600.00	Ruptured berry aneurysm
G616.00	External capsule haemorrhage
G617.00	Intracerebral haemorrhage, intraventricular
G720200	Aneurysm of ulnar artery
G309.00	Acute Q-wave infarct
G30..13	Cardiac rupture following myocardial infarction (MI)
662W.00	Heart failure annual review
G723500	Ruptured popliteal artery aneurysm
G61X.00	Intracerebral haemorrhage in hemisphere, unspecified
G610.00	Cortical haemorrhage
G677.00	Occlusion/stenosis cerebral arts not result cerebral infarct
G62..00	Other and unspecified intracranial haemorrhage
G672.11	Hypertensive crisis
G72y000	Aneurysm of common carotid art
G38..00	Postoperative myocardial infarction
G675.00	Moyamoya disease
C107.11	Diabetes mellitus with gangrene
G630.00	Basilar artery occlusion
G33z400	Ischaemic chest pain
C107.12	Diabetes with gangrene
G580100	Chronic congestive heart failure
G30B.00	Acute posterolateral myocardial infarction

8H2S.00	Admit heart failure emergency
G651.00	Vertebral artery syndrome
G665.00	Pure motor lacunar syndrome
G6X..00	Cerebrl infarctn due/unspcf occlusn or sten/cerebrl artrs
G720000	Aneurysm of brachial artery
C107200	Diabetes mellitus, adult with gangrene
G67y.00	Other cerebrovascular disease OS
G73y000	Diabetic peripheral angiopathy
G311300	Refractory angina
G34y.00	Other specified chronic ischaemic heart disease
G731.00	Thromboangiitis obliterans
G641.11	Cerebral embolus
G30y.00	Other acute myocardial infarction
G673100	Carotico-cavernous sinus fistula
G72y400	Aneurysm of subclavian artery
G34yz00	Other specified chronic ischaemic heart disease NOS
G620.00	Extradural haemorrhage - nontraumatic
G72y200	Aneurysm of internal carotid artery
G36..00	Certain current complication follow acute myocardial infarct
G311.00	Preinfarction syndrome
G342.00	Atherosclerotic cardiovascular disease
G640000	Cerebral infarction due to thrombosis of cerebral arteries
G332.00	Coronary artery spasm
G67z.00	Other cerebrovascular disease NOS
G362.00	Ventric septal defect/curr comp fol acut myocardal infarctn
C10FF00	Type 2 diabetes mellitus with peripheral angiopathy
G676.00	Nonpyogenic venous sinus thrombosis
G351.00	Subsequent myocardial infarction of inferior wall
G72y500	Aneurysm of splenic artery
G73y.00	Other specified peripheral vascular disease
G676000	Cereb infarct due cerebral venous thrombosis, nonpyogenic
G683.00	Sequelae of cerebral infarction
G312.00	Coronary thrombosis not resulting in myocardial infarction
Gyu3000	[X]Other forms of angina pectoris
G311.12	Impending infarction
G31y200	Subendocardial ischaemia
G671.00	Generalised ischaemic cerebrovascular disease NOS
G611.00	Internal capsule haemorrhage
C109500	Non-insulin dependent diabetes mellitus with gangrene
G301000	Acute anteroapical infarction
G723600	Post radiological femoral false aneurysm
G6W..00	Cereb infarct due unsp occlus/stenos precerebr arteries
G716000	Thoracoabdominal aortic aneurysm, without mention of rupture
G632.00	Vertebral artery occlusion
G72y.00	Aneurysm of other artery
G30y200	Acute septal infarction
G341z00	Aneurysm of heart NOS
G384.00	Postoperative subendocardial myocardial infarction
G605.00	Subarachnoid haemorrhage from basilar artery
G603.00	Subarachnoid haemorrhage from anterior communicating artery

G682.00	Sequelae of other nontraumatic intracranial haemorrhage
G581.12	Pulmonary oedema - acute
G680.00	Sequelae of subarachnoid haemorrhage
G653.00	Carotid artery syndrome hemispheric
G723.00	Aneurysm of leg artery
G714000	Juxtarenal aortic aneurysm
G63..00	Precerebral arterial occlusion
G350.00	Subsequent myocardial infarction of anterior wall
G30yz00	Other acute myocardial infarction NOS
G380.00	Postoperative transmural myocardial infarction anterior wall
C109512	Type 2 diabetes mellitus with gangrene
G35X.00	Subsequent myocardial infarction of unspecified site
G381.00	Postoperative transmural myocardial infarction inferior wall
G612.00	Basal nucleus haemorrhage
Gyu3300	[X]Other forms of chronic ischaemic heart disease
G64z100	Wallenberg syndrome
G681.00	Sequelae of intracerebral haemorrhage
G654.00	Multiple and bilateral precerebral artery syndromes
G72y100	Aneurysm of external carotid artery
G68W.00	Sequelae/other + unspecified cerebrovascular diseases
662i.00	New York Heart Association classification - class IV
G6y..00	Other specified cerebrovascular disease
G63y.00	Other precerebral artery occlusion
G740.13	Leriche's syndrome
G677000	Occlusion and stenosis of middle cerebral artery
G666.00	Pure sensory lacunar syndrome
Gyu3.00	[X]Ischaemic heart diseases
G72y600	Aneurysm of axillary artery
Gyu6400	[X]Other cerebral infarction
Gyu6200	[X]Other intracerebral haemorrhage
G311z00	Preinfarction syndrome NOS
G33z100	Stenocardia
C109F11	Type II diabetes mellitus with peripheral angiopathy
G311011	MI - myocardial infarction aborted
G65z000	Impending cerebral ischaemia
G677300	Occlusion and stenosis of cerebellar arteries
G601.00	Subarachnoid haemorrhage from carotid siphon and bifurcation
C107400	NIDDM with peripheral circulatory disorder
G72y900	Aneurysm of inferior mesenteric artery
G618.00	Intracerebral haemorrhage, multiple localized
G63..11	Infarction - precerebral
G677100	Occlusion and stenosis of anterior cerebral artery
G234.00	Hyperten heart&renal dis+both(congestv)heart and renal fail
G72y300	Aneurysm of neck artery NOS
G722200	Aneurysm of internal iliac artery
G363.00	Ruptur cardiac wall w'out haemopericard/cur comp fol ac MI
G341200	Aneurysm of coronary vessels
G720.00	Aneurysm of artery of arm
G72yB00	Aneurysm of other visceral artery
G72y800	Aneurysm of superior mesenteric artery

G722z00	Aneurysm of iliac artery NOS
G364.00	Ruptur chordae tendinae/curr comp fol acute myocard infarct
C108600	Insulin dependent diabetes mellitus with gangrene
G606.00	Subarachnoid haemorrhage from vertebral artery
C109F12	Type 2 diabetes mellitus with peripheral angiopathy
G722100	Aneurysm of external iliac artery
G311000	Myocardial infarction aborted
C109511	Type II diabetes mellitus with gangrene
G615.00	Bulbar haemorrhage
G30y100	Acute papillary muscle infarction
G723z00	Aneurysm of leg artery NOS
C107100	Diabetes mellitus, adult, + peripheral circulatory disorder
G306.00	True posterior myocardial infarction
SP11200	Cardiorespiratory failure as a complication of care
G63..12	Stenosis of precerebral arteries
G713000	Ruptured suprarenal aortic aneurysm
C108G00	Insulin dependent diab mell with peripheral angiopathy
C107z00	Diabetes mellitus NOS with peripheral circulatory disorder
Gyu6100	[X]Other subarachnoid haemorrhage
G677200	Occlusion and stenosis of posterior cerebral artery
SP11111	Heart failure as a complication of care
G33z000	Status anginosus
G72y700	Aneurysm of coeliac artery
G723200	Aneurysm of anterior tibial artery
G341100	Other cardiac wall aneurysm
G31y100	Microinfarction of heart
Gyu3200	[X]Other forms of acute ischaemic heart disease
G38z.00	Postoperative myocardial infarction, unspecified
C107300	IDDM with peripheral circulatory disorder
G365.00	Rupture papillary muscle/curr comp fol acute myocard infarct
G723300	Aneurysm of dorsalis pedis artery
C10E600	Type 1 diabetes mellitus with gangrene
C107000	Diabetes mellitus, juvenile +peripheral circulatory disorder
G671000	Acute cerebrovascular insufficiency NOS
G677400	Occlusion+stenosis of multiple and bilat cerebral arteries
G63z.00	Precerebral artery occlusion NOS
G723400	Aneurysm of posterior tibial artery
G353.00	Subsequent myocardial infarction of other sites
Gyu6.00	[X]Cerebrovascular diseases
Gyu7400	[X]Other specified peripheral vascular diseases
662p.00	Heart failure 6 month review
Gyu6500	[X]Occlusion and stenosis of other precerebral arteries
Gyu6300	[X]Cerebrl infarctn due/unspcf occlusn or sten/cerebrl artrs
G341300	Acquired atrioventricular fistula of heart
Gyu6600	[X]Occlusion and stenosis of other cerebral arteries
C10EG00	Type 1 diabetes mellitus with peripheral angiopathy
G720z00	Aneurysm of arm artery NOS
Gyu6G00	[X]Cereb infarct due unsp occlus/stenos precerebr arteries
G580400	Congestive heart failure due to valvular disease
Gyu6F00	[X]Intracerebral haemorrhage in hemisphere, unspecified

G5y4z00	Post cardiac operation heart failure NOS
Gyu3400	[X]Acute transmural myocardial infarction of unspecif site
G673300	Vertebral artery dissection
G733.00	Ischaemic foot
G679.00	Small vessel cerebrovascular disease
G633.00	Multiple and bilateral precerebral arterial occlusion
Gyu3600	[X]Subsequent myocardial infarction of unspecified site
G583.11	HFNEF - heart failure with normal ejection fraction
G583.00	Heart failure with normal ejection fraction
G714300	Aneurysm of suprarenal aorta
G714200	Infrarenal abdominal aortic aneurysm
G67A.00	Cerebral vein thrombosis
G73z012	Vascular claudication
C10E611	Type I diabetes mellitus with gangrene
C10F511	Type II diabetes mellitus with gangrene
C10FF11	Type II diabetes mellitus with peripheral angiopathy
679W100	Education about deteriorating heart failure
G341111	Mural cardiac aneurysm
G734.00	Peripheral arterial disease
G39..00	Coronary microvascular disease
G657.00	Carotid territory transient ischaemic attack
661M500	Heart failure self-management plan agreed
8HTL000	Referral to rapid access heart failure clinic
G383.00	Postoperative transmural myocardial infarction unspec site
G583.12	Heart failure with preserved ejection fraction
G619.00	Lobar cerebral haemorrhage
G67B.00	Reversible cerebral vasoconstriction syndrome
G67B.11	Call-Fleming syndrome
Gyu6E00	[X]Subarachnoid haemorrh from intracranial artery, unspecif
Gyu6000	[X]Subarachnoid haemorrhage from other intracranial arteries
Gyu3500	[X]Subsequent myocardial infarction of other sites
C10E612	Insulin dependent diabetes mellitus with gangrene
Gyu6C00	[X]Sequelae of stroke,not specfd as h'morrhage or infarction
Gyu6700	[X]Other specified cerebrovascular diseases
2JZ..00	On optimal heart failure therapy
C107y00	Other specified diabetes mellitus with periph circ comps
Gyu3100	[X]Other current complicatns following acute myocard infarct
G721000	Acquired renal artery aneurysm
661N500	Heart failure self-management plan review
C108612	Type 1 diabetes mellitus with gangrene
